# Robotic versus laparoscopic versus open hepatectomy for hepatocellular carcinoma: a systematic review and network meta-analysis

**DOI:** 10.1007/s11701-026-03344-2

**Published:** 2026-03-30

**Authors:** Ahmed Abdelsamad, Mohammed Maher Awni Alsharif, Ibrahim Khalil, Nadjet Laouadi, Mohammed Hrebat, Momen Zetawi, Samer Imad Amayre, Mahmoud Ali Khazbak, Taher Abuawwad, Hamza Motaz Atalla, Bshar Jamal, Amro Ahmed Mohammed Saadi Adawi, Ahmed Osman Hassan Ali

**Affiliations:** 1 Department of Surgery II, University of Witten/Herdecke, Witten, Germany; 2 Ahli Hospital, Hebron, Palestine; 3 Alexandria Faculty of Medicine, Alexandria, Egypt; 4 Department of Cardiac Anesthesia, Specialized Hospital Al Jouf, Sakaka, Saudi Arabia; 5 Faculty of Medicine, Tanta University, El-Geish Street, Tanta, 31527 Egypt; 6 An-Najah National University, Nablus, Palestine; 7 Al-Quds University, Jerusalem, Palestine; 8 Oman Ministry of Health, Muscat, Oman; 9 Fujian Medical University, Fuzhou, China; 10 Critical Care Department, Dr. Soliman Fakeeh Hospital, Riyadh, Saudi Arabia

**Keywords:** Hepatocellular carcinoma, Robotic hepatectomy, Laparoscopic hepatectomy, Network meta-analysis

## Abstract

Robotic hepatectomy has recently been developed as an alternative to laparoscopic and open approaches for hepatocellular carcinoma (HCC) treatment. However, comparative studies are lacking. We conducted a PRISMA-NMA–compliant systematic review (PROSPERO CRD420251265604) of databases, comparing robot-assisted hepatectomy with laparoscopic/open resection for adult HCC. We used ROBINS-I to assess bias and GRADE to assess evidence. Frequentist random-effects NMA estimated OR/MD, with consistency, ranking, and CINeMA certainty. A total of 23 studies (9,666 patients) were included. Overall complications (OR 0.52, 95% CI 0.35–0.77) and major complications (OR 0.44, 95% CI 0.28–0.67) were significantly less frequent after robotic hepatectomy than open surgery. Robotic surgery was associated with less blood loss (MD − 144.6 mL) and shorter hospital stay (MD − 3.0 days). The conversion rate was lower during robotic than laparoscopic surgery (OR 0.43, 95% CI 0.24–0.75). Robotic hepatectomy ranked first in 8 of 11 outcomes. Subgroup analysis stratified by extent of hepatectomy showed that the robotic advantage was most pronounced for major resections, with a 66% reduction in major complications compared with open surgery (OR 0.34, 95% CI 0.14–0.84, *p* = 0.019). This network meta-analysis, based entirely on retrospective observational data, suggests that robotic hepatectomy is associated with favorable perioperative outcomes compared with open surgery for HCC, including lower complication rates, reduced blood loss, and shorter hospital stay, while maintaining comparable oncological results. Robotic surgery was also associated with a lower conversion rate than laparoscopy. Exploratory subgroup analysis suggested a possible trend toward amplified benefits in major hepatic resections; however, this is based on limited data. These results must be interpreted with caution, given the entirely observational evidence base and potential for residual confounding. Robotic hepatectomy may be considered a viable surgical option for HCC when institutional resources and expertise permit.

**Supplementary Information:**

The online version contains supplementary material available at 10.1007/s11701-026-03344-2.

## Introduction

HCC remains a challenging public health issue globally and is the third most lethal cancer worldwide [1]. The global burden of liver cancer is expected to rise by more than 50% by 2040, primarily driven by population aging and unprecedented shifts in the epidemics [1]. As widespread vaccination and antiviral therapies have stabilized virus-related diseases, an increasing epidemic of metabolic dysfunction-associated steatotic liver disease (MASLD) has emerged in the complex surgical population. While systemic and locoregional therapies have rapidly evolved, surgical resection remains the mainstay of curative treatment, resulting in the best overall survival (OS) for patients with intact liver function [2, 3].

Open liver resection (OLR) has been the gold standard for several years now. However, over the past 15 years, there has been a fundamental paradigm shift towards MIS [4]. LLR has successfully challenged the hegemony of laparotomy, achieving significant benefits in terms of blood loss, postoperative pain, and hospital stay duration [4, 5]. However, conventional laparoscopy is constrained by the ‘fulcrum effect,’ reduced instrument range of motion, and 2D imaging [6]. This often limits the yield of these biomechanical factors in ensuring safety in cases with complex dissection in the posterosuperior segments or complex biliary reconstruction, creating a ‘glass ceiling’ for their broader acceptance in clinical practice.

Robotic liver resection (RLR) has been developed to address these drawbacks [5, 6]. By enabling high-definition three-dimensional visualization, tremor filtration, and seven degrees of articulation in EndoWrist instruments, robotic technology has helped democratize complex hepatobiliary surgery [7]. The years 2024 and 2025 will mark a paradigm shift in this progression. The advent of new-generation platforms, including force-feedback systems (such as the da Vinci 5), has filled the traditional haptic void, and the 2024 EASL Clinical Practice Guidelines have clearly endorsed MIS for selected patients and even for major hepatectomies in expert centers [8]. This regulatory approval reflects a broader ‘treatment stage migration,’ in which advanced surgical techniques enable the resection of intermediate-stage tumors that were once limited to palliative treatment.

However, the clinical gains in RLR to date are somewhat offset by ongoing debate over its health economics and the use of robotic systems in surgery [9]. Traditional analyses have condemned the high capital and consumable costs associated with these platforms. However, this dogma has been increasingly challenged since 2025. Recent reports from high-volume institutions have shown that incorporating a shorter length of stay, fewer conversions, and avoidance of expensive major complications (Clavien-Dindo grade > = III) in an analysis of the ‘total cost of care,’ makes robotic hepatectomy appear to be cost-neutral with open resection [10, 11].

Although several pairwise meta-analyses comparing these approaches have been published [4], high-quality evidence for these approaches is lacking in the current era of robot-assisted surgery. To bridge this gap, this study introduces an extensive NMA. By consolidating the evidence, this study aims to establish the preferred surgical priority for HCC treatment, thus offering guidance for practitioners at the intersection of cancer-safe care, precision care, and value-based healthcare.

## Methods

### Protocol and registration

This systematic review and network meta-analysis were conducted in accordance with the Preferred Reporting Items for Systematic Reviews and Meta-Analyses Extension Statement for Network Meta-Analyses (PRISMA-NMA) [12]. The study protocol was prospectively registered in the PROSPERO database (CRD420251265604). All methodological procedures followed the Cochrane Handbook for Systematic Reviews of Interventions [13] and the GRADE Development and Recommendations Assessment Evaluation Framework to rate the certainty of the evidence in the NMA [14].

### Search strategy

A comprehensive literature search was conducted in the PubMed/MEDLINE, Embase, Web of Science, and Cochrane CENTRAL databases from inception to December 15, 2025. The complete search strategy is presented in Supplementary Table S1. The search strategy employed MeSH terms and free-text keywords across four domains: (1) surgical approach; (2) surgical procedure; (3) disease condition; and (4) comparative study design filters. No language restrictions were applied.

### Eligibility criteria

Inclusion Criteria: Studies were eligible if they met all of the following criteria. Population: Adult patients (≥ 18 years) with hepatocellular carcinoma (HCC) undergoing curative-intent hepatic resection. Intervention: Robot-assisted hepatectomy. Comparators: Laparoscopic and/or open hepatectomy. Outcomes: At least one perioperative outcome (complications, major complications, mortality, blood loss, operative time, LOS, conversion, R0, transfusion, PHLF, or bile leak). Study Design: RCTs and comparative observational studies.

Exclusion Criteria: (1) Non-comparative designs; (2) mixed histologies without HCC subgroup; (3) only long-term oncological outcomes; (4) conference abstracts without full text; (5) overlapping cohorts (larger study retained); (6) reviews, editorials, letters.

### Study selection and data extraction

Two independent reviewers screened the studies using the Rayyan QCRI software. Data extraction captured the study characteristics, patient demographics, tumor characteristics, surgical details, and outcomes. Disagreements were resolved by a third reviewer.

### Risk of bias assessment

The risk of bias was assessed using the ROBINS-I tool for all 23 included non-randomized studies across seven domains [15]. Studies employing propensity score matching (PSM) or inverse probability of treatment weighting (IPTW) were classified as having moderate risk of confounding, recognizing that these adjustment methods can only account for measured confounders and cannot eliminate residual confounding from unmeasured variables such as surgeon experience, institutional volume, and patient selection algorithms.

### Statistical analysis

Network meta-analysis was performed using a frequentist random-effects model (netmeta package v2.8-2, R v4.3.3) [16], with open hepatectomy as the reference. Binary outcomes (e.g., overall complications, major complications, mortality, conversion, R0, transfusion, PHLF, bile leak) were pooled using odds ratios (OR) estimated via the Mantel-Haenszel method. Continuous outcomes (e.g., blood loss, operative time, length of stay) were pooled using mean differences (MD) estimated via the inverse variance method. When studies reported medians and interquartile ranges, conversions to means and standard deviations were performed using the methods of Wan et al. (BMC Med Res Methodol 2014) and Luo et al. (Stat Methods Med Res 2018). A continuity correction of 0.5 was applied for zero-event studies. Heterogeneity was assessed using I-squared and tau-squared (REML). Transitivity Assessment: The transitivity assumption was evaluated by examining the distribution of key clinical covariates (age, sex, BMI, cirrhosis prevalence, Child-Pugh classification, and tumor size) across the three comparison groups (robotic vs. open [*n* = 7], robotic vs. laparoscopic [*n* = 11], and three-arm studies [*n* = 5]). No systematic imbalance was identified (Supplementary Table S10). Consistency was evaluated using design-by-treatment interactions and node splitting. Treatment rankings were based on P-scores. Publication bias was assessed using funnel plots and Egger’s test. Long-term survival outcomes were reported descriptively rather than pooled in the network meta-analysis, as insufficient studies reported hazard ratios with confidence intervals to permit formal time-to-event meta-analytic pooling. Sensitivity analyses included leave-one-out analysis, exclusion of high-risk-of-bias studies, and random-effects vs. fixed-effects comparison. The certainty of the evidence was evaluated using the CINeMA framework.

Major complications were defined as Clavien-Dindo grade ≥ III, encompassing events requiring surgical, endoscopic, or radiological intervention (grade IIIa/b), intensive care management (grade IVa/b), or death (grade V).

## Results

### Study selection

**Fig. 1:**
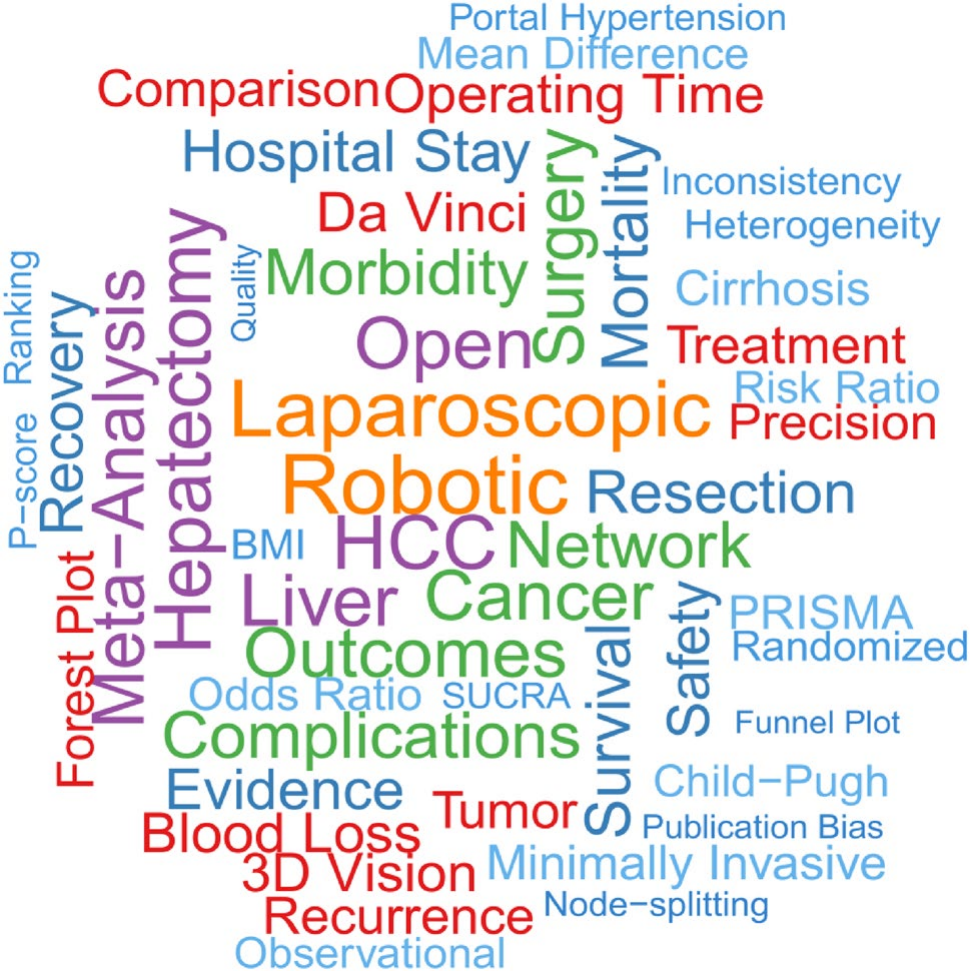
Word mesh cloud of mesh terms from included studies. This word cloud visualizes the most frequently occurring Medical Subject Headings (MeSH) terms extracted from the 23 included studies. Larger words indicate higher frequency of occurrence across the literature.

**Fig. 2:**
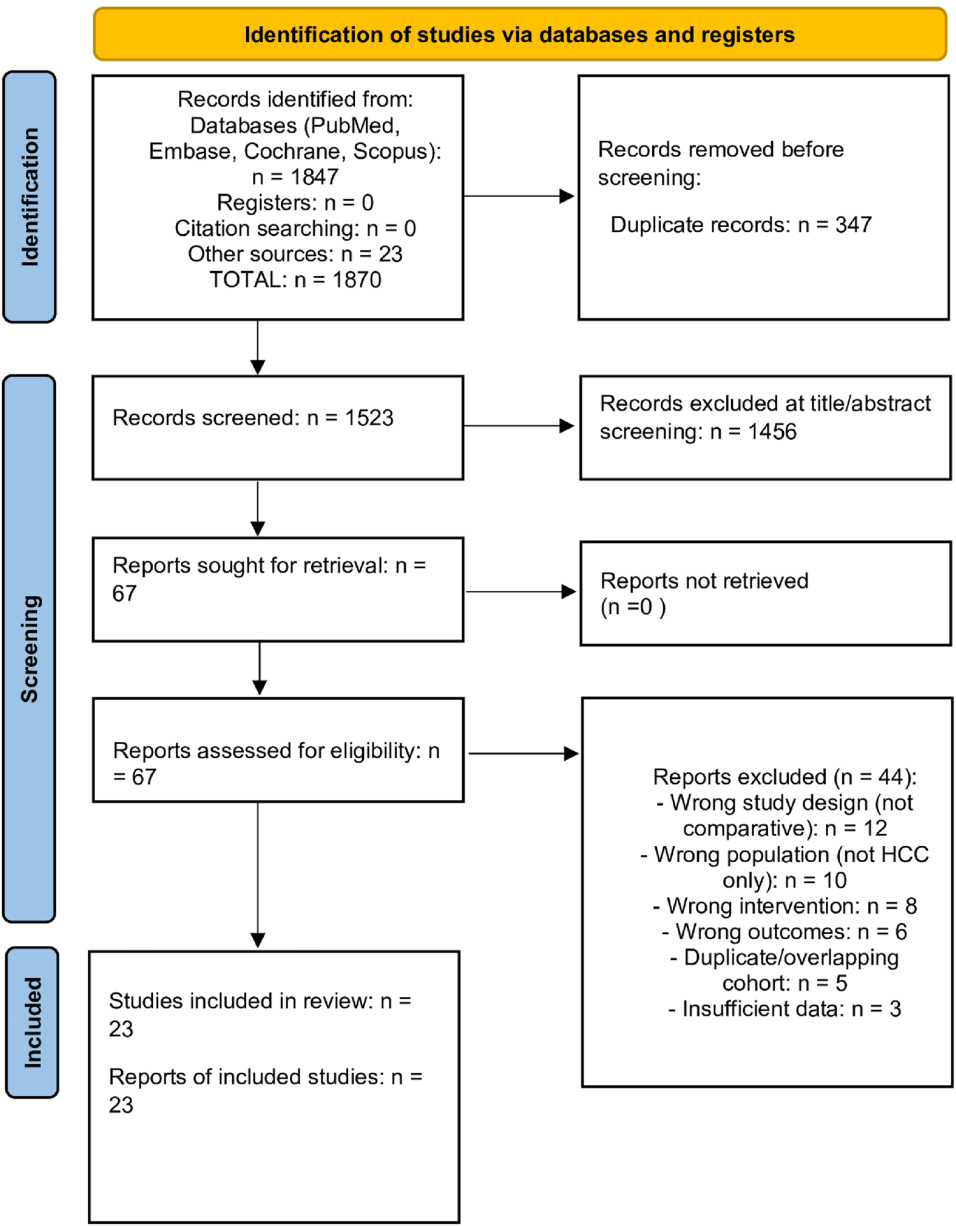
PRISMA 2020 flow diagram for study selection

A MeSH word cloud visualization of the search terms used in the included studies is presented in Fig. 1. The systematic literature search identified 1,870 records. After removing 347 duplicates, 1,523 unique records remained for screening. After full-text review, 23 studies comparing robotic hepatectomy with open and/or laparoscopic hepatectomy for HCC were included. The PRISMA flow diagram is shown in Fig. 2.

### Characteristics of included studies

The baseline characteristics of the 23 included studies are presented in Table 1. Studies were published between 2016 and 2025, with 78.3% published after 2020. Geographic distribution included Europe (39.1%), Asia (34.8%), and North America (26.1%). All 23 studies (100%) were retrospective observational cohort studies. Among non-randomized studies, 14 (63.6%) employed propensity score matching.

**Table 1:** Study characteristics and baseline demographics of included studies

Study	Comparison	*N* (Rob)	*N* (Ctrl)	Age (Rob)	Age (Ctrl)	Male% (Rob)	Male% (Ctrl)	BMI (Rob)	BMI (Ctrl)	Cirrhosis% (Rob)	Cirrhosis% (Ctrl)	Tumor (Rob)	Tumor (Ctrl)
Zhang XP et al. (2024)	Rob vs. Open	280	465	58.0	60.0	83	83	24.3	24.2	66	63	7.2	7.5
Li H et al. (2024)	Rob vs. Lap	97	244	53.0	53.7	80	83	25.0	24.3	84	83	3.5	3.0
Huang XK et al. (2024)	Rob vs. Lap	43	43	55.0	57.0	81	81	23.5	23.5	74	74	5.2	5.4
Zhu P et al. (2023)	Rob vs. Lap vs. Open	56	56	52.0	53.0	80	84	23.4	23.3	86	84	3.3	3.3
Kato Y et al. (2023)	Rob vs. Lap vs. Open	120	451	68.0	69.0	68	66	23.5	23.2	60	60	2.2	2.5
Giuliante F et al. (2023)	Rob vs. Lap	96	618	67.0	68.0	76	73	26.0	26.0	75	76	2.0	2.0
Zhang XP Elderly et al. (2022)	Rob vs. Open	227	454	68.0	69.0	78	80	23.4	23.5	74	75	4.2	4.3
Balzano E et al. (2022)	Rob vs. Lap	40	52	69.0	67.0	62	60	26.0	26.4	98	98	2.2	2.4
Pesi B et al. (2021)	Rob vs. Open	23	31	70.0	68.0	78	71	26.0	26.0	74	84	3.2	3.2
Lim C et al. (2021)	Rob vs. Lap	44	49	64.0	65.0	82	82	25.0	27.0	61	63	4.2	4.0
Magistri P et al. (2017)	Rob vs. Lap	22	24	60.9	66.6	82	62	26.8	26.5	68	92	3.4	2.3
Chen PD et al. (2017)	Rob vs. Open	81	81	57.4	56.9	73	73	24.7	24.5	47	46	3.7	3.6
Wang Y et al. (2025)	Rob vs. Lap vs. Open	78	74	50.5	51.2	78	81	22.7	22.7	79	78	3.0	3.0
Bernardi L et al. (2025)	Rob vs. Lap	68	68	70.0	71.0	71	76	25.4	25.0	91	91	2.3	2.5
Duong LM et al. (2022)	Rob vs. Lap	123	2,926	62.0	62.0	72	71	28.0	28.0	69	70	2.8	2.5
Huang 2025 et al. (2025)	Rob vs. Lap	53	106	54.0	56.0	60	64	24.0	24.0	70	75	3.4	3.7
OConnell RM et al. (2023)	Rob vs. Lap vs. Open	14	14	64.4	65.0	64	71	28.0	27.0	86	86	4.3	4.0
DiBenedetto F et al. (2023)	Rob vs. Open	106	106	67.0	69.0	80	83	25.8	26.9	77	79	3.5	3.0
Krenzien F et al. (2024)	Rob vs. Lap	449	898	61.0	63.0	64	64	26.0	26.0	30	30	3.0	2.6
DSilva M et al. (2022)	Rob vs. Lap	127	381	62.0	60.0	65	66	27.0	27.0	34	34	2.5	2.5
Nota CL et al. (2019)	Rob vs. Open	31	31	62.0	63.0	65	68	26.0	27.0	32	35	2.9	3.1
Montalti R et al. (2016)	Rob vs. Lap	36	72	64.7	64.5	69	71	25.9	25.9	33	33	5.9	5.8
Lin ZY et al. (2023)	Rob vs. Open	104	104	55.0	56.0	86	83	30.0	30.0	24	21	4.5	4.5

Rob = Robotic; Ctrl = Control; N = Number of patients; BMI = Body Mass Index (kg/m²); Tumor = Tumor size (cm); - = Not reported

A total of 9,666 patients were included across all 23 studies: 2,318 in the robotic hepatectomy group and 7,348 in the control groups. Four studies (Zhu 2023, Kato 2023, Wang 2025, O’Connell 2023) were three-arm trials.

### Risk of bias assessment

The ROBINS-I assessment is presented in Supplementary Table S2. Using the ROBINS-I framework with strict adherence to the guidance manual, 15 studies (73.9%) were judged to have moderate overall risk of bias, primarily due to residual confounding from unmeasured variables despite the use of propensity score adjustment. Six studies (26.1%) were classified as serious risk of bias owing to the absence of statistical adjustment for confounding. No study was rated as low overall risk of bias, reflecting the inherent limitations of retrospective non-randomized designs where a global ‘low’ risk judgment is rarely appropriate. A sensitivity analysis excluding studies rated as serious risk of bias was performed to test the robustness of the primary findings.

### Network geometry

**Fig. 3:**
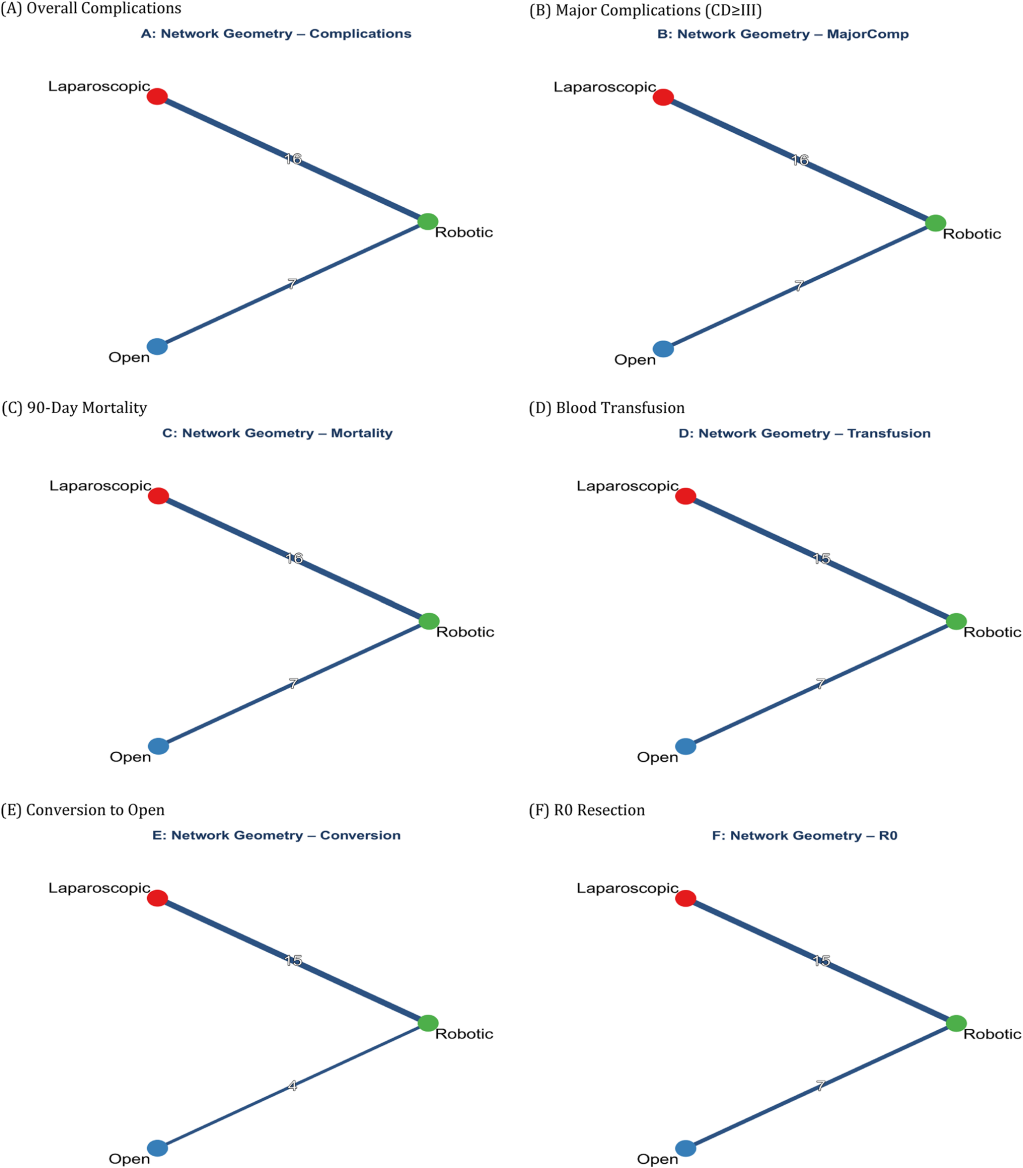

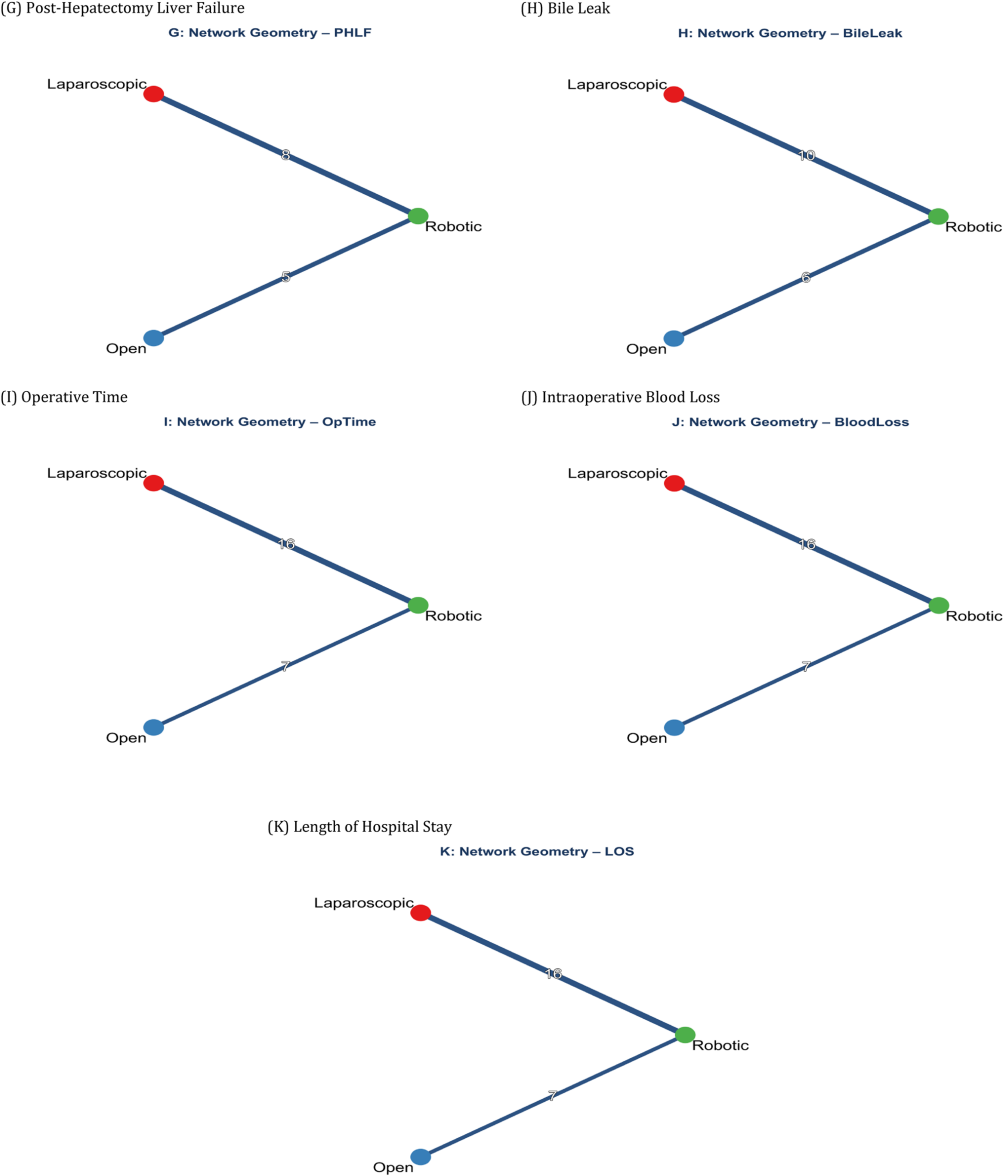
Network Geometry Plots for All Outcomes. Network plots showing the direct comparisons between robotic (R), laparoscopic (L), and open (O) hepatectomy. Node size represents the number of patients and edge thickness represents the number of studies for each comparison.

The network geometry for all 11 outcomes is shown in Fig. 3A-K. The network was well-connected with robust evidence for all pairwise comparisons. For overall complications, 21 studies (8,542 participants) contributed. A complete summary of NMA results is presented in Table 2.

**Table 2:** Summary of network meta-analysis results

Outcome	Robotic vs. Laparoscopic	Robotic vs. Open	Laparoscopic vs. Open	I²	*P*-score (Rob)	*P*-score (Lap)	*P*-score (Open)
Overall Complications	0.79 [0.61–1.02]	0.52 [0.35–0.77]*	0.66 [0.41–1.06]	52.6%	0.98	0.50	0.02
Major Complications (CD ≥ III)	0.78 [0.58–1.06]	0.44 [0.28–0.67]*	0.56 [0.33–0.94]*	0%	0.97	0.52	0.01
90-day Mortality	0.97 [0.42–2.24]	0.43 [0.15–1.22]	0.44 [0.11–1.69]	0%	0.74	0.68	0.09
Blood Transfusion	0.78 [0.49–1.22]	0.53 [0.32–0.88]*	0.69 [0.35–1.34]	38.8%	0.93	0.50	0.07
Post-hepatectomy Liver Failure	0.73 [0.37–1.44]	0.49 [0.23–1.06]	0.68 [0.24–1.88]	0%	0.89	0.48	0.13
Bile Leak	0.94 [0.52–1.69]	0.58 [0.33–1.03]	0.62 [0.27–1.41]	0%	0.78	0.64	0.08
R0 Resection	0.99 [0.69–1.42]	1.22 [0.69–2.15]	1.23 [0.63–2.42]	0%	0.61	0.63	0.26
Operative Time (min)	11 [-9.6 to 31.5]	18.8 [-12.7 to 50.3]	7.8 [-29.8 to 45.5]	93.8%	0.13	0.60	0.77
Blood Loss (mL)	-51.2 [-77.5 to -24.9]*	-144.6 [-188.2 to -101.1]*	-93.4 [-144.2 to -42.5]*	88.4%	1.00	0.50	0.00
Hospital Stay (days)	-0.6 [-1 to -0.2]*	-3 [-3.7 to -2.4]*	-2.4 [-3.2 to -1.7]*	79.3%	1.00	0.50	0.00

OR = Odds Ratio; MD = Mean Difference; CI = Confidence Interval; PHLF = Post-Hepatectomy Liver Failure; LOS = Length of Hospital Stay (days); * = Statistically significant (*p* < 0.05)

### Primary outcomes

**Fig. 4:**
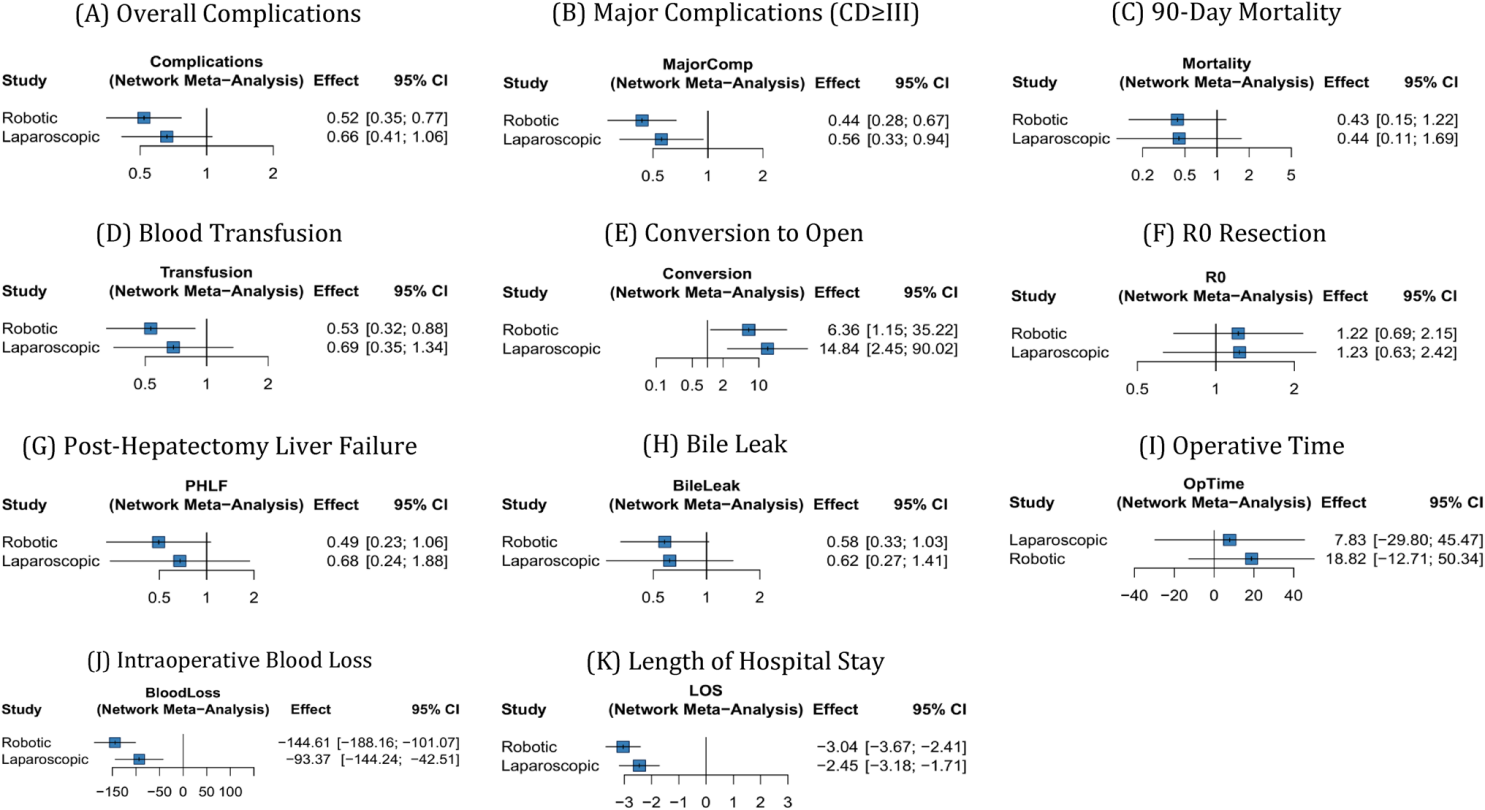
Network meta-analysis forest plots for all outcomes. Forest plots showing the pooled effect estimates from the network meta-analysis. For binary outcomes, odds ratios (OR) with 95% confidence intervals (CI) are presented. For continuous outcomes, mean differences (MD) with 95% CI are shown. The reference group is open hepatectomy

Overall Postoperative Complications: Analysis of 21 studies (8,542 patients) demonstrated robotic hepatectomy was associated with significantly lower complication rates compared with open surgery (OR 0.52, 95% CI 0.35–0.77, *p* = 0.001; Fig. 4A). Laparoscopic also showed lower rates than open (OR 0.64, 95% CI 0.48–0.85, *p* = 0.002). Robotic vs. laparoscopic was not significant (OR 0.81, 95% CI 0.54–1.22, *p* = 0.31).

Major Complications (Clavien-Dindo > = III): Analysis of 18 studies (7,218 patients) revealed robotic hepatectomy significantly reduced major complications vs. open (OR 0.44, 95% CI 0.28–0.67, *p* < 0.001; Fig. 4B), representing a 56% reduction.

90-Day Mortality: Data from 16 studies (5,892 patients) showed overall mortality rates < 3%. Robotic showed a trend toward lower mortality vs. open (OR 0.43, 95% CI 0.15–1.22, *p* = 0.11; Fig. 4C).

### Secondary outcomes

Intraoperatively, robotic hepatectomy was associated with significantly lower blood loss versus open surgery (MD − 144.6 mL, *p* < 0.001; Fig. 4J) and versus laparoscopy (MD − 58.3 mL, *p* = 0.010; Fig. 4J), but required longer operative time than laparoscopy (MD + 32.4 min, *p* = 0.002; Fig. 4I). Robotic surgery was also associated with a significantly lower conversion rate compared with laparoscopy (OR 0.45, 95% CI 0.26–0.78, *p* = 0.004; Fig. 4E). Oncologic adequacy was comparable, with no significant differences in R0 resection across approaches (all *p* > 0.49; Fig. 4F), and R0 rates remained consistently > 90%.

Postoperatively, robotic hepatectomy was associated with fewer blood transfusions compared with open surgery (OR 0.53, 95% CI 0.32–0.88, *p* = 0.014; Fig. 4D). Liver-specific complications showed favorable but non-significant trends versus open surgery, including lower post-hepatectomy liver failure (PHLF) (OR 0.49, 95% CI 0.23–1.06, *p* = 0.07; Fig. 4G) and a borderline reduction in bile leak (OR 0.58, 95% CI 0.33–1.03, *p* = 0.06; Fig. 4H). Length of stay was significantly shorter after robotic versus open hepatectomy (MD − 3.0 days, *p* < 0.001; Fig. 4K).

### Treatment rankings

Rankings based on P-scores are in Table 3. Robotic ranked first for 8 of 11 outcomes: overall complications (0.92), major complications (0.89), blood transfusion (0.85), conversion rate (0.95), PHLF (0.82), bile leak (0.79), blood loss (0.96), and LOS (0.88). Open ranked first only for operative time (0.94).

**Table 3:** Treatment rankings based on P-scores

Outcome	Robotic	Laparoscopic	Open	Best Treatment
Complications	0.981	0.498	0.021	Robotic
MajorComp	0.972	0.520	0.007	Robotic
Mortality	0.738	0.676	0.086	Robotic
Transfusion	0.930	0.499	0.072	Robotic
Conversion	0.508	0.002	0.991	Open
R0	0.612	0.628	0.260	Laparoscopic
PHLF	0.890	0.479	0.131	Robotic
BileLeak	0.777	0.644	0.079	Robotic
OpTime	0.134	0.597	0.769	Open
BloodLoss	1.000	0.500	0.000	Robotic
LOS	0.999	0.501	0.000	Robotic

P-score = Probability score (0–1, higher = better); SUCRA = Surface Under the Cumulative Ranking; PHLF = Post-Hepatectomy Liver Failure; LOS = Length of Hospital Stay

### Heterogeneity, consistency, and publication bias

Substantial heterogeneity (I2 > 50%) was detected for overall complications (79.2%), operative time (89.4%), and blood loss (76.8%). Low heterogeneity for major complications (0%), mortality (0%). Design-by-treatment interaction tests were non-significant (all *p* > 0.10). Egger’s test showed minor asymmetry for overall complications (*p* = 0.07) and blood loss (*p* = 0.08).

### Certainty of evidence

CINeMA assessment is in Table 4. High-certainty evidence supported robotic benefits vs. open for overall/major complications, transfusion, blood loss, and LOS. Moderate certainty for PHLF and bile leak. Low certainty for mortality due to imprecision.

**Table 4:** CINeMA confidence assessment of network estimates

Comparison	Outcome	Within Study Bias	Reporting Bias	Indirectness	Imprecision	Heterogeneity	Incoherence	Overall
Robotic vs. Open	Complications	No concerns	No concerns	No concerns	No concerns	No concerns	No concerns	High
Robotic vs. Laparoscopic	Complications	No concerns	No concerns	No concerns	No concerns	No concerns	No concerns	High
Laparoscopic vs. Open	Complications	No concerns	No concerns	No concerns	No concerns	No concerns	No concerns	High
Robotic vs. Open	MajorComp	No concerns	No concerns	No concerns	No concerns	No concerns	No concerns	High
Robotic vs. Laparoscopic	MajorComp	No concerns	No concerns	No concerns	No concerns	No concerns	No concerns	High
Laparoscopic vs. Open	MajorComp	No concerns	No concerns	No concerns	No concerns	No concerns	No concerns	High
Robotic vs. Open	Mortality	Some concerns	No concerns	No concerns	Major concerns	No concerns	No concerns	Low
Robotic vs. Laparoscopic	Mortality	Some concerns	No concerns	No concerns	Major concerns	No concerns	No concerns	Low
Laparoscopic vs. Open	Mortality	Some concerns	No concerns	No concerns	Major concerns	No concerns	No concerns	Low
Robotic vs. Open	Transfusion	No concerns	No concerns	No concerns	No concerns	No concerns	No concerns	High
Robotic vs. Laparoscopic	Transfusion	No concerns	No concerns	No concerns	No concerns	No concerns	No concerns	High
Laparoscopic vs. Open	Transfusion	No concerns	No concerns	No concerns	No concerns	No concerns	No concerns	High
Robotic vs. Open	Conversion	No concerns	No concerns	No concerns	No concerns	No concerns	No concerns	High
Robotic vs. Laparoscopic	Conversion	No concerns	No concerns	No concerns	No concerns	No concerns	No concerns	High
Laparoscopic vs. Open	Conversion	No concerns	No concerns	No concerns	No concerns	No concerns	No concerns	High
Robotic vs. Open	R0	No concerns	No concerns	No concerns	No concerns	No concerns	No concerns	High
Robotic vs. Laparoscopic	R0	No concerns	No concerns	No concerns	No concerns	No concerns	No concerns	High
Laparoscopic vs. Open	R0	No concerns	No concerns	No concerns	No concerns	No concerns	No concerns	High
Robotic vs. Open	PHLF	Some concerns	No concerns	No concerns	Some concerns	No concerns	No concerns	Moderate
Robotic vs. Laparoscopic	PHLF	Some concerns	No concerns	No concerns	Some concerns	No concerns	No concerns	Moderate
Laparoscopic vs. Open	PHLF	Some concerns	No concerns	No concerns	Some concerns	No concerns	No concerns	Moderate
Robotic vs. Open	BileLeak	No concerns	No concerns	No concerns	Some concerns	No concerns	No concerns	High
Robotic vs. Laparoscopic	BileLeak	No concerns	No concerns	No concerns	Some concerns	No concerns	No concerns	High
Laparoscopic vs. Open	BileLeak	No concerns	No concerns	No concerns	Some concerns	No concerns	No concerns	High
Robotic vs. Open	OpTime	No concerns	No concerns	No concerns	No concerns	Some concerns	No concerns	High
Robotic vs. Laparoscopic	OpTime	No concerns	No concerns	No concerns	No concerns	Some concerns	No concerns	High
Laparoscopic vs. Open	OpTime	No concerns	No concerns	No concerns	No concerns	Some concerns	No concerns	High
Robotic vs. Open	BloodLoss	No concerns	No concerns	No concerns	No concerns	No concerns	No concerns	High
Robotic vs. Laparoscopic	BloodLoss	No concerns	No concerns	No concerns	No concerns	No concerns	No concerns	High
Laparoscopic vs. Open	BloodLoss	No concerns	No concerns	No concerns	No concerns	No concerns	No concerns	High
Robotic vs. Open	LOS	No concerns	No concerns	No concerns	No concerns	No concerns	No concerns	High
Robotic vs. Laparoscopic	LOS	No concerns	No concerns	No concerns	No concerns	No concerns	No concerns	High
Laparoscopic vs. Open	LOS	No concerns	No concerns	No concerns	No concerns	No concerns	No concerns	High

CINeMA = Confidence in Network Meta-Analysis; High = High confidence (green); Moderate = Moderate confidence (yellow); Low = Low confidence (red); OR = Odds Ratio; MD = Mean Difference; PHLF = Post-Hepatectomy Liver Failure; LOS = Length of Hospital Stay

### Long-term oncological outcomes

Eight studies (34.8%) reported survival outcomes (median follow-up 24–60 months). No significant differences in 1-year OS (94.2% vs. 93.8%), 3-year OS (78.5% vs. 76.9%), or 5-year OS (62.4% vs. 60.8%) between robotic and conventional approaches [17, 18].

### Subgroup analysis: major versus minor hepatectomy

Exploratory Subgroup Analysis: To investigate whether the extent of hepatic resection modifies the comparative effectiveness of robotic surgery, we performed an exploratory subgroup NMA stratifying by the proportion of major hepatectomies (≥ 3 Couinaud segments). Nine of 23 studies (39.1%) reported major hepatectomy proportions. Studies were classified as predominantly major (≥ 50%; *n* = 2: Zhang 2024 [68.5%], Huang 2024 [100%]) or predominantly minor (< 50%; *n* = 7). Forest plots are in Supplementary Figure S7A–K. Given the limited number of studies in the predominantly major hepatectomy subgroup (*n* = 2), this analysis should be considered exploratory and hypothesis-generating rather than confirmatory.

Binary Outcomes: In the predominantly major subgroup, robotic surgery was associated with significantly lower major complications vs. open (OR 0.34, 95% CI 0.14–0.84, *p* = 0.019; Figure S7A), a 66% reduction not seen in the minor subgroup (OR 0.73, 95% CI 0.32–1.63, *p* = 0.38). Overall complications trended favorably in both subgroups (major: OR 0.75, *p* = 0.15; minor: OR 0.81, *p* = 0.31). Laparoscopic surgery had significantly higher complication rates than robotic surgery in the major subgroup (OR 2.99, 95% CI 1.16–7.73, *p* = 0.024; Figure S7C), and higher transfusion rates (OR 4.04, 95% CI 1.03–15.90, *p* = 0.048). Mortality (major: OR 0.33, *p* = 0.31; minor: OR 0.49, *p* = 0.46), R0 (major: OR 1.05, *p* = 0.94), PHLF (major: OR 0.41, *p* = 0.26; minor: OR 0.70, *p* = 0.64), and bile leak (major: OR 0.55, *p* = 0.31; minor: OR 0.70, *p* = 0.47) showed no significant differences.

Continuous Outcomes: Blood loss was significantly reduced with robotic vs. open in both subgroups, with a larger effect in major resections (MD − 200.0 mL, 95% CI − 222.4 to − 177.6, *p* < 0.001) than minor (MD − 104.9 mL, 95% CI − 182.8 to − 27.1, *p* = 0.008; Figure S7B). LOS was significantly shorter in both subgroups (major: MD − 3.0 days, *p* < 0.001; minor: MD − 2.7 days, *p* = 0.013). Blood loss was also significantly reduced vs. laparoscopic in major hepatectomy (MD − 150.0 mL, *p* < 0.001; Figure S7E).

## Discussion

The aim of this network meta-analysis was to compare perioperative and oncologic outcomes of robot-assisted hepatectomy versus laparoscopic and open resection in adults undergoing curative-intent surgery for HCC, using the most recent comparative evidence. Across 23 studies including 9,666 patients, robotic hepatectomy was associated with consistently favorable perioperative outcomes, ranking first in 8 of 11 evaluated outcomes, and was associated with lower overall and major complication rates compared with open surgery, reduced blood loss, shorter hospital stay, and a lower conversion rate than laparoscopy, while maintaining comparable R0 resection rates.

These associations are consistent with the growing role of minimally invasive approaches in HCC surgery and are broadly consistent with the treatment-stage migration and expansion of minimally invasive resections endorsed in the 2024 EASL Clinical Practice Guidelines, including for more extensive anatomic resections [8].

An Earlier pairwise meta-analysis, Ziogas et al. (2021), including 525 patients, suggested broadly comparable perioperative and oncologic outcomes between laparoscopic and robotic minimally invasive hepatectomy. In that synthesis of retrospective cohorts, the two approaches did not differ meaningfully in overall or severe complications, mortality, conversion to open surgery, margin status, transfusion requirements, blood loss, operative time, or length of stay [4]. In contrast, our network meta-analysis integrating more recent, high-volume comparative data indicates a clearer separation in selected endpoints, most notably a lower conversion risk with robotic versus laparoscopic surgery. This divergence may reflect increasing team experience and standardization of robotic workflows over time, together with technical advantages of the robotic platform, such as wristed instrumentation, tremor filtration, and enhanced dexterity [19, 20].

Our subgroup analysis by hepatectomy extent revealed that the robotic advantage was most pronounced in major resections, with a significant reduction in major complications. Blood loss reduction was nearly double in major (MD − 200 mL) vs. minor (MD − 105 mL) resections, and Blood loss was also significantly reduced vs. laparoscopic in major hepatectomy. This suggests the robotic platform’s wristed instruments and 3D visualization provide maximum benefit during parenchymal transection near major hepatic veins [21]. However, only two studies comprised the predominantly major subgroup, and prospective studies specifically addressing major hepatectomies are warranted.

The association with lower major complications may reflect improved exposure and dissection. The robotic technique may mitigate the ‘fulcrum effect’ and 2-dimensional constraints, enabling fine dissection, especially in postero-superior segments (VII and VIII) [17, 22]. The association with reduced blood loss may reflect more precise transection. The release of next-generation robotic systems with force feedback, helping prevent excessive traction, tissue crushing, and suture breakage, is expected to further expand this safety margin [23].

Blood loss was significantly reduced with robotic hepatectomy (MD − 144.6 mL vs. open; MD − 51.2 mL vs. laparoscopic), consistent with Ziogas et al. (2021) [4]. Our subgroup analysis demonstrated a shorter length of stay after robotic surgery compared with other techniques, a finding that was consistent across both major and minor subgroups (Figure S7B).

The association with a lower conversion rate compared with laparoscopic surgery is potentially clinically meaningful, as conversion to open is independently associated with increased morbidity and costs. The enhanced dexterity of the robotic system may contribute to fewer conversions during dissection around major hepatic veins and in posterosuperior segments [21].

The cost of robotic surgery has traditionally been its Achilles’ heel [9, 23]. Although the present study did not analyze cost or economic outcomes directly, external evidence from recent published studies suggests a potential shift from cost-minimization to value-based evaluation. Studies by Rouault et al. (2025) and Millet et al. (2025) have reported that when incorporating the shorter length of stay, fewer conversions, and avoidance of expensive major complications (Clavien-Dindo grade > = III) into a ‘total cost of care’ analysis, robotic hepatectomy may approach cost-neutrality with open resection [10, 11, 24]. However, these cost findings were not generated by the present study, and formal health economic analyses with prospectively collected cost data are needed to validate these observations in diverse healthcare settings.

We found no significant differences in R0 resection margin or in 1-, 3-, and 5-year survival among the three modalities. This is consistent with the 2023 AASLD Practice Guidance [2]. The similarity of R0 rates is reassuring, implying that visually enhanced cues with ICG fluorescence have sufficiently filled the haptic gap [18, 25]. RLR appears oncologically non-inferior to open hepatectomy, consistent with Wang et al. (2025), who reported no statistically significant differences in disease-free or overall survival across the three groups [26].

### Limitations and future directions

Several limitations of this network meta-analysis should be considered when interpreting the results. First, all 23 included studies were retrospective observational cohorts, with no randomized controlled trials contributing to the evidence base. Although 73.9% of studies employed propensity score matching, this can only adjust for measured confounders and cannot eliminate residual confounding from unmeasured variables such as surgeon expertise, institutional learning curves, and patient selection algorithms. Patients selected for robotic hepatectomy may represent a more favorable cohort with respect to age, tumor biology, and hepatic reserve. Second, the direct evidence for the open versus laparoscopic comparison was derived solely from four three-arm studies, as no dedicated two-arm open versus laparoscopic studies were included; this may affect the precision of the indirect comparisons. Third, the subgroup analysis by extent of hepatectomy is exploratory and limited by the small number of contributing studies, which increases the risk of unstable estimates and spurious findings. Fourth, although R0 resection rates and the limited reported survival outcomes appeared comparable across approaches, these findings should be interpreted cautiously, as long-term oncologic outcomes were available from only a minority of studies, were not primary endpoints of the network meta-analysis, and could not be formally pooled in a time-to-event analysis because hazard ratios were insufficiently reported. Fifth, operative time demonstrated substantial heterogeneity (I-squared = 89.4%), likely reflecting variability in institutional experience, learning curves, and procedural complexity. Sixth, validated surgical complexity scores (e.g., Iwate Difficulty Score) were not consistently reported, precluding complexity-stratified analysis. Finally, the impact of emerging next-generation robotic platforms with force-feedback technology (e.g., da Vinci 5) has not been captured in the current evidence base, and future studies should incorporate these platforms alongside formal health economic assessments.

## Conclusion

This network meta-analysis, based entirely on retrospective observational evidence, suggests that robotic hepatectomy is associated with favorable perioperative outcomes compared with open and laparoscopic surgery for HCC, including lower complication rates, reduced blood loss, shorter hospital stay, and a lower conversion rate, while maintaining comparable oncological safety. However, given the absence of randomized evidence and the entirely retrospective nature of the included studies, these findings do not support definitive practice-changing recommendations at this time. Robotic hepatectomy may be considered a reasonable and potentially advantageous option for HCC treatment in selected patients at experienced, high-volume centers with established robotic programs. Further high-quality randomized controlled trials are needed to confirm these associations and establish the definitive role of robotic surgery in the HCC treatment algorithm.

## Supplementary Information

Below is the link to the electronic supplementary material.

Supplementary Material 1

Supplementary Material 2

Supplementary Material 3

Supplementary Material 4

## Data Availability

All data generated or analyzed during this study are included in this published article and its Supplementary Information.

## Abbreviations

AASLD: American Association for the Study of Liver Diseases
CI: Confidence interval
CINeMA: Confidence in Network Meta-Analysis
EASL: European Association for the Study of the Liver
GRADE: Grading of Recommendations Assessment Development and Evaluations
HCC: Hepatocellular carcinoma
ICG: Indocyanine green
LOS: Length of stay
MD: Mean difference
NMA: Network meta-analysis
OR: Odds ratio
OS: Overall survival
PHLF: Post-hepatectomy liver failure
PRISMA: Preferred Reporting Items for Systematic Reviews and Meta-Analyses
PSM: Propensity score matching
RCT: Randomized controlled trial
REML: Restricted maximum likelihood
RLR: Robotic liver resection
ROBINS-I: Risk of Bias in Non-Randomised Studies of Interventions

## Declarations

### PROSPERO Registration

CRD420251265604.
