# Supplementary material for: Robotic versus laparoscopic versus open hepatectomy for hepatocellular carcinoma: a systematic review and network meta-analysis
Source: J Robot Surg. 2026 Mar 30;20(1):380. doi: 10.1007/s11701-026-03344-2 (PMC13035557; doi:10.1007/s11701-026-03344-2)
Supplement: Supplementary file 1 — Supplementary Material 1 [file 11701_2026_3344_MOESM1_ESM.docx]

**Supplementary Table S10.** Transitivity Assessment: Distribution of Baseline Covariates Across Network Comparison Groups

*The transitivity assumption was assessed by comparing the distribution of key clinical and methodological covariates across the three direct comparison groups in the network. Values represent weighted means or pooled proportions from the robotic hepatectomy arms of each comparison group. No systematic imbalance was identified, supporting the plausibility of the transitivity assumption.*

| **Covariate** | **Rob vs Open (n = 7)** | **Rob vs Lap (n = 11)** | **Three-arm (n = 5)** |
| --- | --- | --- | --- |
| **Number of studies** | 7 | 11 | 5 |
| Total robotic patients, n | 852 | 1154 | 312 |
| Total control patients, n | 1272 | 5432 | 644 |
| Age (years), mean | 62.5 | 61.7 | 59.8 |
| Male sex (%) | 79.9 | 68.5 | 74.7 |
| BMI (kg/m²), mean | 25.7 | 25.8 | 24.5 |
| Cirrhosis (%) | 61.7 | 53.2 | 70.8 |
| Child-Pugh A (%) | 94.4 | 82.0 | 90.7 |
| HBV positive (%) | 72.8 | 25.6 | 47.4 |
| Tumor size (cm), mean | 4.2 | 3.3 | 3.4 |
| Multiple tumors (%) | 11.2 | 9.3 | 9.4 |
| AFP elevated (%) | 33.1 | 51.8 | 19.2 |

**Rob vs Open:** Zhang XP 2024, Zhang XP Elderly 2022, Pesi B 2021, Chen PD 2017, DiBenedetto F 2023, Nota CL 2019, Lin ZY 2023

**Rob vs Lap:** Li H 2024, Huang XK 2024, Giuliante F 2023, Balzano E 2022, Magistri P 2017, Bernardi L 2025, Duong LM 2022, Huang 2025 2025, Krenzien F 2024, DSilva M 2022, Montalti R 2016

**Three-arm:** Zhu P 2023, Kato Y 2023, Lim C 2021, Wang Y 2025, OConnell RM 2023

*Abbreviations: Rob, robotic; Lap, laparoscopic; BMI, body mass index; HBV, hepatitis B virus; AFP, alpha-fetoprotein; NR, not reported.
Values represent pooled data from the robotic hepatectomy arms within each comparison group. Three-arm studies contributed to all three pairwise comparisons within the network.*
