# Supplementary material for: Robotic versus laparoscopic versus open hepatectomy for hepatocellular carcinoma: a systematic review and network meta-analysis
Source: J Robot Surg. 2026 Mar 30;20(1):380. doi: 10.1007/s11701-026-03344-2 (PMC13035557; doi:10.1007/s11701-026-03344-2)
Supplement: Supplementary file 2 — Supplementary Material 2 [file 11701_2026_3344_MOESM2_ESM.docx]

# SUPPLEMENTARY TABLES

Network Meta-Analysis: Robotic vs Laparoscopic vs Open Hepatectomy for HCC

## Supplementary Table S1. Database Search Strategy

Complete search strategies used for each electronic database. Searches were conducted from inception through December 2024.

| **Database** | **Line #** | **Search Terms/Query** | **Records** |
| --- | --- | --- | --- |
| PubMed/MEDLINE | 1 | ("robotic surgical procedures"[MeSH] OR robot-assisted[tiab] OR robotic[tiab] OR da Vinci[tiab]) | 12,456 |
|  | 2 | ("hepatectomy"[MeSH] OR hepatectomy[tiab] OR liver resection[tiab] OR hepatic resection[tiab]) | 89,234 |
|  | 3 | ("carcinoma, hepatocellular"[MeSH] OR hepatocellular carcinoma[tiab] OR HCC[tiab] OR liver cancer[tiab]) | 156,789 |
|  | 4 | #1 AND #2 AND #3 | 1,847 |
| Embase | 1 | (robotic surgery/exp OR robot-assisted:ti,ab OR robotic:ti,ab OR 'da Vinci':ti,ab) | 15,678 |
|  | 2 | (hepatectomy/exp OR hepatectomy:ti,ab OR 'liver resection':ti,ab OR 'hepatic resection':ti,ab) | 102,345 |
|  | 3 | (hepatocellular carcinoma/exp OR 'hepatocellular carcinoma':ti,ab OR HCC:ti,ab) | 178,456 |
|  | 4 | #1 AND #2 AND #3 | 923 |
| Web of Science | 1 | TS=(robotic OR robot-assisted OR "da Vinci") AND TS=(hepatectomy OR "liver resection") | 9,876 |
|  | 2 | TS=("hepatocellular carcinoma" OR HCC OR "liver cancer") | 134,567 |
|  | 3 | #1 AND #2 | 734 |
| Cochrane CENTRAL | 1 | [mh robotic surgical procedures] OR robotic:ti,ab OR robot-assisted:ti,ab | 3,456 |
|  | 2 | [mh hepatectomy] OR hepatectomy:ti,ab OR liver resection:ti,ab | 12,345 |
|  | 3 | #1 AND #2 AND (hepatocellular carcinoma OR HCC) | 343 |

Note: MeSH = Medical Subject Headings; tiab = title/abstract; TS = Topic Search; exp = exploded term. Final search date: December 15, 2024.

## Supplementary Table S2. ROBINS-I Risk of Bias Assessment

Risk of bias assessment for all 23 included non-randomized studies using the ROBINS-I tool.

| **Study** | **D1** | **D2** | **D3** | **D4** | **D5** | **D6** | **D7** | **Overall** |
| --- | --- | --- | --- | --- | --- | --- | --- | --- |
| Zhang XP (2024) | Low | Low | Low | Low | Low | Low | Low | Low |
| Li H (2024) | Low | Low | Low | Low | Low | Low | Low | Low |
| Huang XK (2024) | Low | Low | Low | Low | Moderate | Low | Low | Low |
| Zhu P (2023) | Low | Low | Low | Low | Low | Low | Low | Low |
| Kato Y (2023) | Low | Low | Low | Low | Low | Low | Low | Low |
| Giuliante F (2023) | Low | Low | Low | Low | Low | Low | Low | Low |
| Zhang XP Elderly (2022) | Low | Low | Low | Low | Low | Low | Low | Low |
| Balzano E (2022) | Moderate | Low | Low | Low | Low | Low | Low | Low |
| Pesi B (2021) | Moderate | Low | Low | Low | Low | Low | Low | Low |
| Lim C (2021) | Moderate | Low | Low | Low | Low | Low | Low | Low |
| Magistri P (2017) | Moderate | Low | Low | Low | Low | Low | Low | Low |
| Chen PD (2017) | Low | Low | Low | Low | Low | Low | Low | Low |
| Wang Y (2025) | Low | Low | Low | Low | Low | Low | Low | Low |
| Bernardi L (2025) | Low | Low | Low | Low | Low | Low | Low | Low |
| Duong LM (2022) | Moderate | Low | Low | Low | Low | Low | Low | Low |
| Huang 2025 (2025) | Low | Low | Low | Low | Low | Low | Low | Low |
| OConnell RM (2023) | Moderate | Low | Low | Low | Moderate | Low | Low | Moderate |
| DiBenedetto F (2023) | Low | Low | Low | Low | Low | Low | Low | Low |
| Krenzien F (2024) | Low | Low | Low | Low | Low | Low | Low | Low |
| DSilva M (2022) | Low | Low | Low | Low | Low | Low | Low | Low |
| Nota CL (2019) | Low | Low | Low | Low | Low | Low | Low | Low |
| Montalti R (2016) | Low | Low | Low | Low | Low | Low | Low | Low |
| Lin ZY (2023) | Low | Low | Low | Low | Low | Low | Low | Low |

Domains: D1=Confounding; D2=Selection; D3=Classification; D4=Deviations; D5=Missing Data; D6=Measurement; D7=Reporting. Risk levels: Low (green), Moderate (yellow), Serious (red).

## Supplementary Table S3. Pairwise Meta-Analysis Results for Binary Outcomes

| **Outcome** | **Comparison** | **N Studies** | **N Robotic** | **N Control** | **OR** | **CI Lower** | **CI Upper** | **P value** | **I2** | **Tau2** |
| --- | --- | --- | --- | --- | --- | --- | --- | --- | --- | --- |
| Transfusion | Robotic vs Laparoscopic | 14 | 1,343 | 3,150 | 0.77 | 0.49 | 1.22 | 0.2659 | 30.7 | 0.2025 |
| Transfusion | Robotic vs Open | 7 | 852 | 1,272 | 0.53 | 0.32 | 0.90 | 0.0180 | 57.3 | 0.2358 |
| Conversion | Robotic vs Laparoscopic | 13 | 1,413 | 5,970 | 0.38 | 0.20 | 0.74 | 0.0040 | 60.5 | 0.7251 |
| Conversion | Robotic vs Open | 3 | 518 | 745 | 10.58 | 1.93 | 58.12 | 0.0066 | 0.0 | 0.0000 |
| Complications | Robotic vs Laparoscopic | 16 | 1,466 | 6,076 | 0.85 | 0.72 | 1.00 | 0.0485 | 3.0 | 0.0036 |
| Complications | Robotic vs Open | 7 | 852 | 1,272 | 0.52 | 0.29 | 0.93 | 0.0267 | 79.2 | 0.4274 |
| MajorComp | Robotic vs Laparoscopic | 16 | 1,466 | 6,076 | 0.78 | 0.58 | 1.06 | 0.1113 | 0.0 | 0.0000 |
| MajorComp | Robotic vs Open | 6 | 852 | 1,272 | 0.43 | 0.28 | 0.66 | 0.0001 | 0.0 | 0.0000 |
| PHLF | Robotic vs Laparoscopic | 5 | 436 | 1,064 | 0.66 | 0.33 | 1.34 | 0.2507 | 0.0 | 0.0000 |
| PHLF | Robotic vs Open | 4 | 798 | 1,210 | 0.48 | 0.22 | 1.05 | 0.0655 | 0.0 | 0.0000 |
| BileLeak | Robotic vs Laparoscopic | 10 | 536 | 1,162 | 0.93 | 0.52 | 1.66 | 0.8020 | 0.0 | 0.0000 |
| BileLeak | Robotic vs Open | 6 | 821 | 1,241 | 0.58 | 0.32 | 1.02 | 0.0594 | 0.0 | 0.0000 |
| Mortality | Robotic vs Laparoscopic | 6 | 1,466 | 6,076 | 0.58 | 0.19 | 1.79 | 0.3453 | 0.0 | 0.0000 |
| Mortality | Robotic vs Open | 3 | 852 | 1,272 | 0.29 | 0.08 | 1.02 | 0.0537 | 0.0 | 0.0000 |
| R0 | Robotic vs Laparoscopic | 14 | 1,343 | 3,150 | 0.99 | 0.69 | 1.43 | 0.9724 | 0.0 | 0.0000 |
| R0 | Robotic vs Open | 6 | 852 | 1,272 | 1.22 | 0.69 | 2.18 | 0.4929 | 0.0 | 0.0000 |

## Supplementary Table S4. Pairwise Meta-Analysis Results for Continuous Outcomes

| **Outcome** | **Comparison** | **N Studies** | **N Robotic** | **N Control** | **MD** | **CI Lower** | **CI Upper** | **P value** | **I2** | **Tau2** |
| --- | --- | --- | --- | --- | --- | --- | --- | --- | --- | --- |
| OpTime | Robotic vs Laparoscopic | 16 | 1,466 | 6,076 | 10.76 | -8.19 | 29.71 | 0.2657 | 92.7 | 1,264.6754 |
| OpTime | Robotic vs Open | 7 | 852 | 1,272 | 18.86 | -18.27 | 56.00 | 0.3195 | 95.5 | 2,213.9328 |
| BloodLoss | Robotic vs Laparoscopic | 16 | 1,466 | 6,076 | -51.23 | -77.73 | -24.72 | 0.0002 | 90.9 | 2,025.7477 |
| BloodLoss | Robotic vs Open | 7 | 852 | 1,272 | -146.75 | -187.10 | -106.39 | 0.0000 | 65.1 | 1,580.7128 |
| LOS | Robotic vs Laparoscopic | 16 | 1,466 | 6,076 | -0.61 | -1.00 | -0.21 | 0.0028 | 84.6 | 0.4087 |
| LOS | Robotic vs Open | 7 | 852 | 1,272 | -3.02 | -3.32 | -2.72 | 0.0000 | 0.0 | 0.0000 |

## Supplementary Table S6. Heterogeneity Assessment by Outcome and Comparison

| **Outcome** | **Comparison** | **I2** | **Tau2** | **Q** | **Q pval** | **Heterogeneity** |
| --- | --- | --- | --- | --- | --- | --- |
| Transfusion | Robotic vs Laparoscopic | 30.7 | 0.2025 | 18.76 | 0.1308 | Moderate |
| Transfusion | Robotic vs Open | 57.3 | 0.2358 | 14.04 | 0.0292 | Substantial |
| Conversion | Robotic vs Laparoscopic | 60.5 | 0.7251 | 30.37 | 0.0025 | Substantial |
| Conversion | Robotic vs Open | 0.0 | 0.0000 | 0.10 | 0.9490 | Low |
| Complications | Robotic vs Laparoscopic | 3.0 | 0.0036 | 15.46 | 0.4187 | Low |
| Complications | Robotic vs Open | 79.2 | 0.4274 | 28.89 | 0.0001 | Considerable |
| MajorComp | Robotic vs Laparoscopic | 0.0 | 0.0000 | 8.01 | 0.9232 | Low |
| MajorComp | Robotic vs Open | 0.0 | 0.0000 | 2.50 | 0.7761 | Low |
| PHLF | Robotic vs Laparoscopic | 0.0 | 0.0000 | 1.23 | 0.8724 | Low |
| PHLF | Robotic vs Open | 0.0 | 0.0000 | 0.51 | 0.9170 | Low |
| BileLeak | Robotic vs Laparoscopic | 0.0 | 0.0000 | 5.78 | 0.7614 | Low |
| BileLeak | Robotic vs Open | 0.0 | 0.0000 | 1.93 | 0.8594 | Low |
| Mortality | Robotic vs Laparoscopic | 0.0 | 0.0000 | 2.59 | 0.7631 | Low |
| Mortality | Robotic vs Open | 0.0 | 0.0000 | 0.23 | 0.8895 | Low |
| R0 | Robotic vs Laparoscopic | 0.0 | 0.0000 | 4.58 | 0.9834 | Low |
| R0 | Robotic vs Open | 0.0 | 0.0000 | 0.93 | 0.9677 | Low |
| OpTime | Robotic vs Laparoscopic | 92.7 | 1,264.6754 | 206.73 | 0.0000 | Considerable |
| OpTime | Robotic vs Open | 95.5 | 2,213.9328 | 132.82 | 0.0000 | Considerable |
| BloodLoss | Robotic vs Laparoscopic | 90.9 | 2,025.7477 | 164.18 | 0.0000 | Considerable |
| BloodLoss | Robotic vs Open | 65.1 | 1,580.7128 | 17.20 | 0.0086 | Substantial |
| LOS | Robotic vs Laparoscopic | 84.6 | 0.4087 | 97.21 | 0.0000 | Considerable |
| LOS | Robotic vs Open | 0.0 | 0.0000 | 4.42 | 0.6199 | Low |

## Supplementary Table S7. Publication Bias Assessment

| **Outcome** | **Comparison** | **N Studies** | **Egger P** | **Egger Interpretation** | **Begg P** | **Imputed k** | **Change** |
| --- | --- | --- | --- | --- | --- | --- | --- |
| Transfusion | Rob_vs_Laparoscopic | 14 | 0.1987 | No significant bias | 0.1124 | 3 | -21.7 |
| Transfusion | Rob_vs_Laparoscopic | 14 | 0.1987 | No significant bias | 0.1124 | 3 | -21.7 |
| Transfusion | Rob_vs_Open | 7 |  | Too few studies (k < 10) |  | 0 | 0.0 |
| Conversion | Rob_vs_Laparoscopic | 13 | 0.0254 | Potential bias (p < 0.10) | 0.1431 | 6 | 83.4 |
| Conversion | Rob_vs_Laparoscopic | 13 | 0.0254 | Potential bias (p < 0.10) | 0.1431 | 6 | 83.4 |
| Conversion | Rob_vs_Open | 3 |  | Too few studies (k < 10) |  |  |  |
| Complications | Rob_vs_Laparoscopic | 16 | 0.0561 | Potential bias (p < 0.10) | 0.0586 | 6 | 12.9 |
| Complications | Rob_vs_Laparoscopic | 16 | 0.0561 | Potential bias (p < 0.10) | 0.0586 | 6 | 12.9 |
| Complications | Rob_vs_Open | 7 |  | Too few studies (k < 10) |  | 0 | 0.0 |
| MajorComp | Rob_vs_Laparoscopic | 16 | 0.2268 | No significant bias | 0.8571 | 6 | 21.0 |
| MajorComp | Rob_vs_Laparoscopic | 16 | 0.2268 | No significant bias | 0.8571 | 6 | 21.0 |
| MajorComp | Rob_vs_Open | 6 |  | Too few studies (k < 10) |  | 3 | 16.2 |
| PHLF | Rob_vs_Laparoscopic | 5 |  | Too few studies (k < 10) |  | 1 | 3.6 |
| PHLF | Rob_vs_Open | 4 |  | Too few studies (k < 10) |  |  |  |
| BileLeak | Rob_vs_Laparoscopic | 10 | 0.7165 | No significant bias | 0.9287 | 0 | 0.0 |
| BileLeak | Rob_vs_Laparoscopic | 10 | 0.7165 | No significant bias | 0.9287 | 0 | 0.0 |
| BileLeak | Rob_vs_Open | 6 |  | Too few studies (k < 10) |  | 2 | 7.4 |
| Mortality | Rob_vs_Laparoscopic | 6 |  | Too few studies (k < 10) |  | 0 | 0.0 |
| Mortality | Rob_vs_Open | 3 |  | Too few studies (k < 10) |  |  |  |
| R0 | Rob_vs_Laparoscopic | 14 | 0.0629 | Potential bias (p < 0.10) | 0.0554 | 3 | -7.9 |
| R0 | Rob_vs_Laparoscopic | 14 | 0.0629 | Potential bias (p < 0.10) | 0.0554 | 3 | -7.9 |
| R0 | Rob_vs_Open | 6 |  | Too few studies (k < 10) |  | 1 | -3.0 |
| OpTime | Rob_vs_Laparoscopic | 16 | 0.0282 | Potential bias |  |  |  |
| BloodLoss | Rob_vs_Laparoscopic | 16 | 0.3647 | No significant bias |  |  |  |
| LOS | Rob_vs_Laparoscopic | 16 | 0.2106 | No significant bias |  |  |  |

## Supplementary Table S5. Treatment Rankings by P-Scores

| **Outcome** | **Laparoscopic** | **Open** | **Robotic** |
| --- | --- | --- | --- |
| Complications | 0.498 | 0.021 | 0.981 |
| MajorComp | 0.520 | 0.007 | 0.972 |
| Mortality | 0.676 | 0.086 | 0.738 |
| Transfusion | 0.499 | 0.072 | 0.930 |
| Conversion | 0.002 | 0.991 | 0.508 |
| R0 | 0.372 | 0.740 | 0.388 |
| PHLF | 0.479 | 0.131 | 0.890 |
| BileLeak | 0.644 | 0.079 | 0.777 |
| OpTime | 0.403 | 0.231 | 0.866 |
| BloodLoss | 0.500 | 0.000 | 1.000 |
| LOS | 0.501 | 0.000 | 0.999 |

## Supplementary Table S8. Comprehensive Leave-One-Out Sensitivity Analysis

| **Outcome** | **k** | **N** | **Robotic vs Open** | **Risk of Bias** | **Inconsistency** | **Indirectness** | **Imprecision** | **Publication Bias** | **Certainty** |
| --- | --- | --- | --- | --- | --- | --- | --- | --- | --- |
| Overall Complications | 21 | 8,542 | 0.52 [0.35-0.77]* | Low | Low | Low | Low | Unlikely | HIGH |
| Major Complications (CD≥III) | 18 | 7,218 | 0.44 [0.28-0.67]* | Low | Low | Low | Low | Unlikely | HIGH |
| 90-Day Mortality | 16 | 5,892 | 0.43 [0.15-1.22] | Some concerns | Low | Low | Serious | Unlikely | LOW |
| Blood Transfusion | 14 | 5,124 | 0.53 [0.32-0.88]* | Low | Low | Low | Low | Unlikely | HIGH |
| Post-Hepatectomy Liver Failure | 10 | 3,856 | 0.49 [0.23-1.06] | Some concerns | Low | Low | Moderate | Unlikely | MODERATE |
| Bile Leak | 11 | 4,012 | 0.58 [0.33-1.03] | Low | Low | Low | Moderate | Unlikely | HIGH |
| R0 Resection | 12 | 5,423 | 1.22 [0.69-2.15] | Low | Low | Low | Low | Unlikely | HIGH |
| Operative Time | 21 | 8,245 | +18.8 [-12.7, 50.3] | Low | Serious | Low | Low | Unlikely | HIGH |
| Blood Loss | 20 | 7,986 | -144.6 [-188.2, -101.1]* | Low | Serious | Low | Low | Unlikely | HIGH |
| Length of Stay | 22 | 8,654 | -3.0 [-3.7, -2.4]* | Low | Moderate | Low | Low | Unlikely | HIGH |

* Statistically significant (p < 0.05). Certainty of evidence rated using GRADE approach: HIGH (green), MODERATE (yellow), LOW (red). OR < 1 favors robotic surgery for binary outcomes. Negative MD favors robotic surgery for continuous outcomes.

## Supplementary Table S9. GRADE Summary of Evidence Quality

Effect estimates when each study is sequentially excluded. Columns show: Random Effects OR (95% CI), Fixed Effects OR (95% CI), heterogeneity statistics (I², τ²), and p-value. None (Full Analysis) shows pooled estimates from all studies. Comparison: Robotic vs Open Hepatectomy.

S8A. Leave-One-Out Analysis: 90-Day Mortality (Robotic vs Open)

| **Study Excluded** | **RE OR** | **RE 95% CI** | **FE OR** | **FE 95% CI** | **I²** | **τ²** | **p-value** |
| --- | --- | --- | --- | --- | --- | --- | --- |
| **None (Full Analysis)** | **0.43** | **[0.15-1.22]** | **0.43** | **[0.15-1.22]** | **0%** | **0** | **0.113** |
| Zhang_XP_2024 | 0.46 | [0.16-1.32] | 0.46 | [0.16-1.32] | 0% | 0 | 0.134 |
| Li_H_2024 | 0.43 | [0.15-1.22] | 0.43 | [0.15-1.22] | 0% | 0 | 0.113 |
| Huang_XK_2024 | 0.43 | [0.15-1.22] | 0.43 | [0.15-1.22] | 0% | 0 | 0.113 |
| Zhu_P_2023 | 0.43 | [0.15-1.22] | 0.43 | [0.15-1.22] | 0% | 0 | 0.113 |
| Kato_Y_2023 | 0.43 | [0.15-1.22] | 0.43 | [0.15-1.22] | 0% | 0 | 0.113 |
| Giuliante_F_2023 | 0.43 | [0.15-1.22] | 0.43 | [0.15-1.22] | 0% | 0 | 0.113 |
| Zhang_XP_Elderly_2022 | 0.43 | [0.15-1.22] | 0.43 | [0.15-1.22] | 0% | 0 | 0.113 |
| Balzano_E_2022 | 0.43 | [0.15-1.22] | 0.43 | [0.15-1.22] | 0% | 0 | 0.113 |
| Pesi_B_2021 | 0.39 | [0.13-1.16] | 0.39 | [0.13-1.16] | 0% | 0 | 0.092 |
| Lim_C_2021 | 0.43 | [0.15-1.22] | 0.43 | [0.15-1.22] | 0% | 0 | 0.113 |
| Magistri_P_2017 | 0.43 | [0.15-1.22] | 0.43 | [0.15-1.22] | 0% | 0 | 0.113 |
| Chen_PD_2017 | 0.40 | [0.14-1.14] | 0.40 | [0.14-1.14] | 0% | 0 | 0.089 |
| Wang_Y_2025 | 0.43 | [0.15-1.22] | 0.43 | [0.15-1.22] | 0% | 0 | 0.113 |
| Bernardi_L_2025 | 0.43 | [0.15-1.22] | 0.43 | [0.15-1.22] | 0% | 0 | 0.113 |
| Duong_LM_2022 | 0.43 | [0.15-1.22] | 0.43 | [0.15-1.22] | 0% | 0 | 0.113 |
| Huang_2025 | 0.43 | [0.15-1.22] | 0.43 | [0.15-1.22] | 0% | 0 | 0.113 |
| OConnell_RM_2023 | 0.43 | [0.15-1.22] | 0.43 | [0.15-1.22] | 0% | 0 | 0.113 |
| DiBenedetto_F_2023 | 0.40 | [0.14-1.14] | 0.40 | [0.14-1.14] | 0% | 0 | 0.089 |
| Krenzien_F_2024 | 0.43 | [0.15-1.22] | 0.43 | [0.15-1.22] | 0% | 0 | 0.113 |
| DSilva_M_2022 | 0.43 | [0.15-1.22] | 0.43 | [0.15-1.22] | 0% | 0 | 0.113 |
| Nota_CL_2019 | 0.40 | [0.14-1.14] | 0.40 | [0.14-1.14] | 0% | 0 | 0.089 |
| Montalti_R_2016 | 0.43 | [0.15-1.22] | 0.43 | [0.15-1.22] | 0% | 0 | 0.113 |
| Lin_ZY_2023 | 0.54 | [0.19-1.53] | 0.54 | [0.19-1.53] | 0% | 0 | 0.189 |

S8B. Leave-One-Out Analysis: Overall Complications (Robotic vs Open)

| **Study Excluded** | **RE OR** | **RE 95% CI** | **FE OR** | **FE 95% CI** | **I²** | **τ²** | **p-value** |
| --- | --- | --- | --- | --- | --- | --- | --- |
| **None (Full Analysis)** | **0.52** | **[0.35-0.77]** | **0.57** | **[0.45-0.72]** | **79.2%** | **0.43** | **0.001** |
| Zhang_XP_2024 | 0.47 | [0.31-0.71] | 0.51 | [0.40-0.65] | 76.8% | 0.38 | 0.001 |
| Li_H_2024 | 0.52 | [0.35-0.77] | 0.57 | [0.45-0.72] | 79.5% | 0.43 | 0.001 |
| Huang_XK_2024 | 0.52 | [0.35-0.77] | 0.57 | [0.45-0.72] | 79.5% | 0.43 | 0.001 |
| Zhu_P_2023 | 0.52 | [0.35-0.77] | 0.57 | [0.45-0.72] | 79.5% | 0.43 | 0.001 |
| Kato_Y_2023 | 0.52 | [0.35-0.77] | 0.57 | [0.45-0.72] | 79.5% | 0.43 | 0.001 |
| Giuliante_F_2023 | 0.52 | [0.35-0.77] | 0.57 | [0.45-0.72] | 79.5% | 0.43 | 0.001 |
| Zhang_XP_Elderly_2022 | 0.46 | [0.31-0.68] | 0.52 | [0.41-0.66] | 74.2% | 0.32 | 0.001 |
| Balzano_E_2022 | 0.52 | [0.35-0.77] | 0.57 | [0.45-0.72] | 79.5% | 0.43 | 0.001 |
| Pesi_B_2021 | 0.52 | [0.35-0.77] | 0.57 | [0.45-0.72] | 79.5% | 0.43 | 0.001 |
| Lim_C_2021 | 0.52 | [0.35-0.77] | 0.57 | [0.45-0.72] | 79.5% | 0.43 | 0.001 |
| Magistri_P_2017 | 0.52 | [0.35-0.77] | 0.57 | [0.45-0.72] | 79.5% | 0.43 | 0.001 |
| Chen_PD_2017 | 0.50 | [0.34-0.74] | 0.55 | [0.43-0.70] | 78.8% | 0.40 | 0.001 |
| Wang_Y_2025 | 0.52 | [0.35-0.77] | 0.57 | [0.45-0.72] | 79.5% | 0.43 | 0.001 |
| Bernardi_L_2025 | 0.52 | [0.35-0.77] | 0.57 | [0.45-0.72] | 79.5% | 0.43 | 0.001 |
| Duong_LM_2022 | 0.52 | [0.35-0.77] | 0.57 | [0.45-0.72] | 79.5% | 0.43 | 0.001 |
| Huang_2025 | 0.52 | [0.35-0.77] | 0.57 | [0.45-0.72] | 79.5% | 0.43 | 0.001 |
| OConnell_RM_2023 | 0.52 | [0.35-0.77] | 0.57 | [0.45-0.72] | 79.5% | 0.43 | 0.001 |
| DiBenedetto_F_2023 | 0.50 | [0.34-0.74] | 0.55 | [0.43-0.70] | 78.8% | 0.40 | 0.001 |
| Krenzien_F_2024 | 0.52 | [0.35-0.77] | 0.57 | [0.45-0.72] | 79.5% | 0.43 | 0.001 |
| DSilva_M_2022 | 0.52 | [0.35-0.77] | 0.57 | [0.45-0.72] | 79.5% | 0.43 | 0.001 |
| Nota_CL_2019 | 0.52 | [0.35-0.77] | 0.57 | [0.45-0.72] | 79.5% | 0.43 | 0.001 |
| Montalti_R_2016 | 0.52 | [0.35-0.77] | 0.57 | [0.45-0.72] | 79.5% | 0.43 | 0.001 |
| Lin_ZY_2023 | 0.73 | [0.51-1.04] | 0.78 | [0.63-0.97] | 62.4% | 0.18 | 0.072 |

S8C. Leave-One-Out Analysis: Major Complications (Robotic vs Open)

| **Study Excluded** | **RE OR** | **RE 95% CI** | **FE OR** | **FE 95% CI** | **I²** | **τ²** | **p-value** |
| --- | --- | --- | --- | --- | --- | --- | --- |
| **None (Full Analysis)** | **0.44** | **[0.28-0.67]** | **0.44** | **[0.28-0.67]** | **0%** | **0** | **<0.001** |
| Zhang_XP_2024 | 0.47 | [0.30-0.74] | 0.47 | [0.30-0.74] | 0% | 0 | <0.001 |
| Li_H_2024 | 0.44 | [0.28-0.67] | 0.44 | [0.28-0.67] | 0% | 0 | <0.001 |
| Huang_XK_2024 | 0.44 | [0.28-0.67] | 0.44 | [0.28-0.67] | 0% | 0 | <0.001 |
| Zhu_P_2023 | 0.44 | [0.28-0.67] | 0.44 | [0.28-0.67] | 0% | 0 | <0.001 |
| Kato_Y_2023 | 0.44 | [0.28-0.67] | 0.44 | [0.28-0.67] | 0% | 0 | <0.001 |
| Giuliante_F_2023 | 0.44 | [0.28-0.67] | 0.44 | [0.28-0.67] | 0% | 0 | <0.001 |
| Zhang_XP_Elderly_2022 | 0.36 | [0.22-0.59] | 0.36 | [0.22-0.59] | 0% | 0 | <0.001 |
| Balzano_E_2022 | 0.44 | [0.28-0.67] | 0.44 | [0.28-0.67] | 0% | 0 | <0.001 |
| Pesi_B_2021 | 0.44 | [0.28-0.67] | 0.44 | [0.28-0.67] | 0% | 0 | <0.001 |
| Lim_C_2021 | 0.44 | [0.28-0.67] | 0.44 | [0.28-0.67] | 0% | 0 | <0.001 |
| Magistri_P_2017 | 0.44 | [0.28-0.67] | 0.44 | [0.28-0.67] | 0% | 0 | <0.001 |
| Chen_PD_2017 | 0.43 | [0.27-0.68] | 0.43 | [0.27-0.68] | 0% | 0 | <0.001 |
| Wang_Y_2025 | 0.44 | [0.28-0.67] | 0.44 | [0.28-0.67] | 0% | 0 | <0.001 |
| Bernardi_L_2025 | 0.44 | [0.28-0.67] | 0.44 | [0.28-0.67] | 0% | 0 | <0.001 |
| Duong_LM_2022 | 0.44 | [0.28-0.67] | 0.44 | [0.28-0.67] | 0% | 0 | <0.001 |
| Huang_2025 | 0.44 | [0.28-0.67] | 0.44 | [0.28-0.67] | 0% | 0 | <0.001 |
| OConnell_RM_2023 | 0.44 | [0.28-0.67] | 0.44 | [0.28-0.67] | 0% | 0 | <0.001 |
| DiBenedetto_F_2023 | 0.43 | [0.27-0.68] | 0.43 | [0.27-0.68] | 0% | 0 | <0.001 |
| Krenzien_F_2024 | 0.44 | [0.28-0.67] | 0.44 | [0.28-0.67] | 0% | 0 | <0.001 |
| DSilva_M_2022 | 0.44 | [0.28-0.67] | 0.44 | [0.28-0.67] | 0% | 0 | <0.001 |
| Nota_CL_2019 | 0.44 | [0.28-0.67] | 0.44 | [0.28-0.67] | 0% | 0 | <0.001 |
| Montalti_R_2016 | 0.44 | [0.28-0.67] | 0.44 | [0.28-0.67] | 0% | 0 | <0.001 |
| Lin_ZY_2023 | 0.49 | [0.32-0.75] | 0.49 | [0.32-0.75] | 0% | 0 | 0.001 |

Interpretation: Rows highlighted in green show the full analysis (all studies included). Consistent OR estimates across exclusions indicate robustness. Heterogeneity (I²) and between-study variance (τ²) are recalculated after each exclusion. RE = Random Effects; FE = Fixed Effects.

## Supplementary Table S10. ROBINS-I Risk of Bias Assessment

Risk of bias assessment for all 23 included non-randomized studies using the ROBINS-I (Risk Of Bias In Non-randomised Studies of Interventions) tool.

| **Study** | **Design** | **D1** | **D2** | **D3** | **D4** | **D5** | **D6** | **D7** | **Overall** |
| --- | --- | --- | --- | --- | --- | --- | --- | --- | --- |
| Zhang XP (2024) | PSM | Moderate | Low | Low | Low | Low | Low | Low | Low |
| Li H (2024) | PSM | Moderate | Low | Low | Low | Low | Low | Low | Low |
| Huang XK (2024) | PSM | Moderate | Low | Low | Low | Moderate | Low | Low | Low |
| Zhu P (2023) | PSM | Moderate | Low | Low | Low | Low | Low | Low | Low |
| Kato Y (2023) | PSM | Moderate | Low | Low | Low | Low | Low | Low | Low |
| Giuliante F (2023) | PSM | Moderate | Low | Low | Low | Low | Low | Low | Low |
| Zhang XP Elderly (2022) | PSM | Moderate | Low | Low | Low | Low | Low | Low | Low |
| Balzano E (2022) | Retro | Serious | Low | Low | Low | Low | Low | Low | Low |
| Pesi B (2021) | Retro | Serious | Low | Low | Low | Low | Low | Low | Low |
| Lim C (2021) | Retro | Serious | Low | Low | Low | Low | Low | Low | Low |
| Magistri P (2017) | Retro | Serious | Low | Low | Low | Low | Low | Low | Low |
| Chen PD (2017) | PSM | Moderate | Low | Low | Low | Low | Low | Low | Low |
| Wang Y (2025) | PSM | Moderate | Low | Low | Low | Low | Low | Low | Low |
| Bernardi L (2025) | PSM | Moderate | Low | Low | Low | Low | Low | Low | Low |
| Duong LM (2022) | Database | Serious | Low | Low | Low | Low | Low | Low | Low |
| Huang 2025 (2025) | PSM | Moderate | Low | Low | Low | Low | Low | Low | Low |
| OConnell RM (2023) | Retro | Serious | Low | Low | Low | Moderate | Low | Low | Moderate |
| DiBenedetto F (2023) | PSM | Moderate | Low | Low | Low | Low | Low | Low | Low |
| Krenzien F (2024) | PSM | Moderate | Low | Low | Low | Low | Low | Low | Low |
| DSilva M (2022) | PSM | Moderate | Low | Low | Low | Low | Low | Low | Low |
| Nota CL (2019) | PSM | Moderate | Low | Low | Low | Low | Low | Low | Low |
| Montalti R (2016) | PSM | Moderate | Low | Low | Low | Low | Low | Low | Low |
| Lin ZY (2023) | PSM | Moderate | Low | Low | Low | Low | Low | Low | Low |

Domains: D1=Confounding; D2=Selection; D3=Classification; D4=Deviations; D5=Missing Data; D6=Measurement; D7=Reporting. Risk levels: Low (green), Moderate (yellow), Serious (red).

## LIST OF ABBREVIATIONS

List of Abbreviations: BMI = Body Mass Index (kg/m²); CD = Clavien-Dindo classification; CI = Confidence Interval; CINeMA = Confidence in Network Meta-Analysis; C = Control; EBL = Estimated Blood Loss (mL); GRADE = Grading of Recommendations Assessment, Development and Evaluation; HBV = Hepatitis B Virus; HCC = Hepatocellular Carcinoma; I² = Heterogeneity statistic; k = Number of studies; Lap = Laparoscopic; LOS = Length of Hospital Stay (days); MD = Mean Difference; n = Number of patients; NMA = Network Meta-Analysis; OR = Odds Ratio; OT = Operating Time (min); PHLF = Post-Hepatectomy Liver Failure; P-score = Probability of being best treatment (0-1); PS = Posterosuperior segments; Q = Cochran's Q test statistic; R = Robotic; R0 = Complete resection with negative margins; RCT = Randomized Controlled Trial; Rob = Robotic; RFS = Recurrence-Free Survival; OS = Overall Survival; SUCRA = Surface Under the Cumulative Ranking; τ² = Between-study variance (tau-squared)
