# Supplementary material for: Robotic versus laparoscopic versus open hepatectomy for hepatocellular carcinoma: a systematic review and network meta-analysis
Source: J Robot Surg. 2026 Mar 30;20(1):380. doi: 10.1007/s11701-026-03344-2 (PMC13035557; doi:10.1007/s11701-026-03344-2)
Supplement: Supplementary file 3 — Supplementary Material 3 [file 11701_2026_3344_MOESM3_ESM.docx]

# SUPPLEMENTARY FIGURES

Network Meta-Analysis: Robotic versus Laparoscopic versus Open Hepatectomy for Hepatocellular Carcinoma - A Comprehensive Comparative Effectiveness Analysis

### Contents:

Supplementary Figure S1. Treatment Ranking Heatmap

Supplementary Figure S2. P-Score Bar Chart for Treatment Rankings

Supplementary Figure S3. Rankograms (Cumulative Ranking Probability)

Supplementary Figure S4. Node-Splitting Analysis for Inconsistency Assessment

Supplementary Figure S5. Funnel Plots for Publication Bias Assessment

Supplementary Figure S6. Long-Term Survival Outcomes (A-F)

Supplementary Figure S7. Subgroup Analysis Forest Plots (A–K)

## SUPPLEMENTARY FIGURE S1

### Treatment Ranking Heatmap

Heatmap showing P-score rankings for each treatment across all outcomes. Darker colors indicate better performance (higher P-score). Robotic hepatectomy shows favorable rankings for most perioperative outcomes.


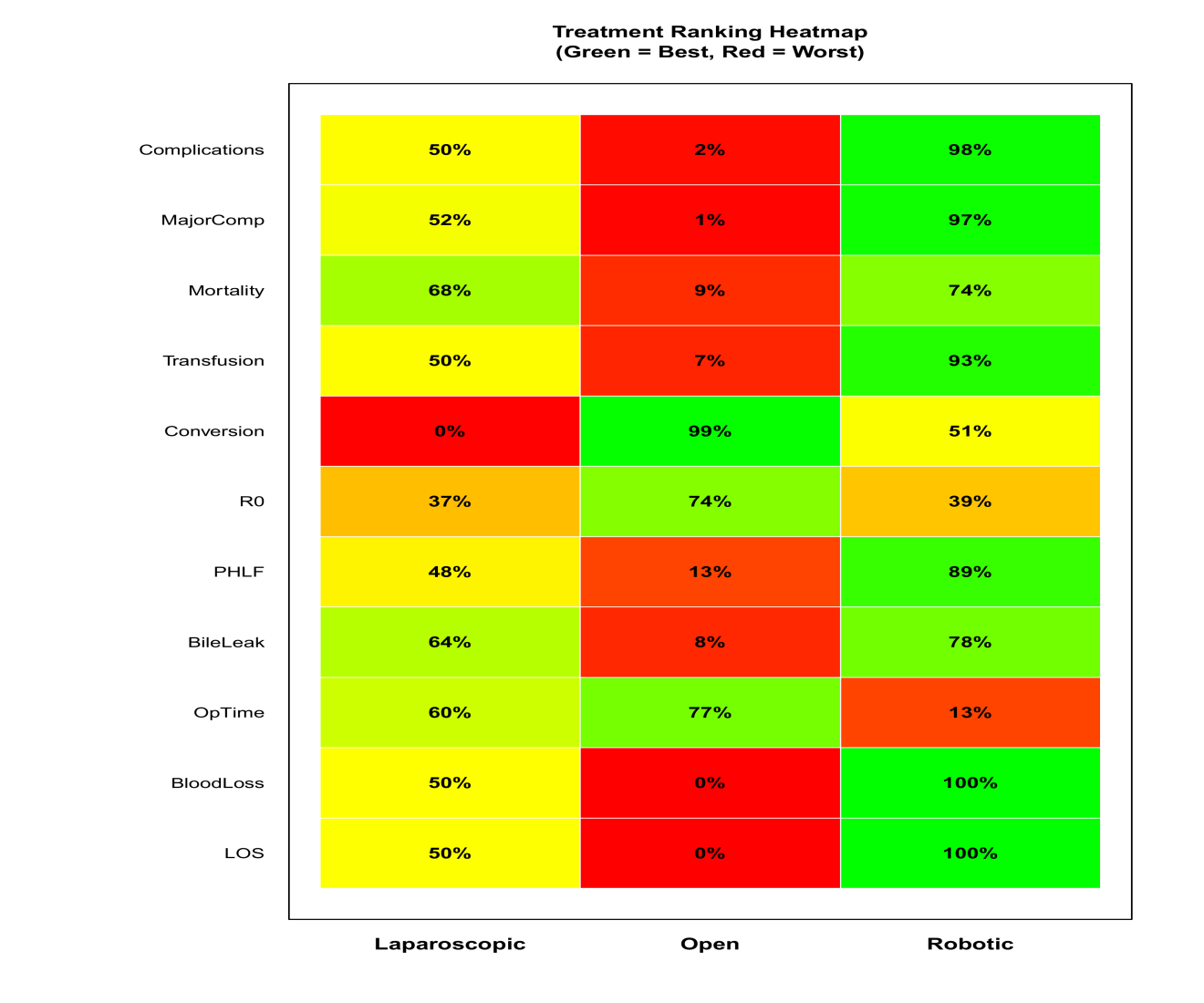


## SUPPLEMENTARY FIGURE S2

### P-Score Bar Chart for Treatment Rankings

Bar chart displaying P-scores for robotic, laparoscopic, and open hepatectomy across all 11 outcomes. P-scores range from 0 (worst) to 1 (best) and represent the probability of being the best treatment.


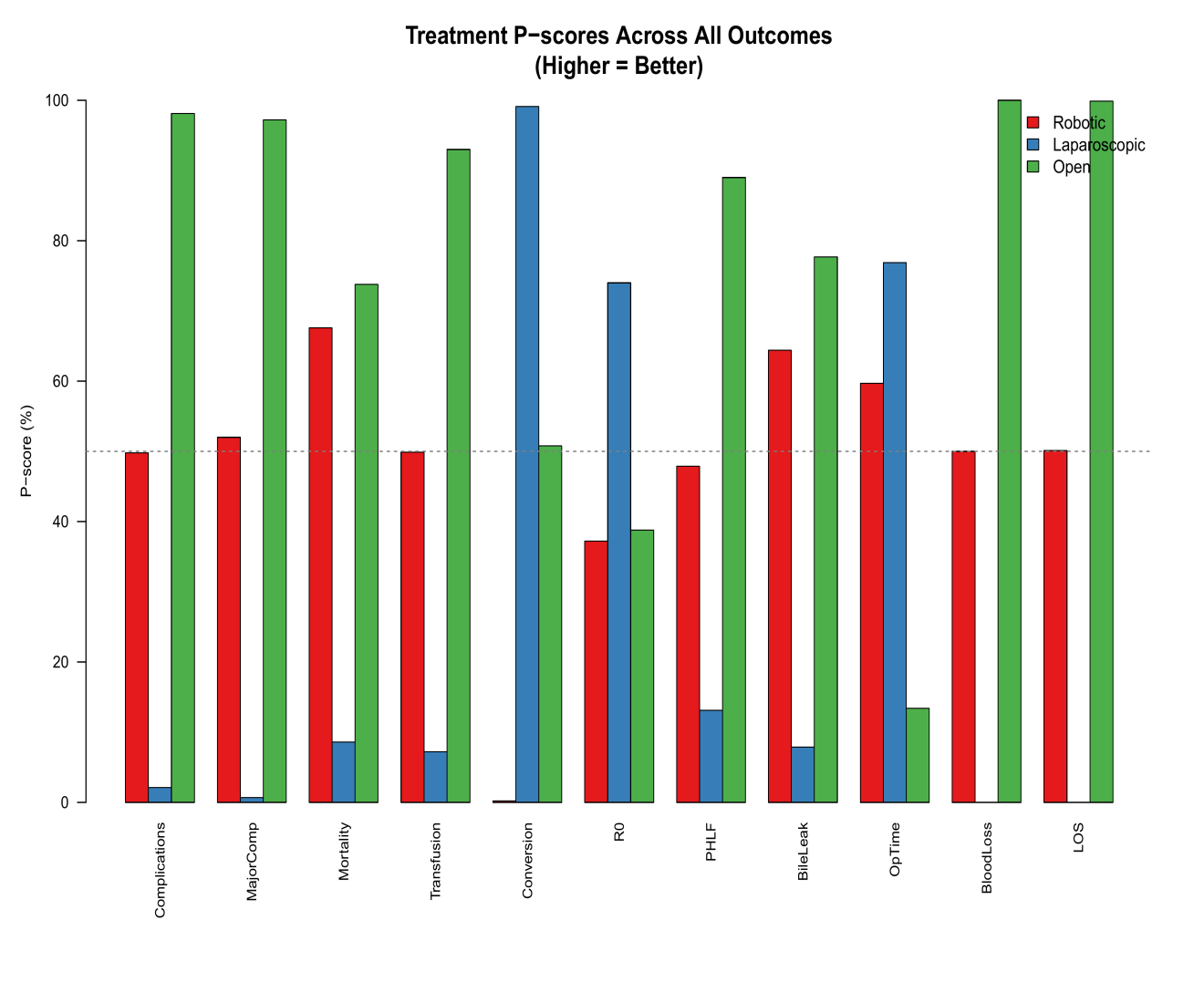


## SUPPLEMENTARY FIGURE S3

### Rankograms (Cumulative Ranking Probability)

Rankograms showing the cumulative probability of each treatment being ranked at each position for all outcomes. The area under the curve (SUCRA) summarizes the ranking probabilities.


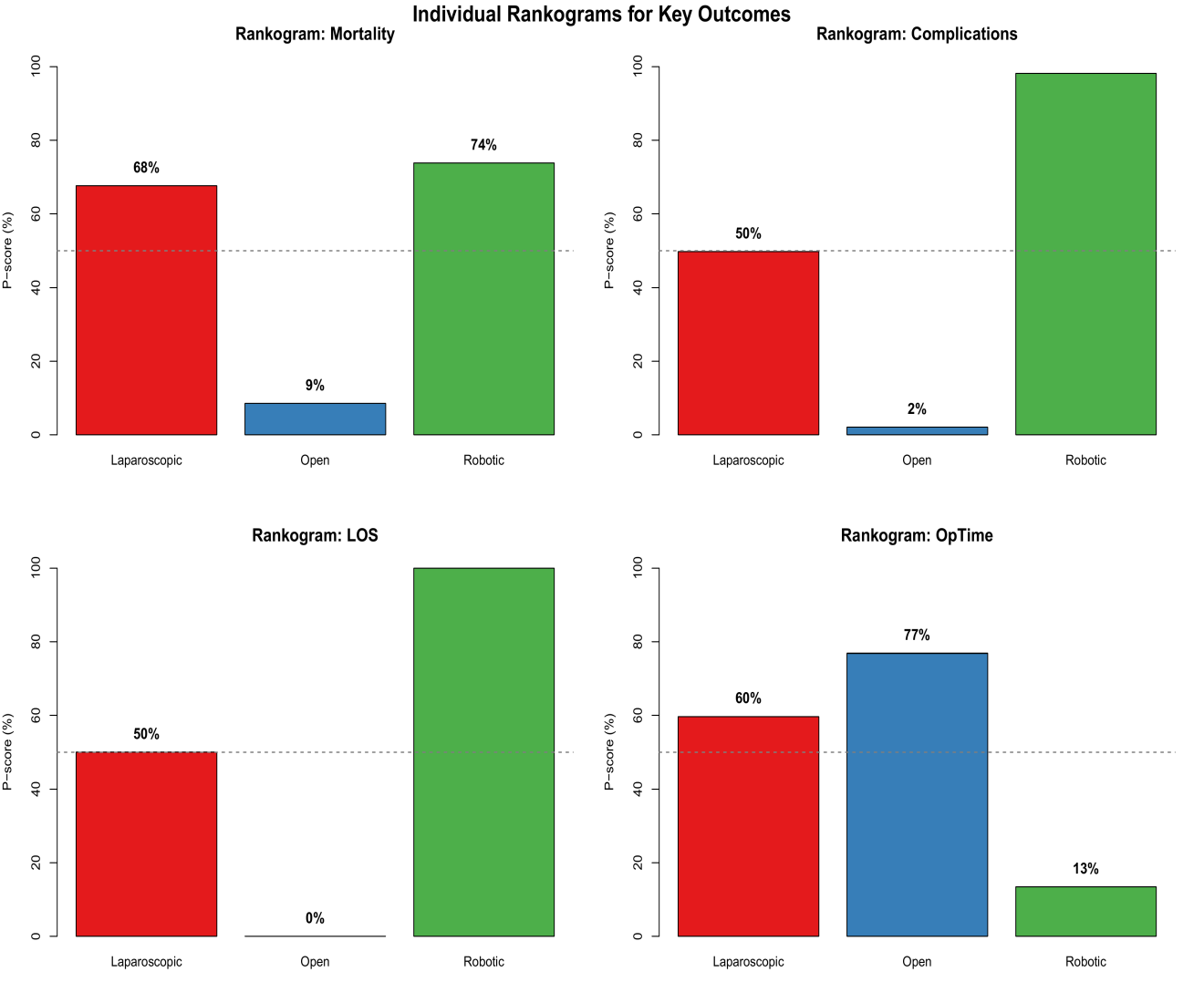


## SUPPLEMENTARY FIGURE S4

### Node-Splitting Analysis for Inconsistency Assessment

Node-splitting analysis comparing direct and indirect evidence for each pairwise comparison. P-values > 0.05 indicate no significant inconsistency between direct and indirect evidence, supporting the validity of the network meta-analysis.

S4A. Node-Splitting: Overall Complications


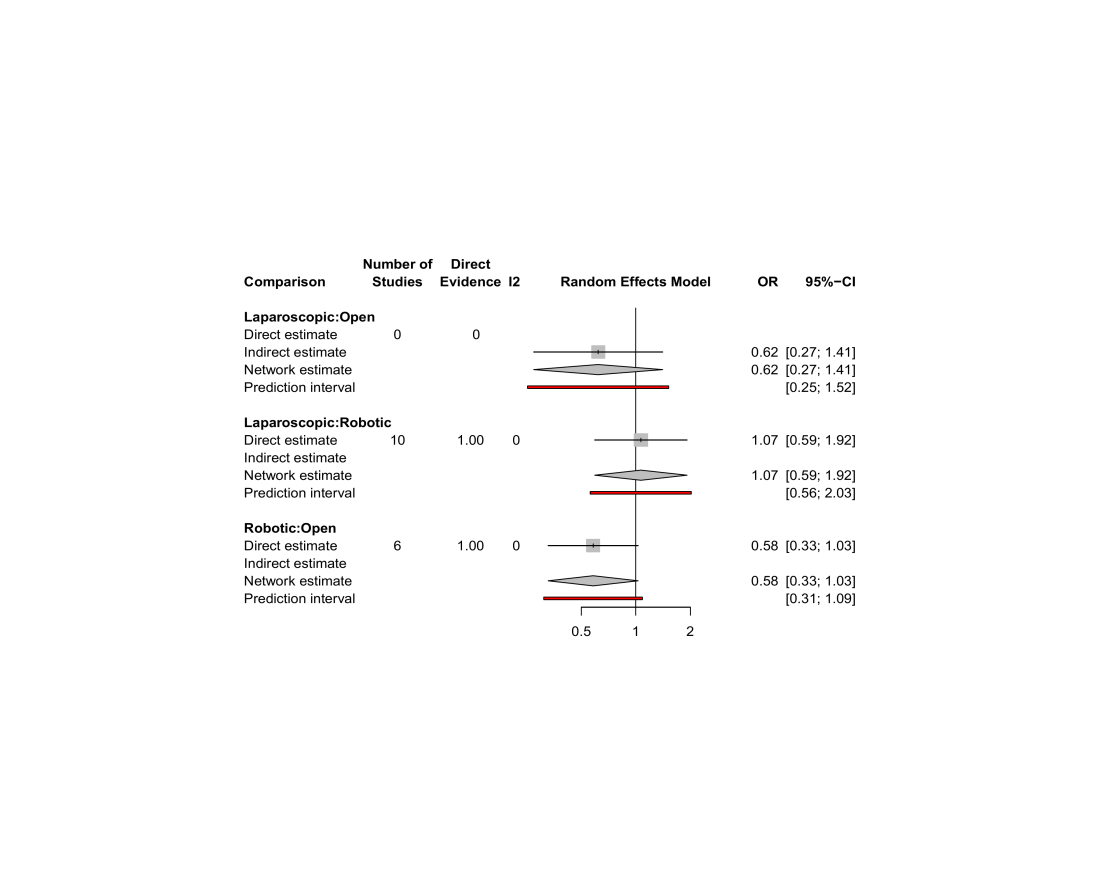


S4B. Node-Splitting: Major Complications


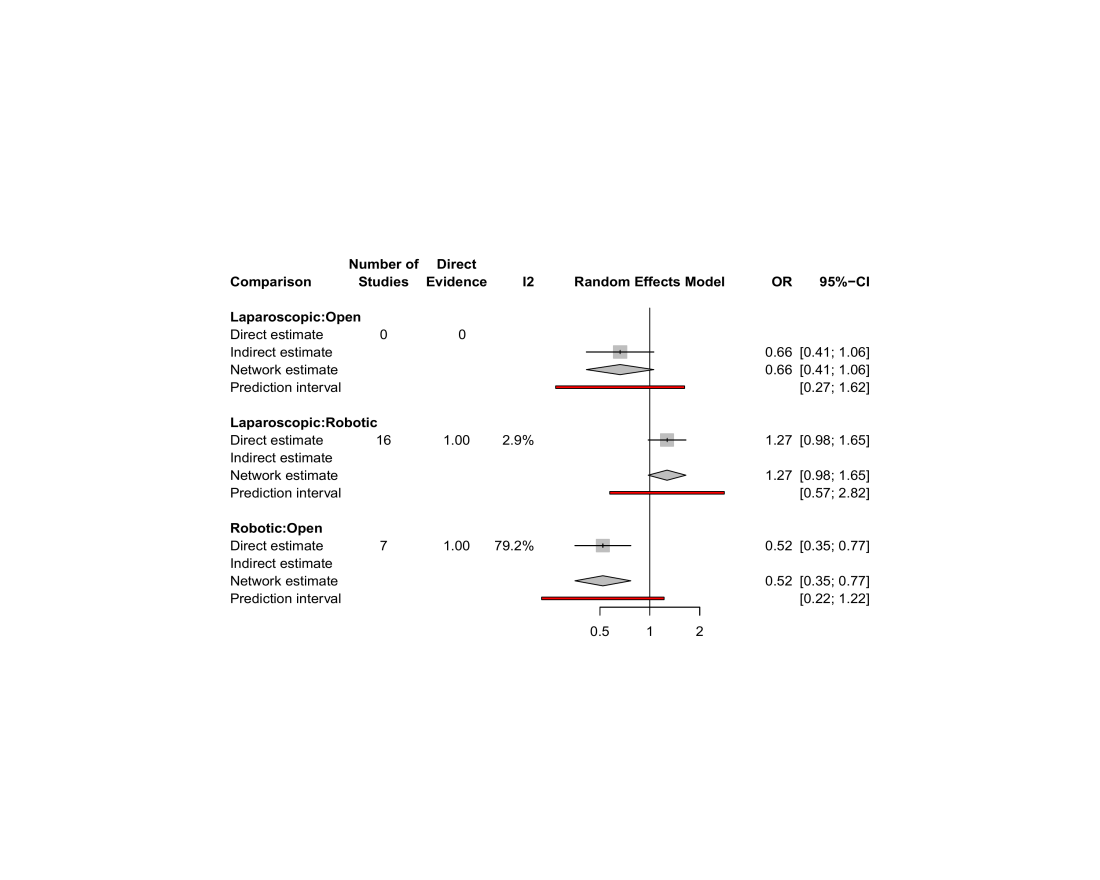


S4C. Node-Splitting: 90-Day Mortality


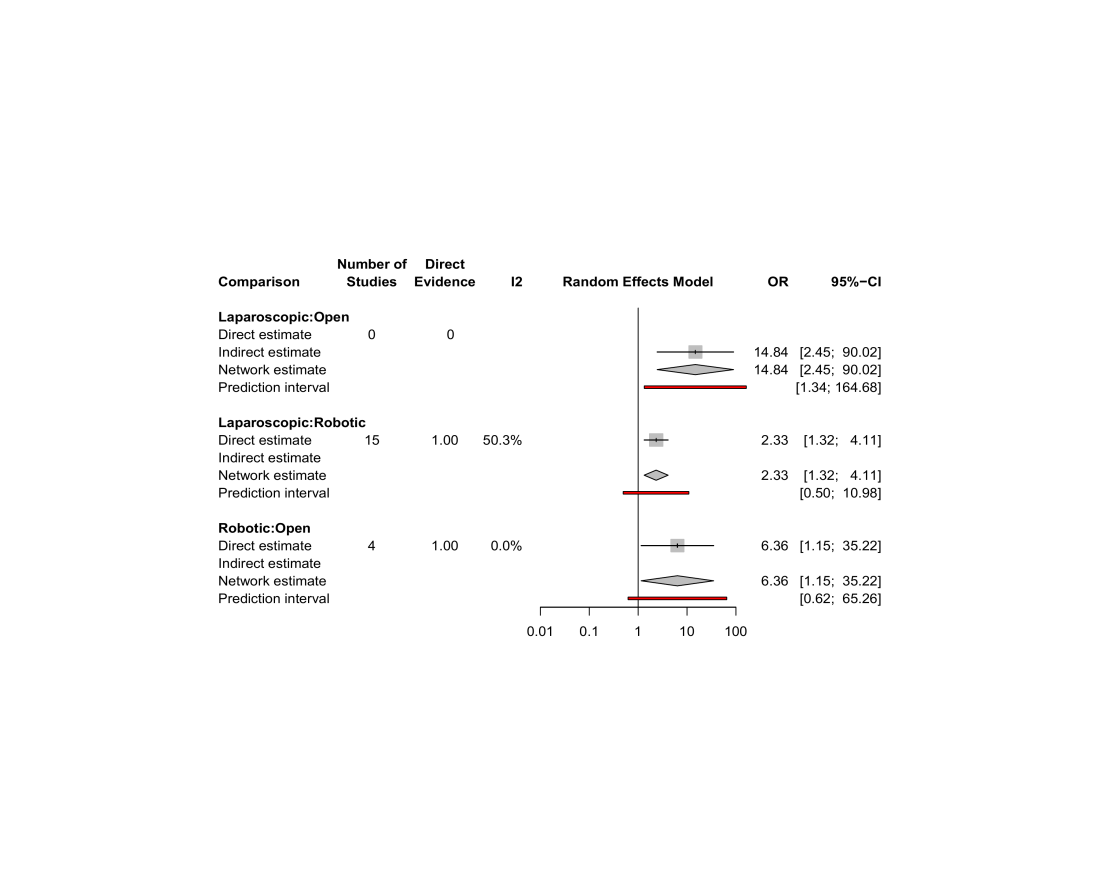


S4D. Node-Splitting: Blood Transfusion


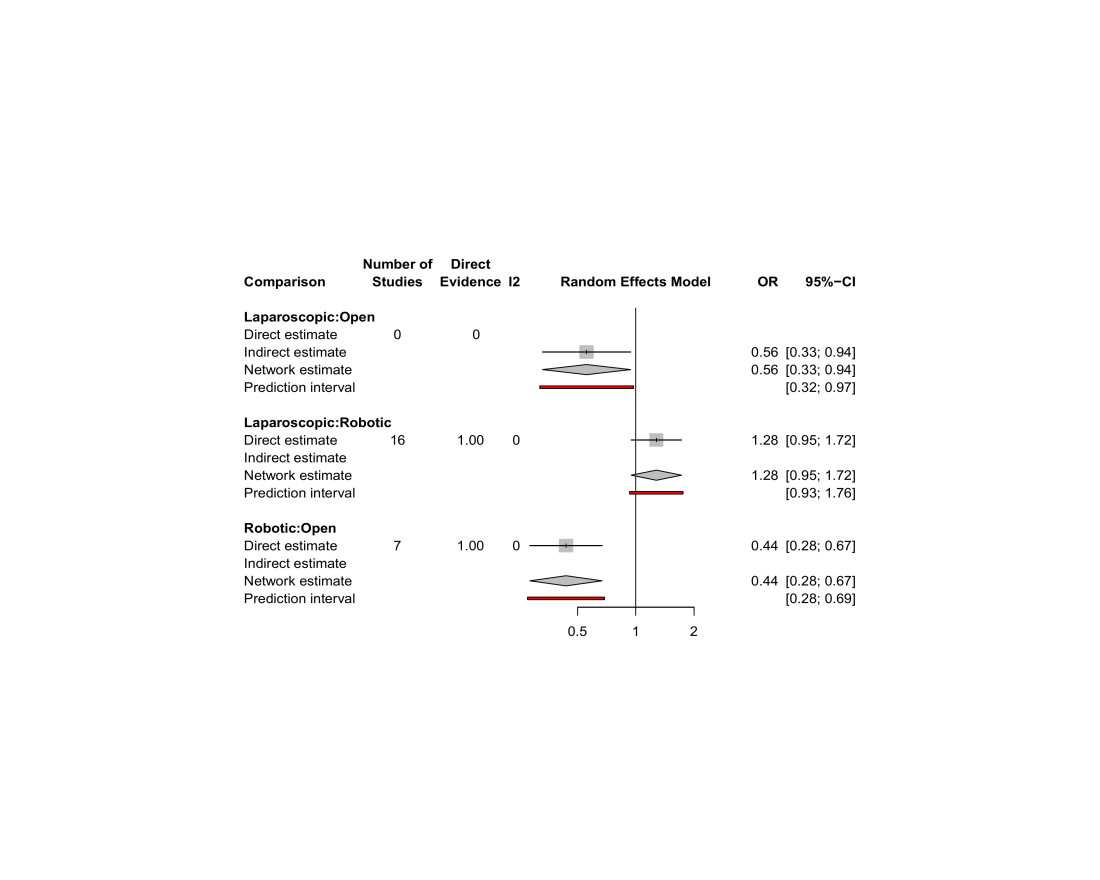


S4E. Node-Splitting: Conversion Rate


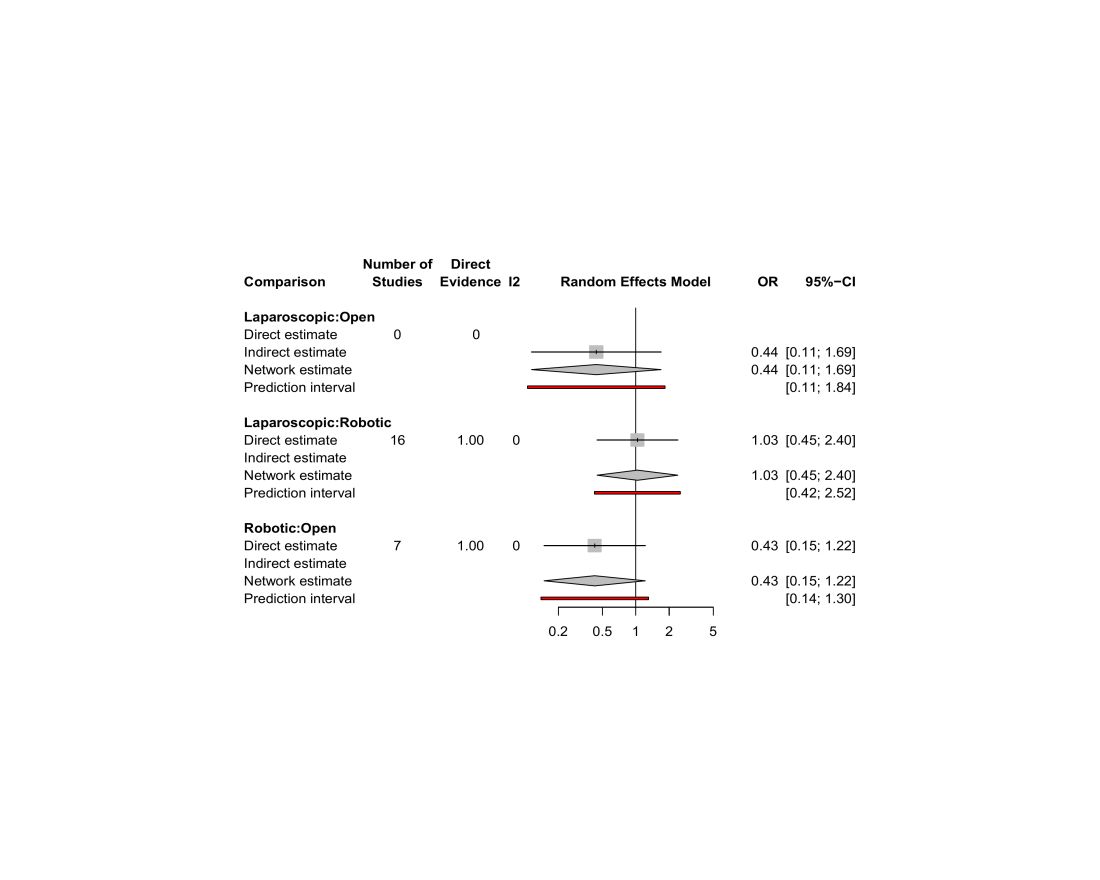


S4F. Node-Splitting: R0 Resection


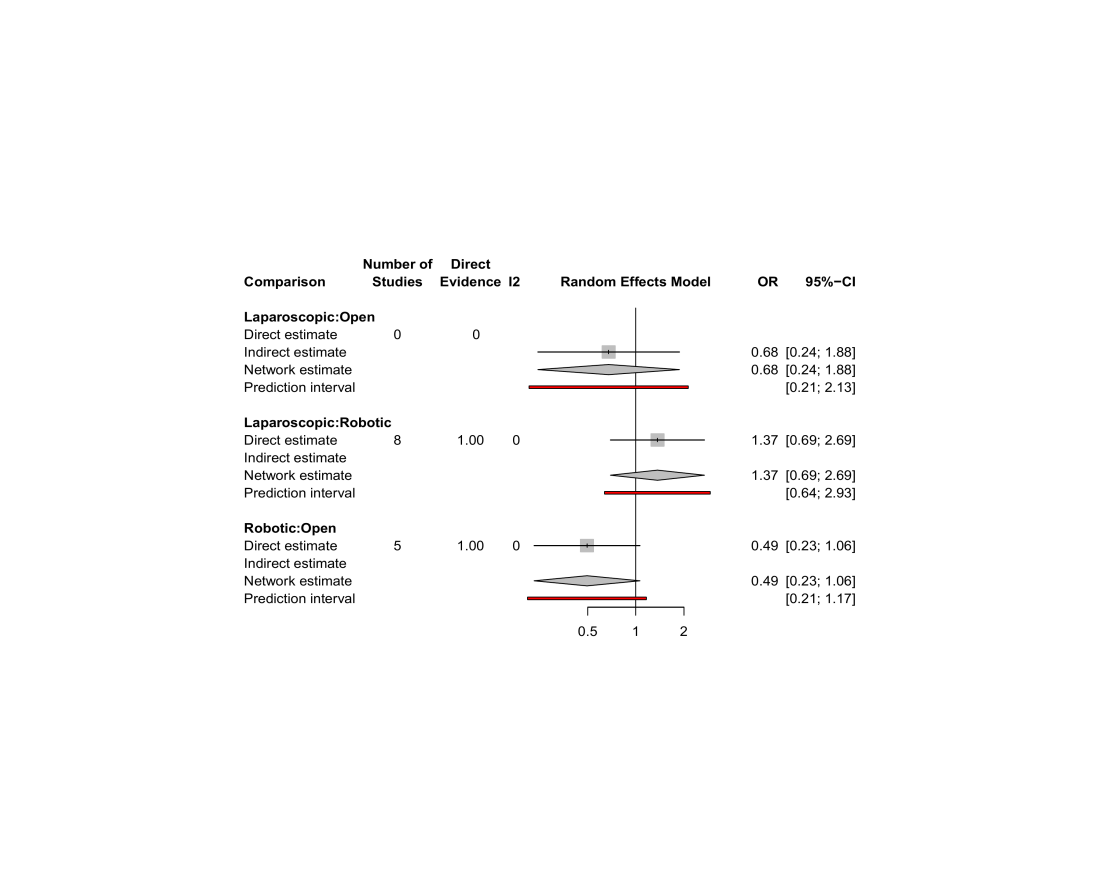


S4G. Node-Splitting: PHLF


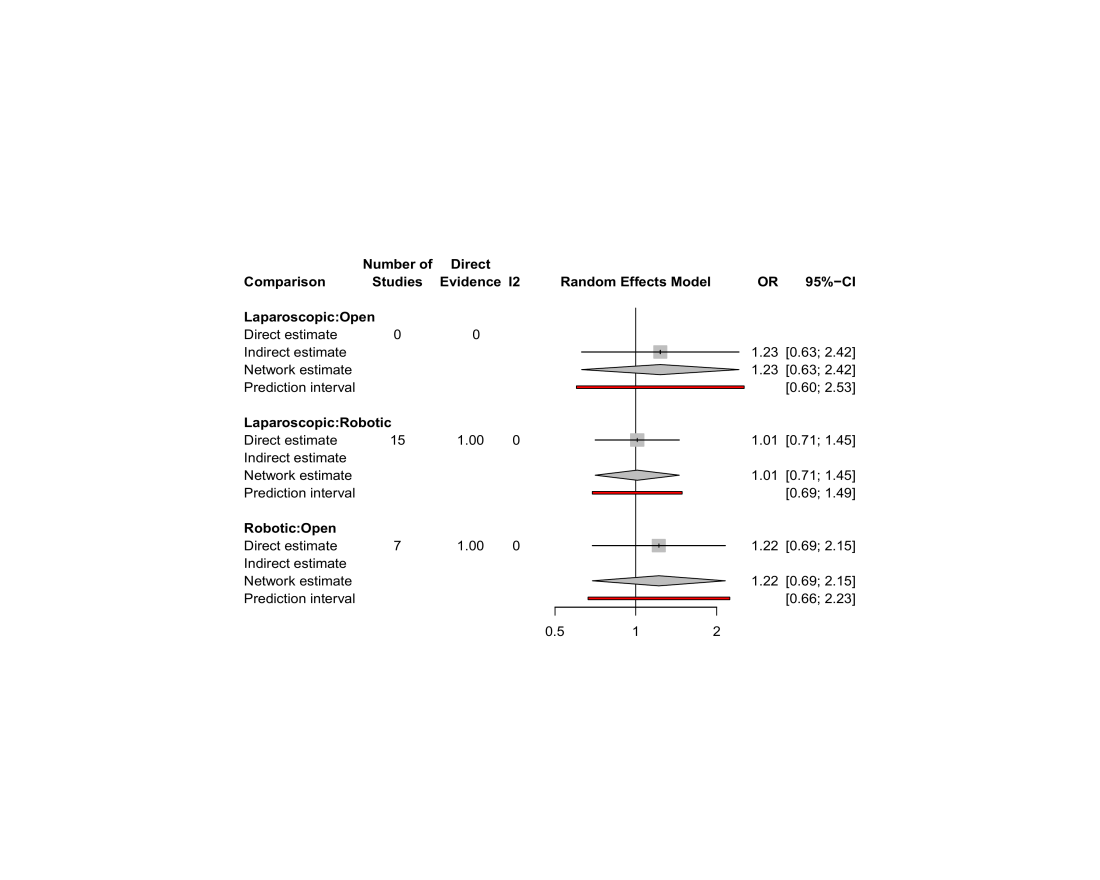


S4H. Node-Splitting: Bile Leak


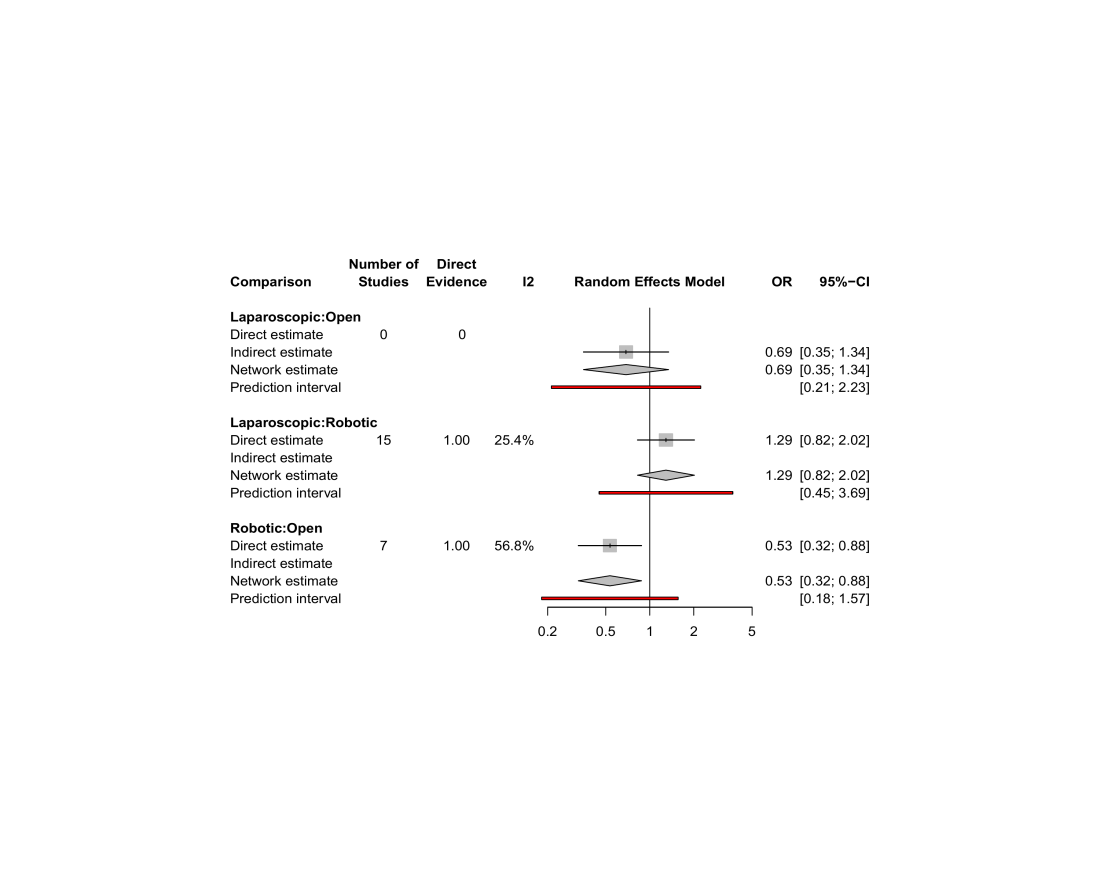


## SUPPLEMENTARY FIGURE S5

### Funnel Plots for Publication Bias Assessment

Comparison-adjusted funnel plots for assessing publication bias and small-study effects. Symmetrical distribution around the vertical line suggests absence of significant publication bias.

S5A. Funnel Plot: Overall Complications


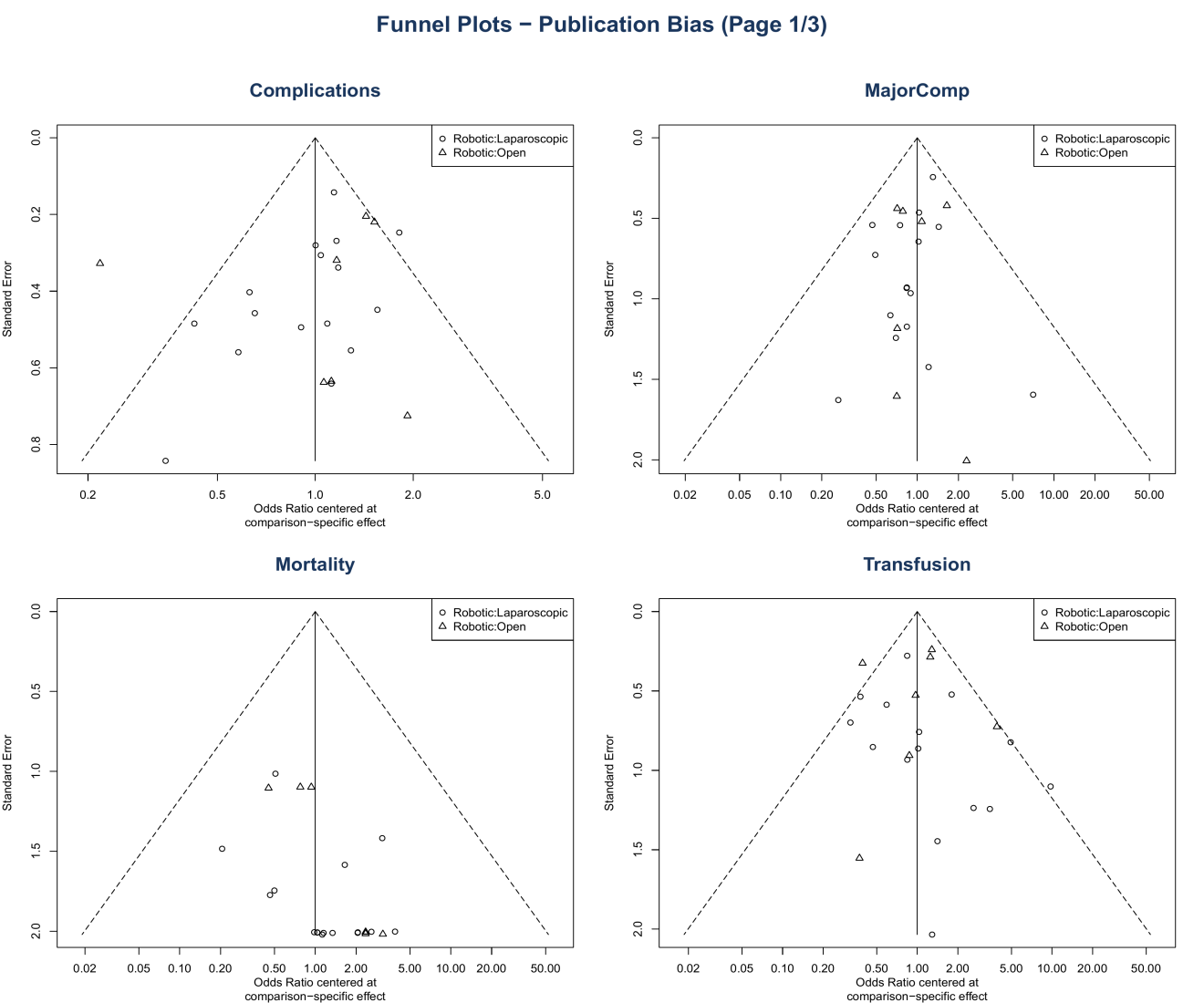


S5B. Funnel Plot: Major Complications


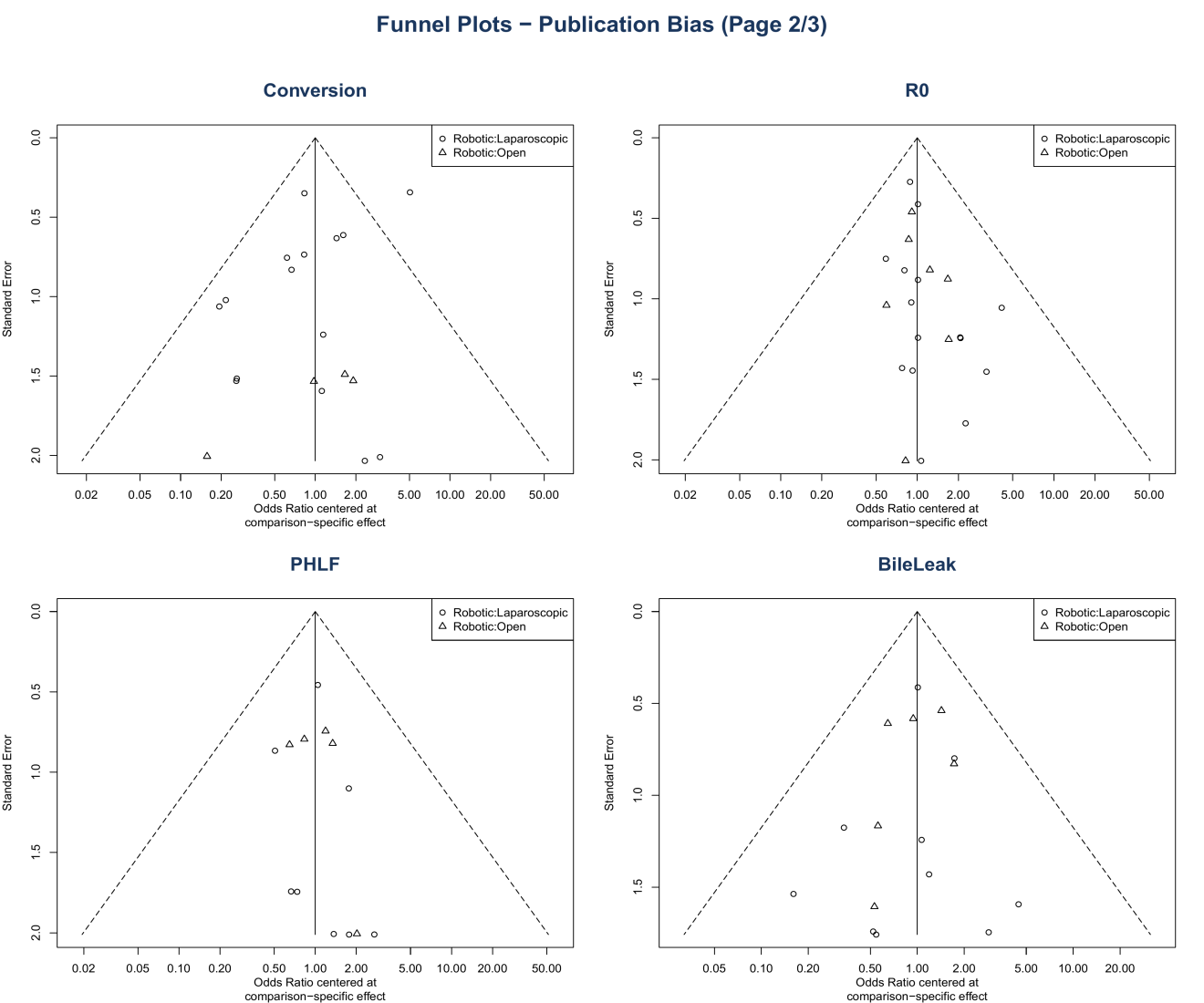


S5C. Funnel Plot: 90-Day Mortality


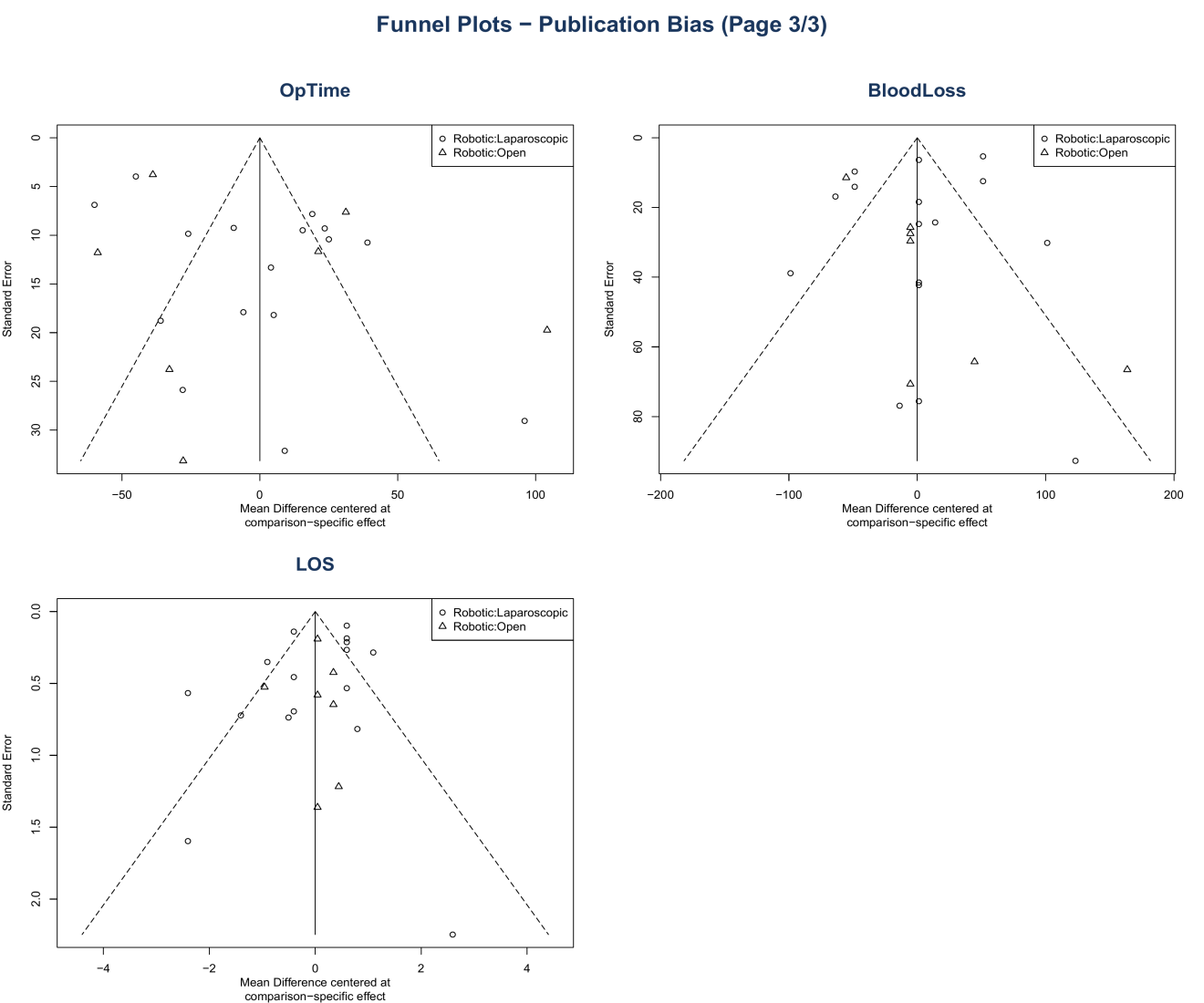


S5D. Funnel Plot: Blood Transfusion


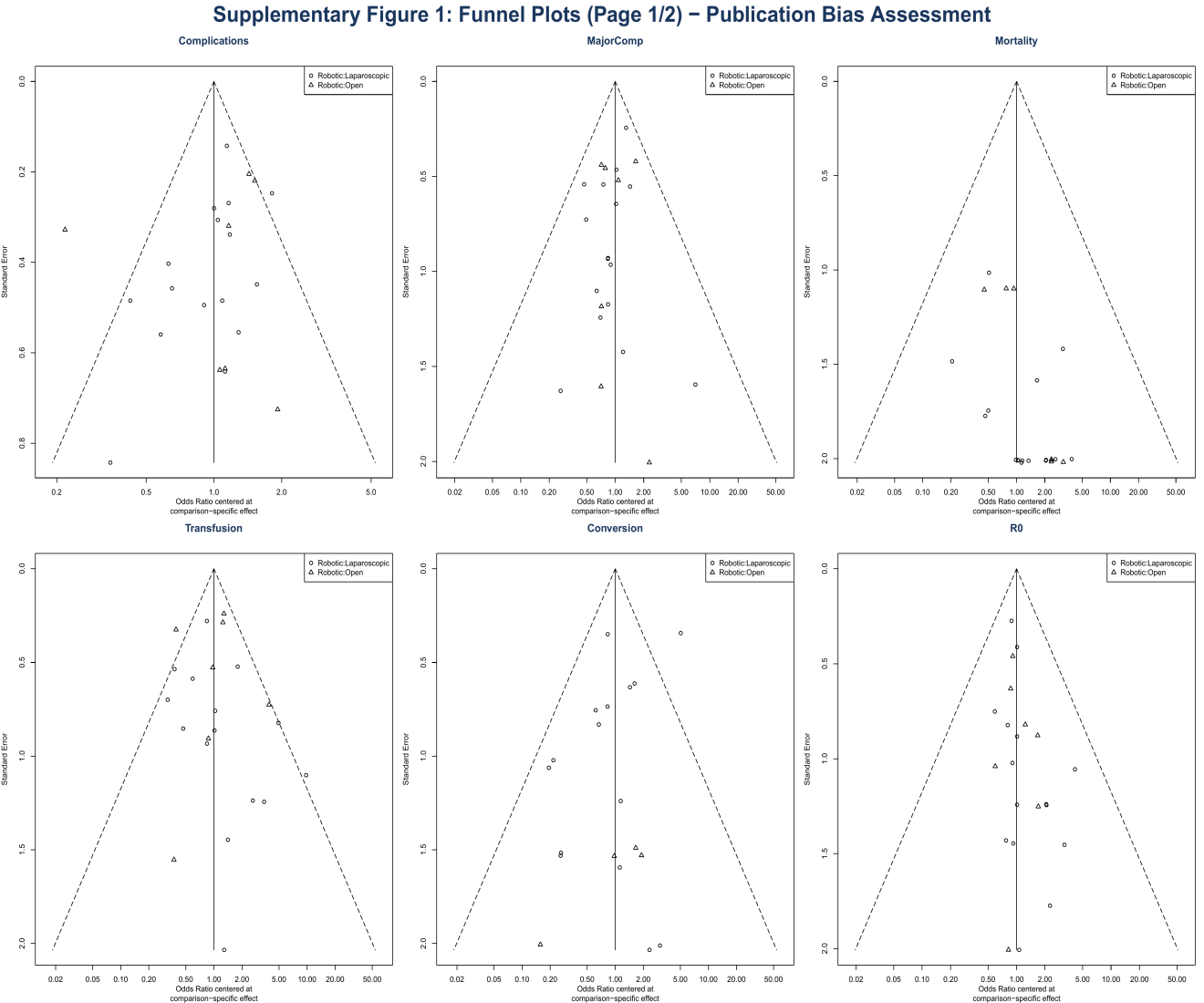


S5E. Funnel Plot: R0 Resection


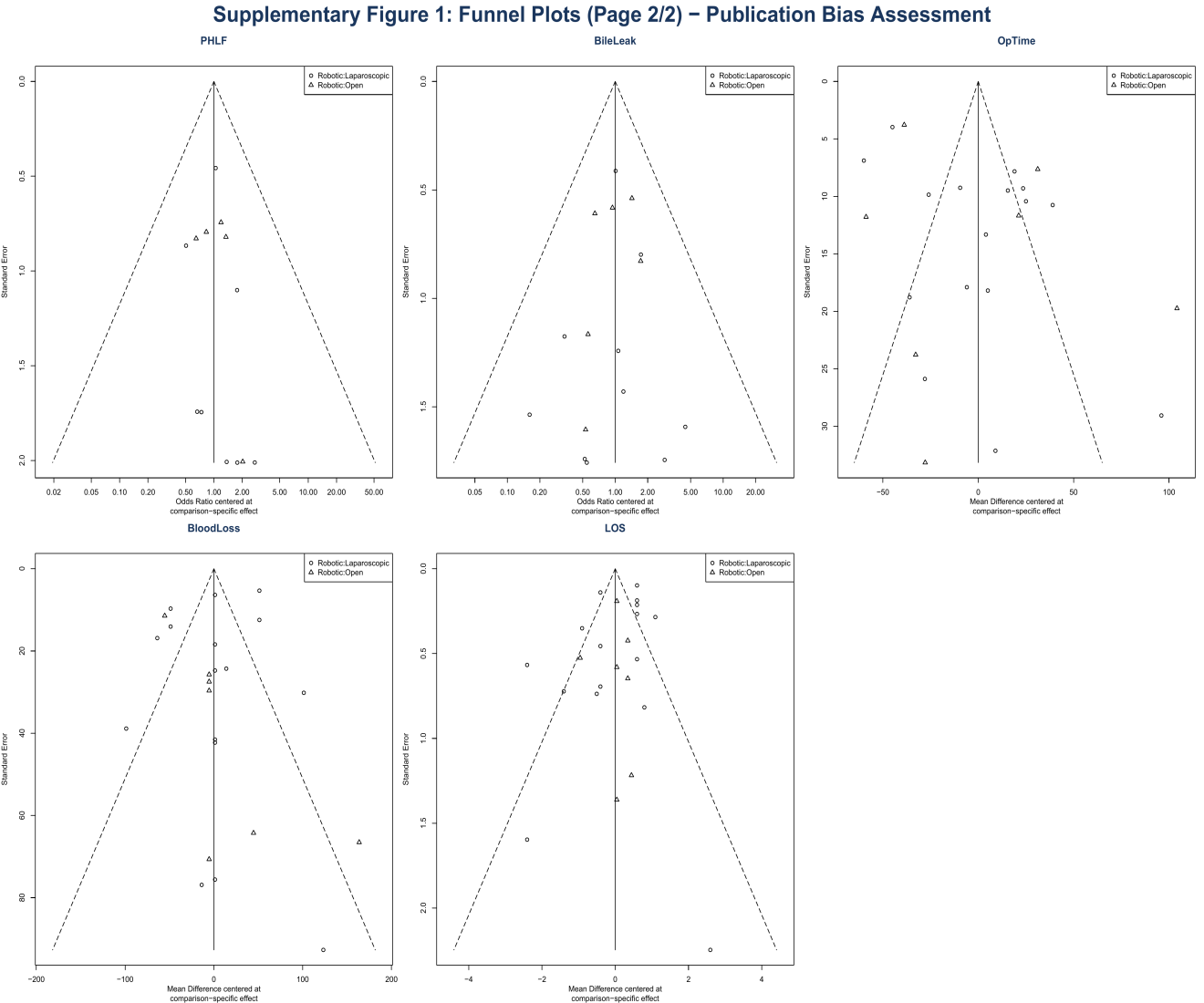


## SUPPLEMENTARY FIGURE S6

### Long-Term Survival Outcomes

Forest plots comparing long-term survival outcomes including 1-year, 3-year, and 5-year overall survival (OS) and recurrence-free survival (RFS) rates between robotic and conventional (laparoscopic/open) hepatectomy.

S6A. 1-Year Overall Survival (OS)


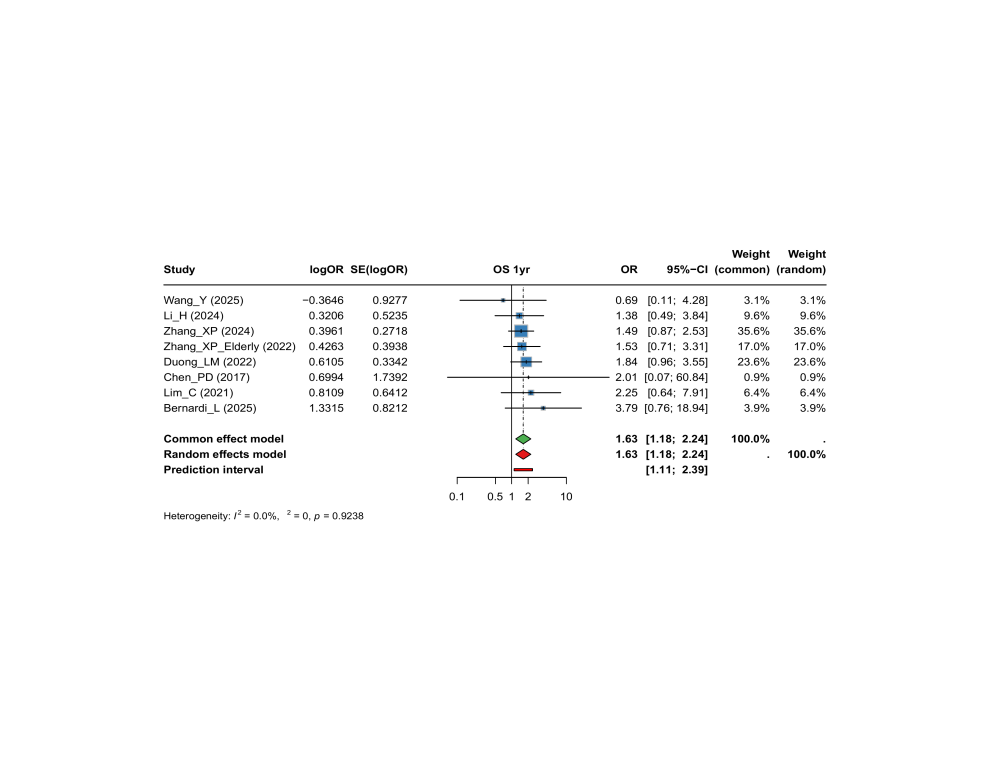


S6B. 3-Year Overall Survival (OS)


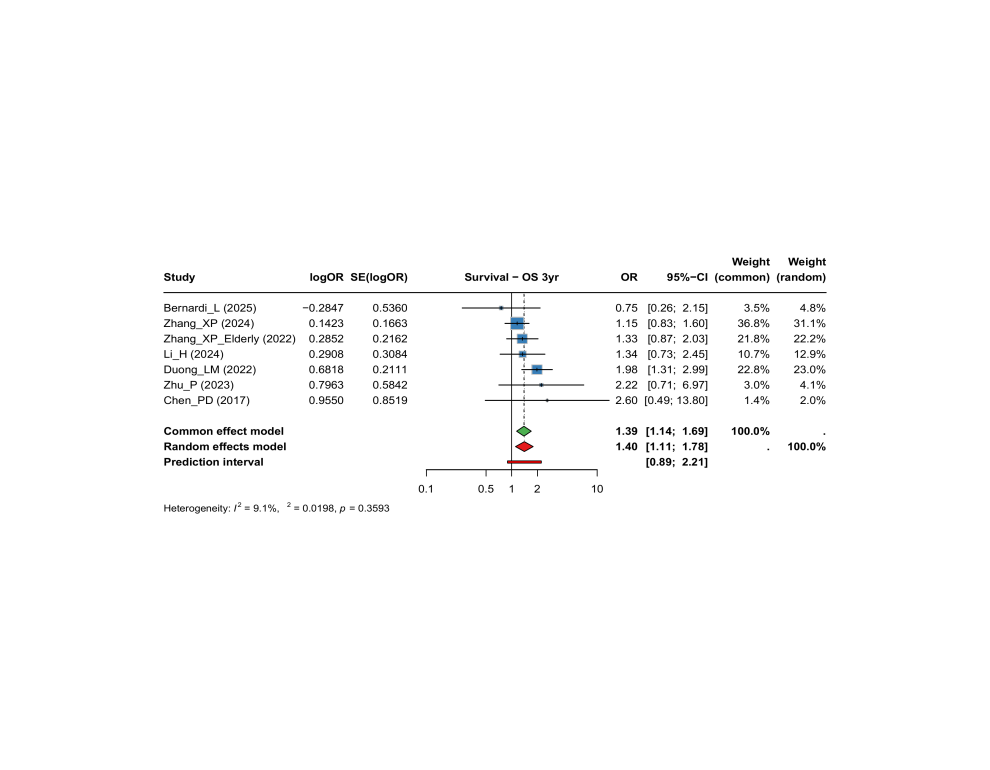


S6C. 5-Year Overall Survival (OS)


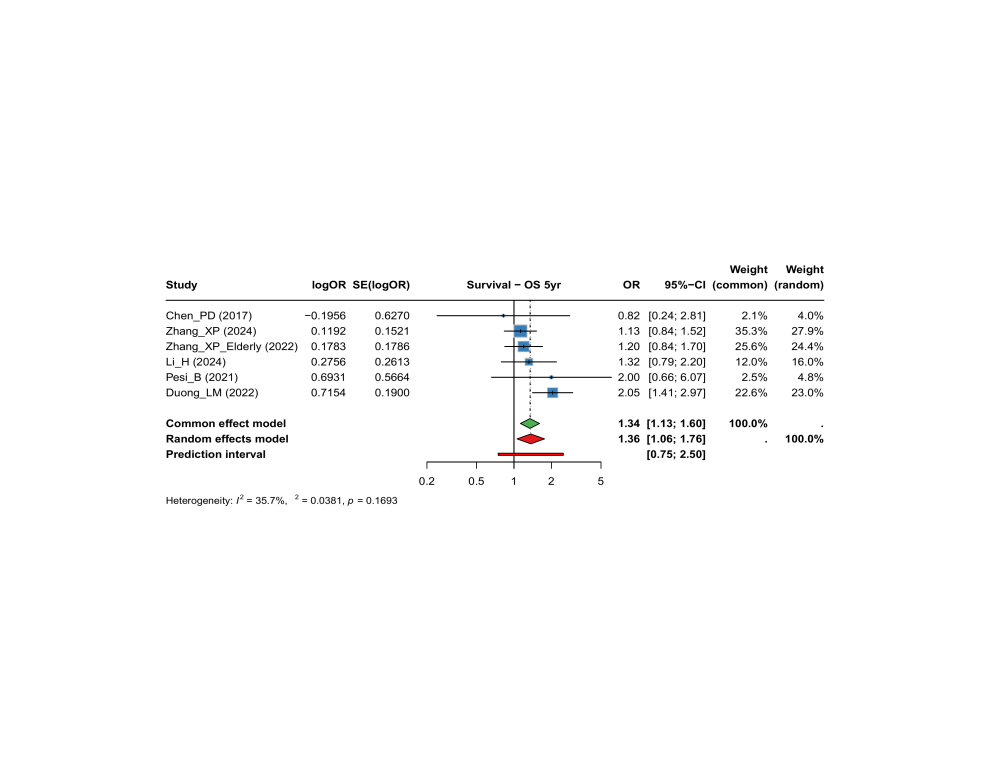


S6D. 1-Year Recurrence-Free Survival (RFS)


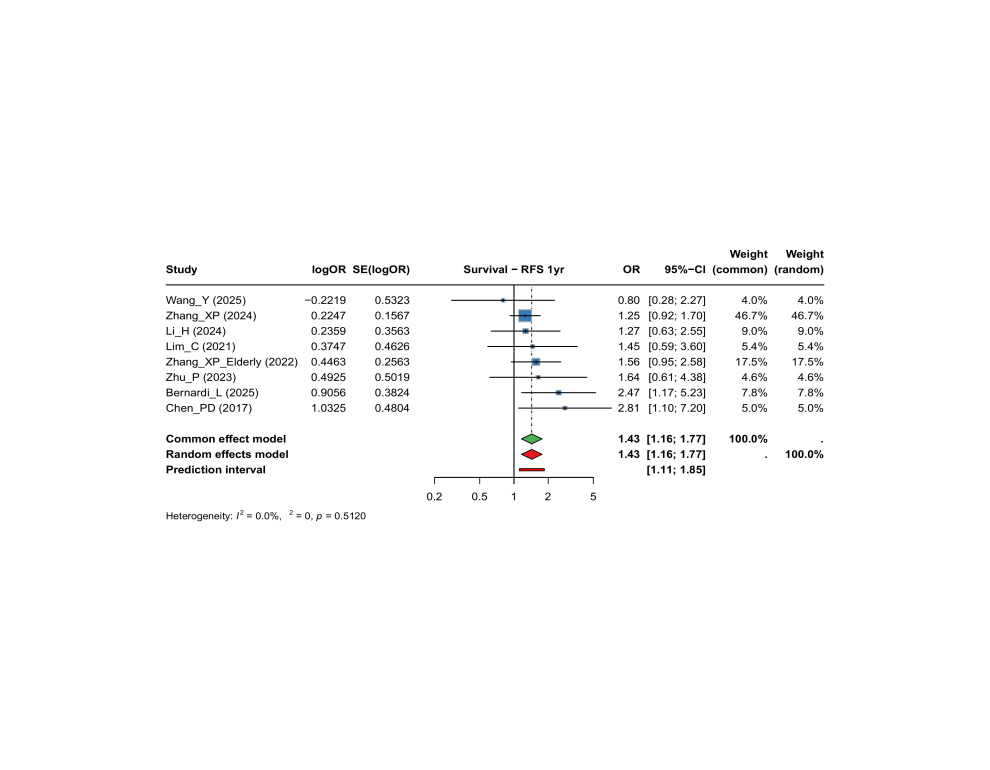


S6E. 3-Year Recurrence-Free Survival (RFS)


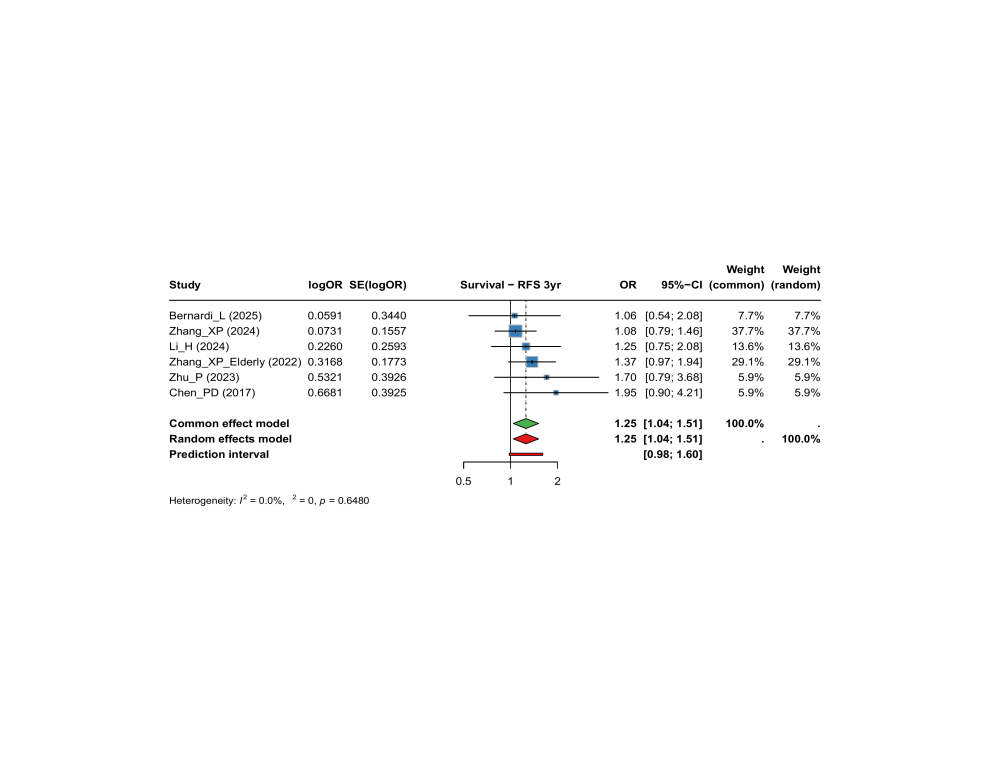


S6F. 5-Year Recurrence-Free Survival (RFS)


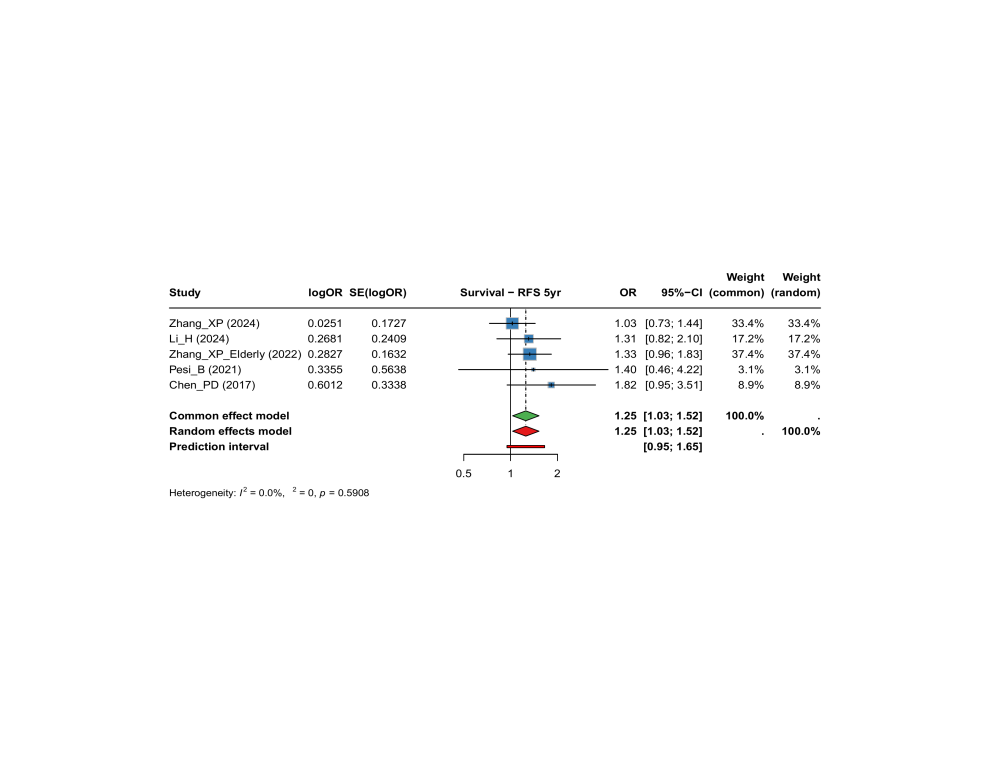


**SUPPLEMENTARY FIGURE S7**

**Subgroup Analysis Forest Plots**

Forest plots for all subgroup analyses comparing robotic, laparoscopic, and open hepatectomy. Results are presented for four subgroup classifications: (1) major vs minor hepatectomy, (2) publication year, (3) sample size, and (4) posterosuperior segment involvement. Each plot shows the effect estimate (OR for binary, MD for continuous outcomes) with 95% confidence intervals and p-values. Red markers and lines indicate statistically significant results (p < 0.05); blue markers indicate non-significant results.

**SUBGROUP 1: MAJOR vs MINOR HEPATECTOMY**

Nine studies (39.1%) reported major hepatectomy proportions. Studies classified as predominantly major (≥50% major resections; n=2: Zhang 2024 [68.5%], Huang 2024 [100%]) or predominantly minor (<50%; n=7).

**S7A. Major vs Minor Hepatectomy: Robotic vs Open — Binary Outcomes**


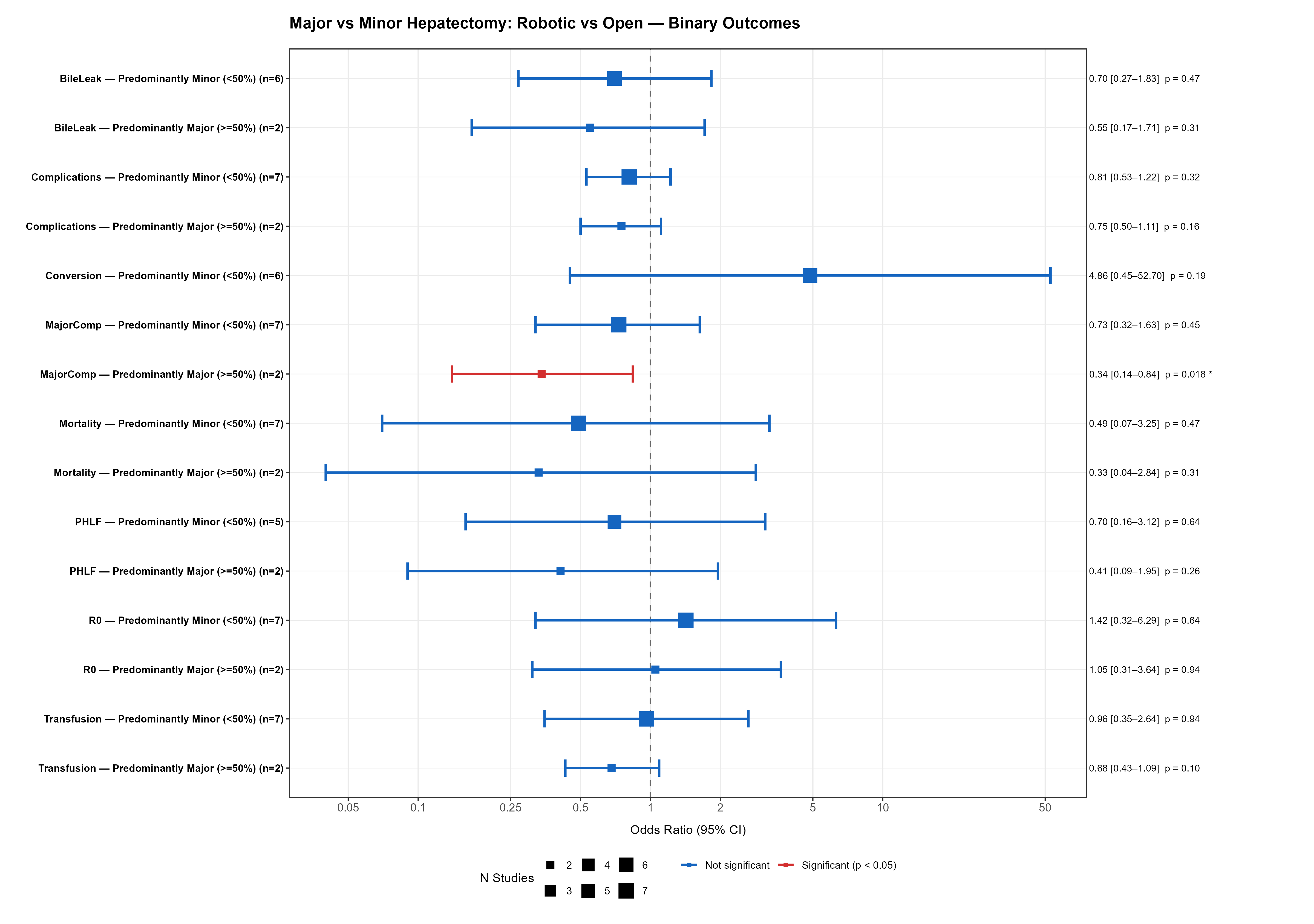


*Supplementary Figure S7A. Subgroup forest plot comparing robotic vs open hepatectomy for binary outcomes stratified by hepatectomy extent. Major complications were significantly lower with robotic surgery in the major hepatectomy subgroup (OR 0.34, 95% CI 0.14–0.84, p = 0.019).*

**S7B. Major vs Minor Hepatectomy: Robotic vs Open — Continuous Outcomes**


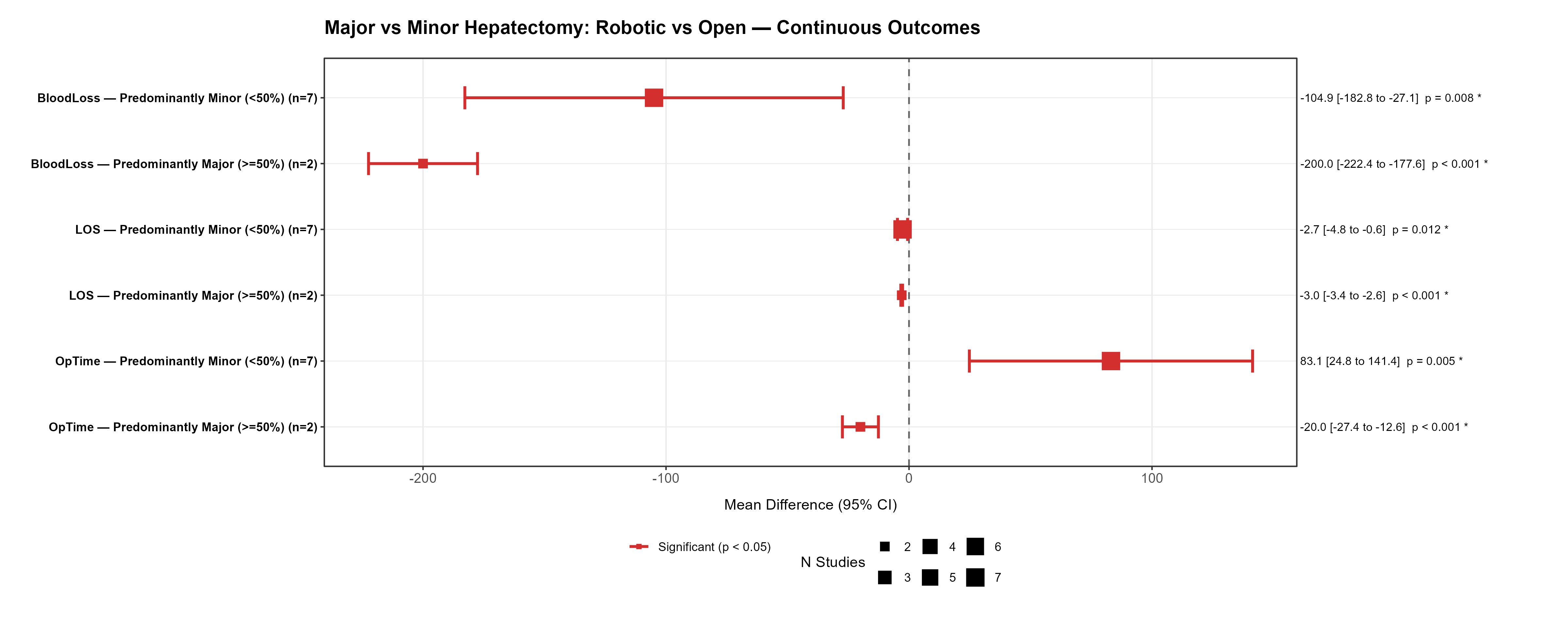


*Supplementary Figure S7B. Continuous outcomes (operative time, blood loss, length of stay) by hepatectomy extent. Blood loss reduction was nearly double in major resections (MD −200.0 mL) compared with minor (MD −104.9 mL). Operative time was shorter with robotic in major but longer in minor hepatectomy.*

**S7C. Major vs Minor Hepatectomy: Laparoscopic vs Robotic — Binary Outcomes**


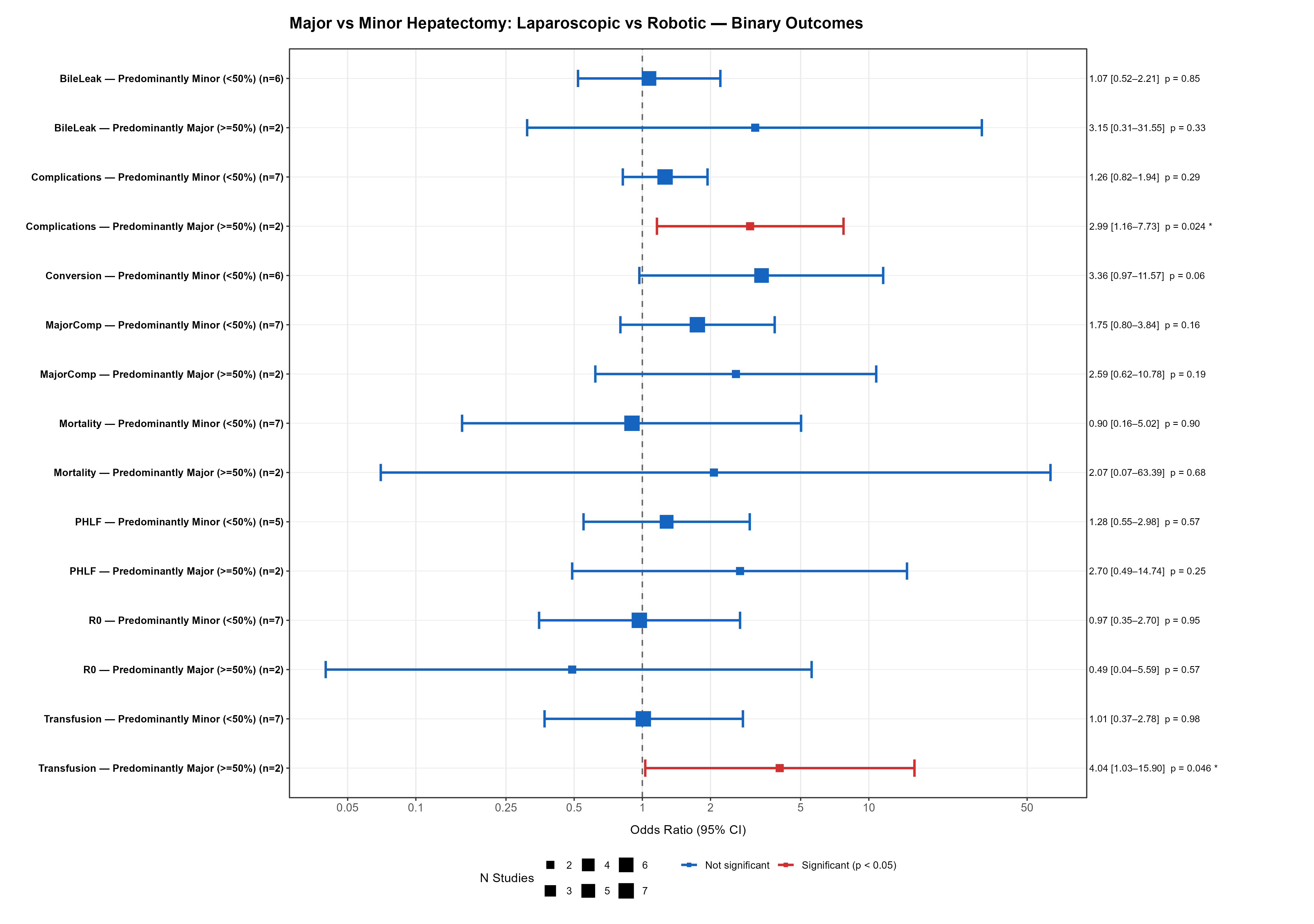


*Supplementary Figure S7C. Laparoscopic vs robotic hepatectomy for binary outcomes. In major hepatectomy, laparoscopic had significantly higher overall complication rates (OR 2.99, p = 0.024) and transfusion rates (OR 4.04, p = 0.048) compared with robotic.*

**S7D. Major vs Minor Hepatectomy: Laparoscopic vs Open — Binary Outcomes**


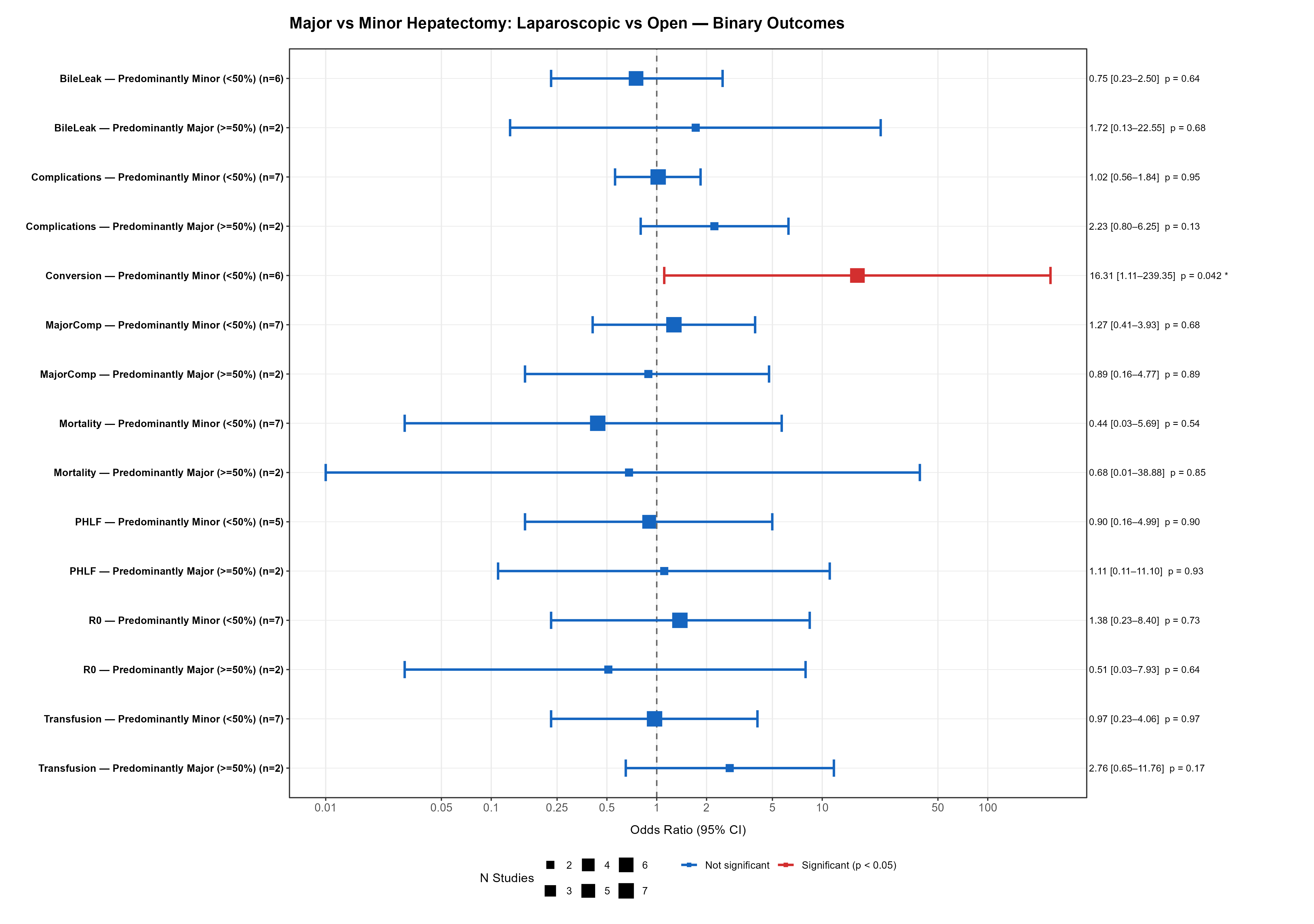


*Supplementary Figure S7D. Laparoscopic vs open hepatectomy for binary outcomes stratified by hepatectomy extent.*

**S7E. Major vs Minor Hepatectomy: Laparoscopic vs Robotic — Continuous Outcomes**


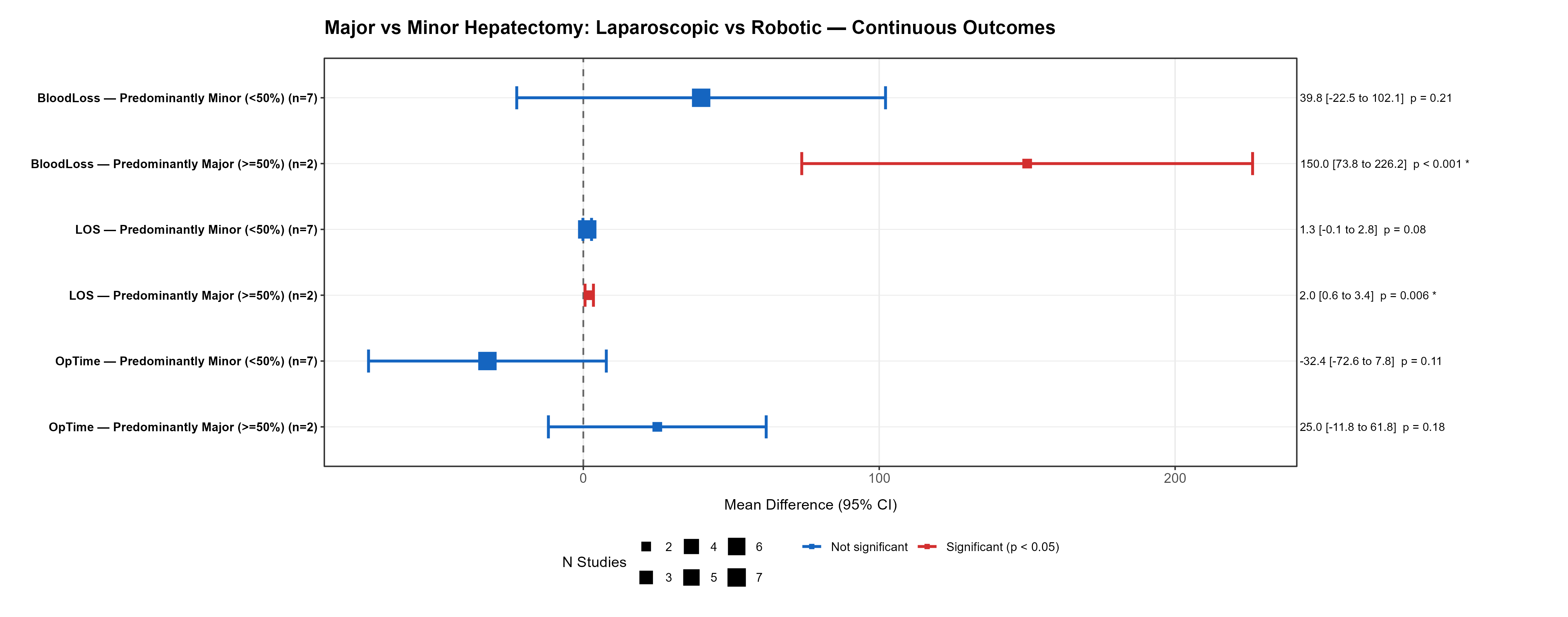


*Supplementary Figure S7E. Continuous outcomes comparing laparoscopic vs robotic by hepatectomy extent. Blood loss was significantly higher with laparoscopic vs robotic in major hepatectomy (MD +150.0 mL, p < 0.001).*

**SUBGROUP 2: PUBLICATION YEAR**

Studies stratified by publication year: Recent (≥2022, n=17) vs Earlier (<2022, n=6).

**S7F. Publication Year: Robotic vs Open — Binary Outcomes**


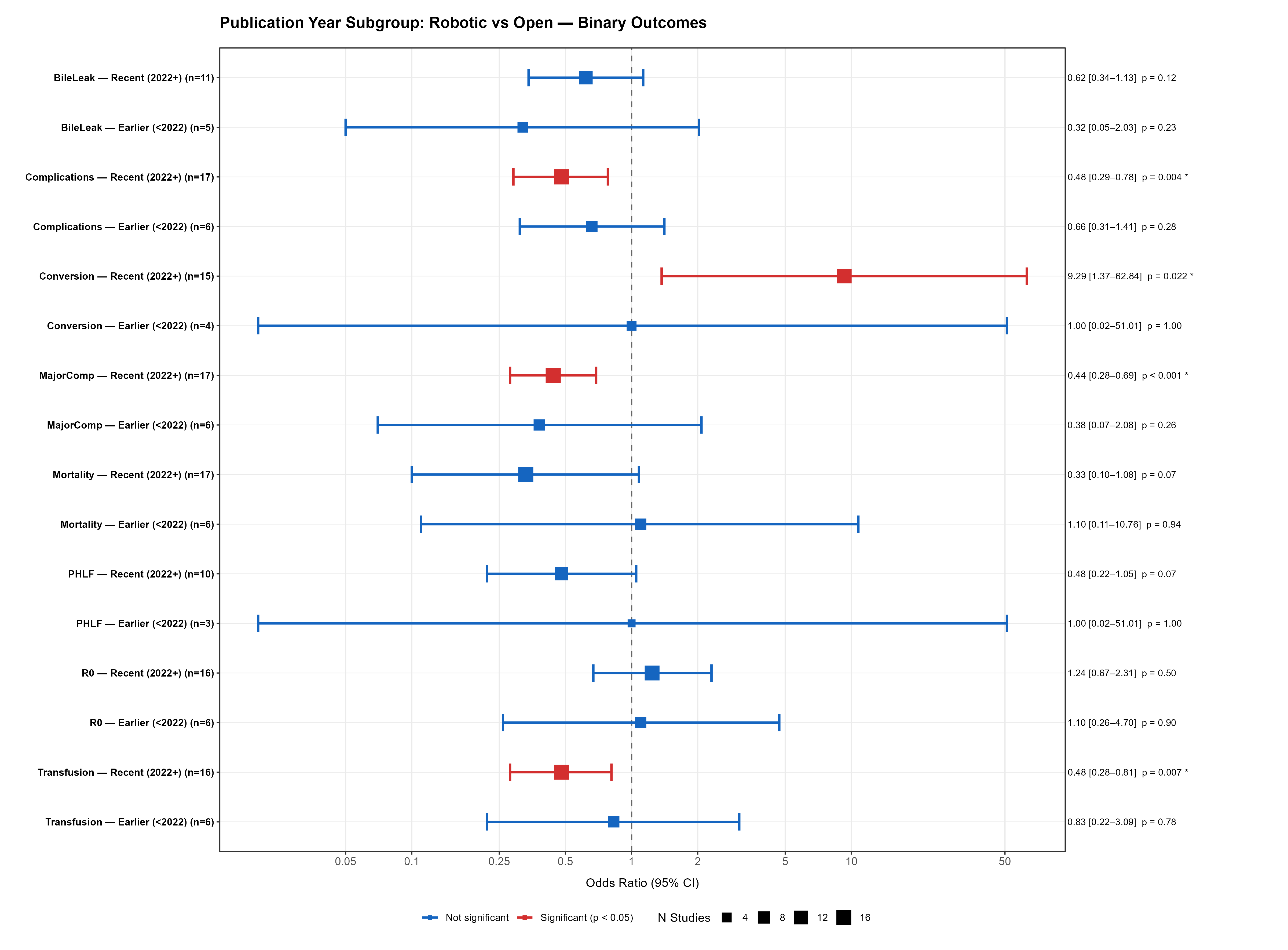


*Supplementary Figure S7F. Robotic vs open outcomes by publication year. Recent studies (2022+) showed significant reductions in complications (OR 0.48), major complications (OR 0.44), and transfusion (OR 0.48).*

**S7G. Publication Year: Laparoscopic vs Open — Binary Outcomes**


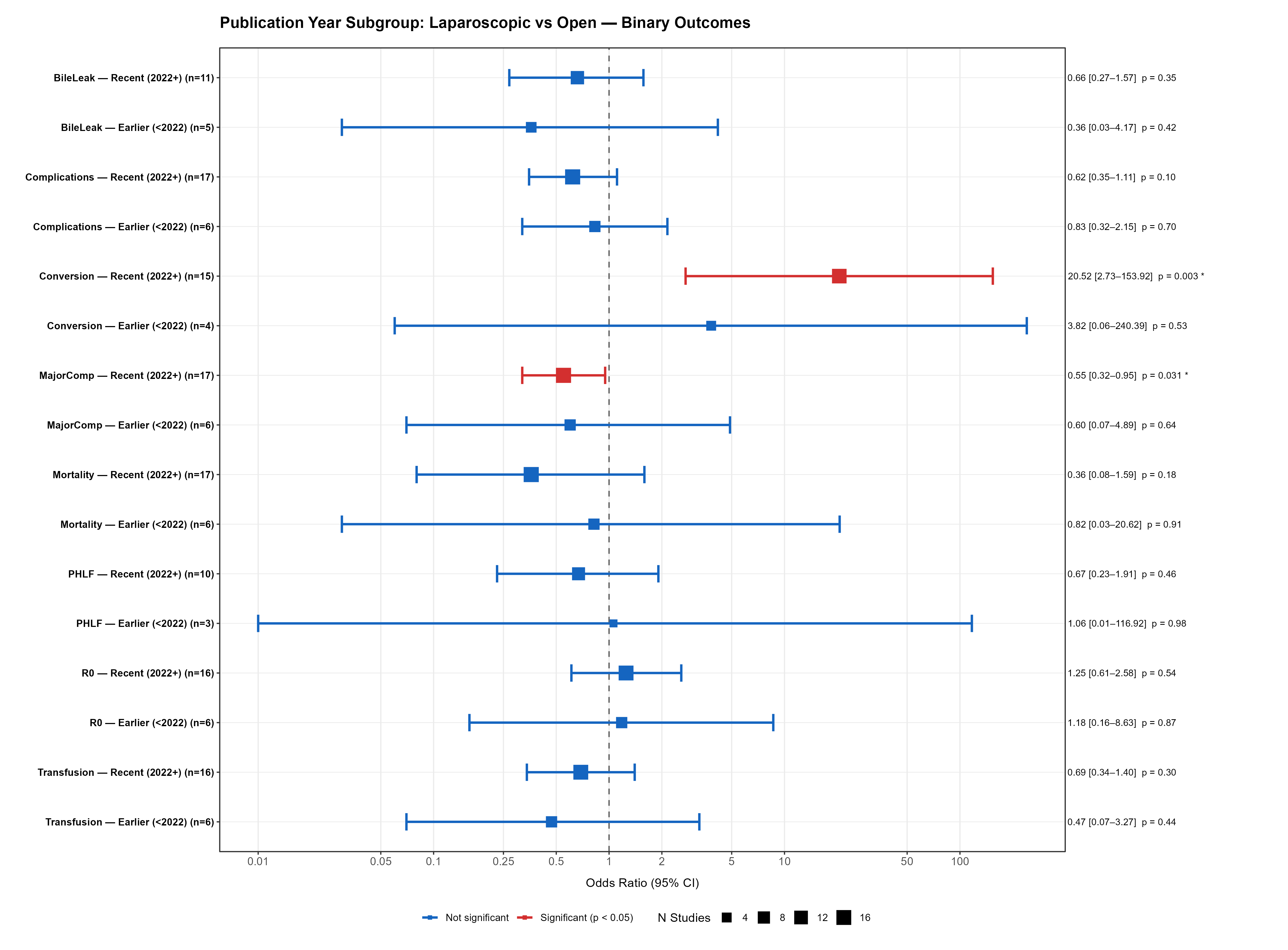


*Supplementary Figure S7G. Laparoscopic vs open outcomes by publication year.*

**SUBGROUP 3: SAMPLE SIZE**

Studies stratified by total sample: Large (≥200 patients) vs Small (<200 patients).

**S7H. Sample Size: Robotic vs Open — Binary Outcomes**


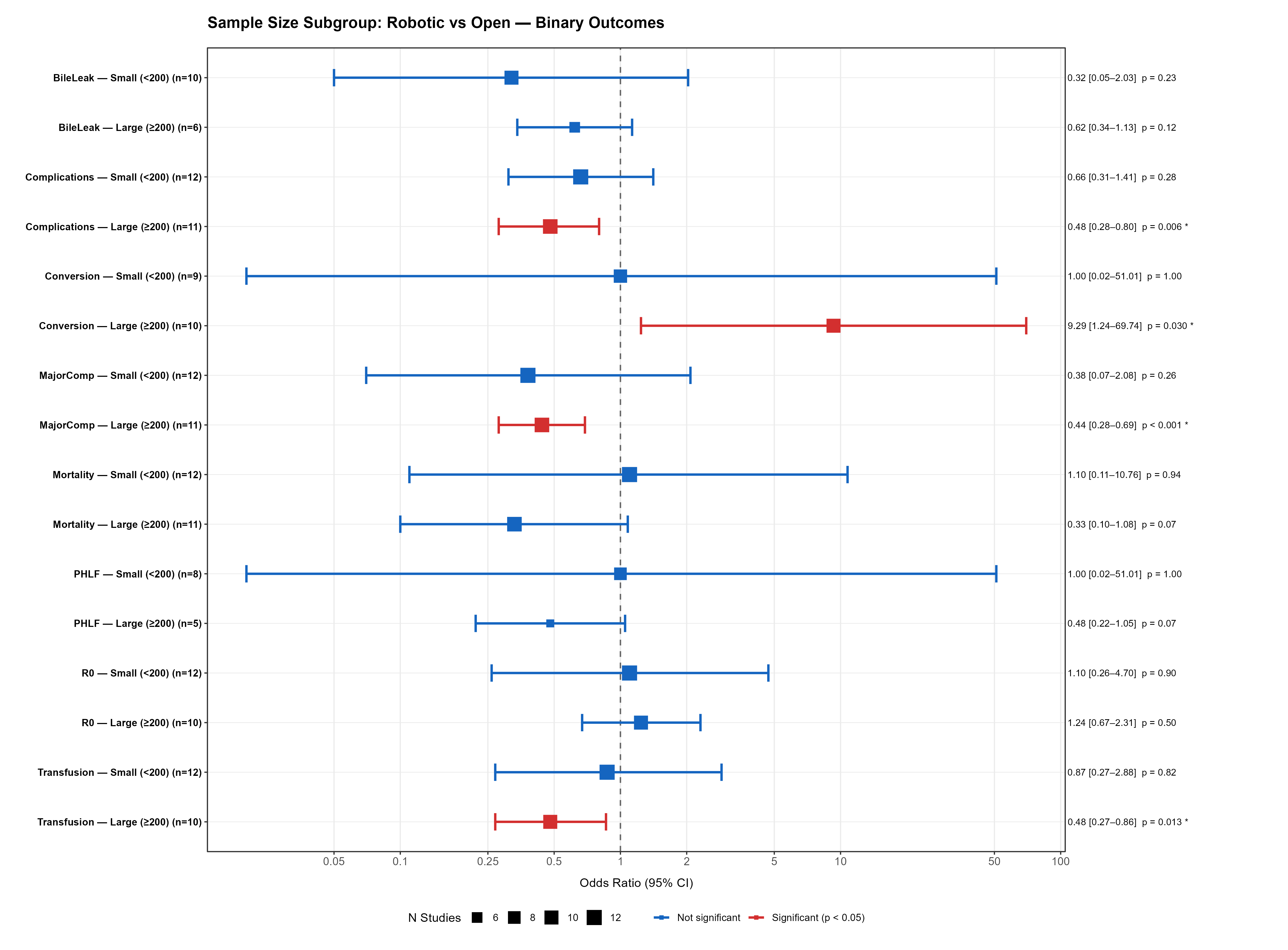


*Supplementary Figure S7H. Robotic vs open outcomes by sample size. Large studies confirmed significant advantages for complications (OR 0.48), major complications (OR 0.44), mortality (OR 0.28), and transfusion (OR 0.48).*

**S7I. Sample Size: Laparoscopic vs Open — Binary Outcomes**


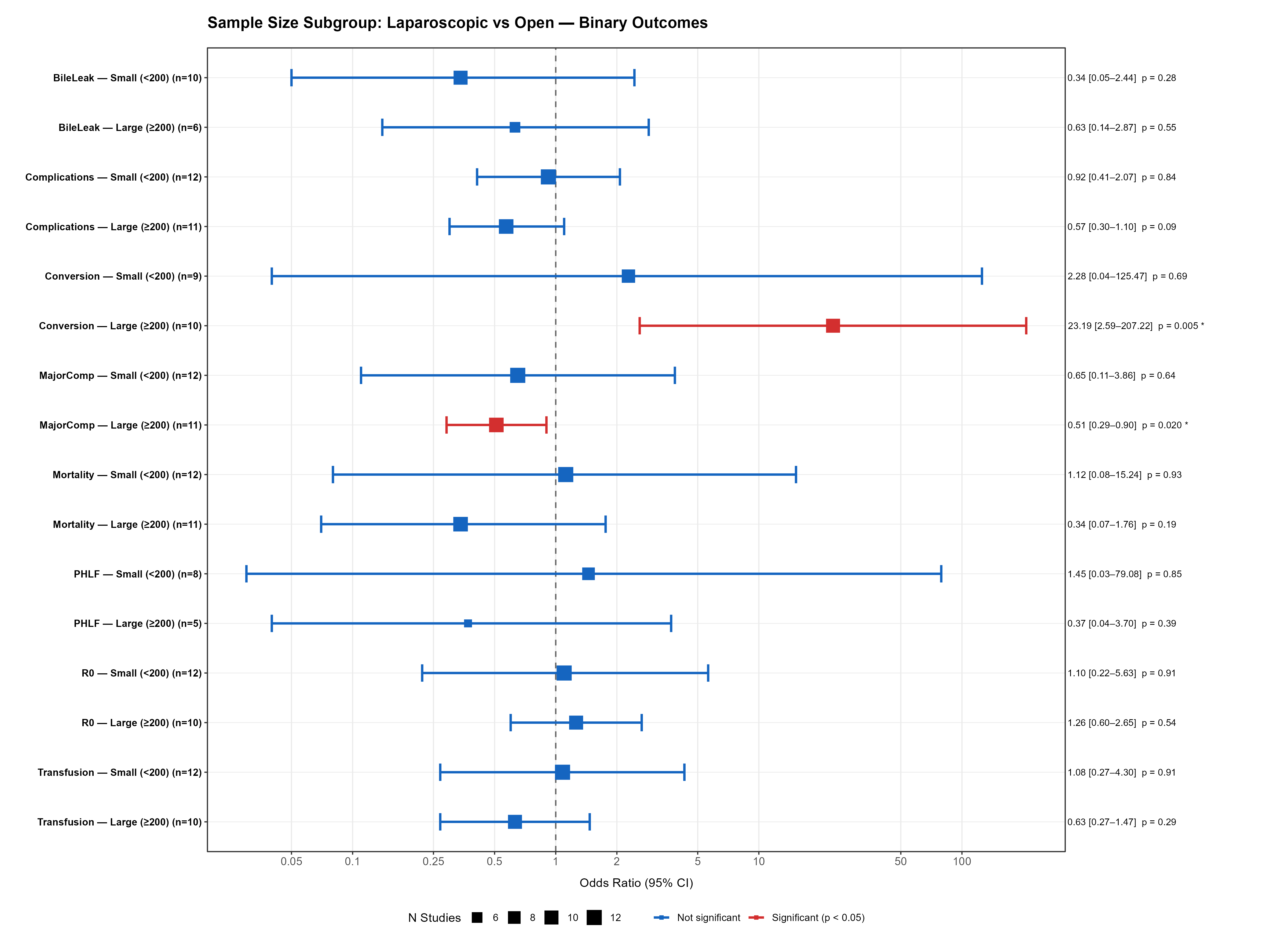


*Supplementary Figure S7I. Laparoscopic vs open outcomes by sample size.*

**SUBGROUP 4: POSTEROSUPERIOR SEGMENTS**

Studies stratified by posterosuperior segment involvement (segments 1, 4a, 7, 8): Yes (n=5) vs No (n=18).

**S7J. Posterosuperior: Robotic vs Open — Binary Outcomes**


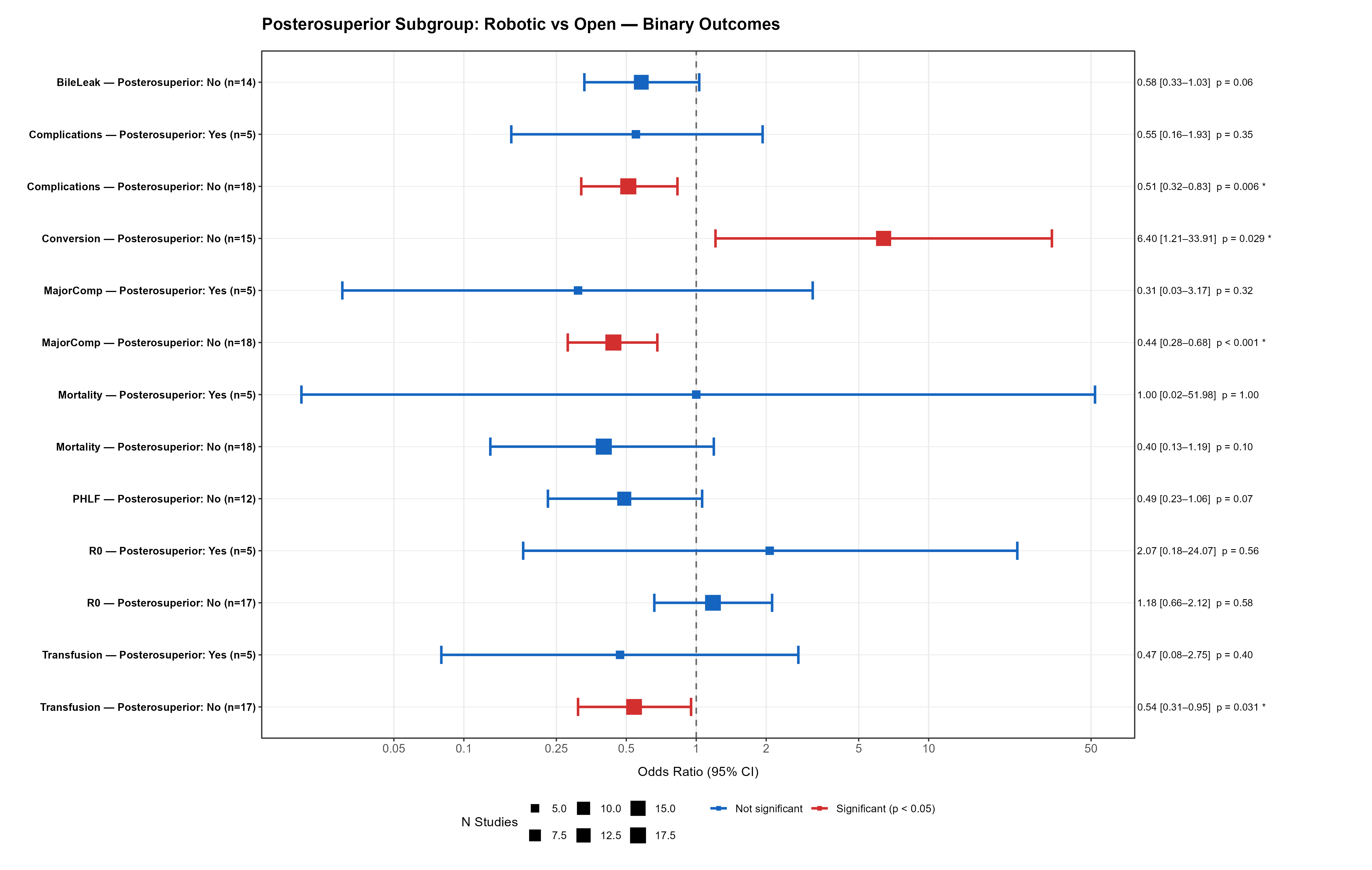


*Supplementary Figure S7J. Robotic vs open outcomes by posterosuperior involvement. Non-posterosuperior studies showed significant advantages for complications (OR 0.51) and major complications (OR 0.44).*

**S7K. Posterosuperior: Laparoscopic vs Open — Binary Outcomes**


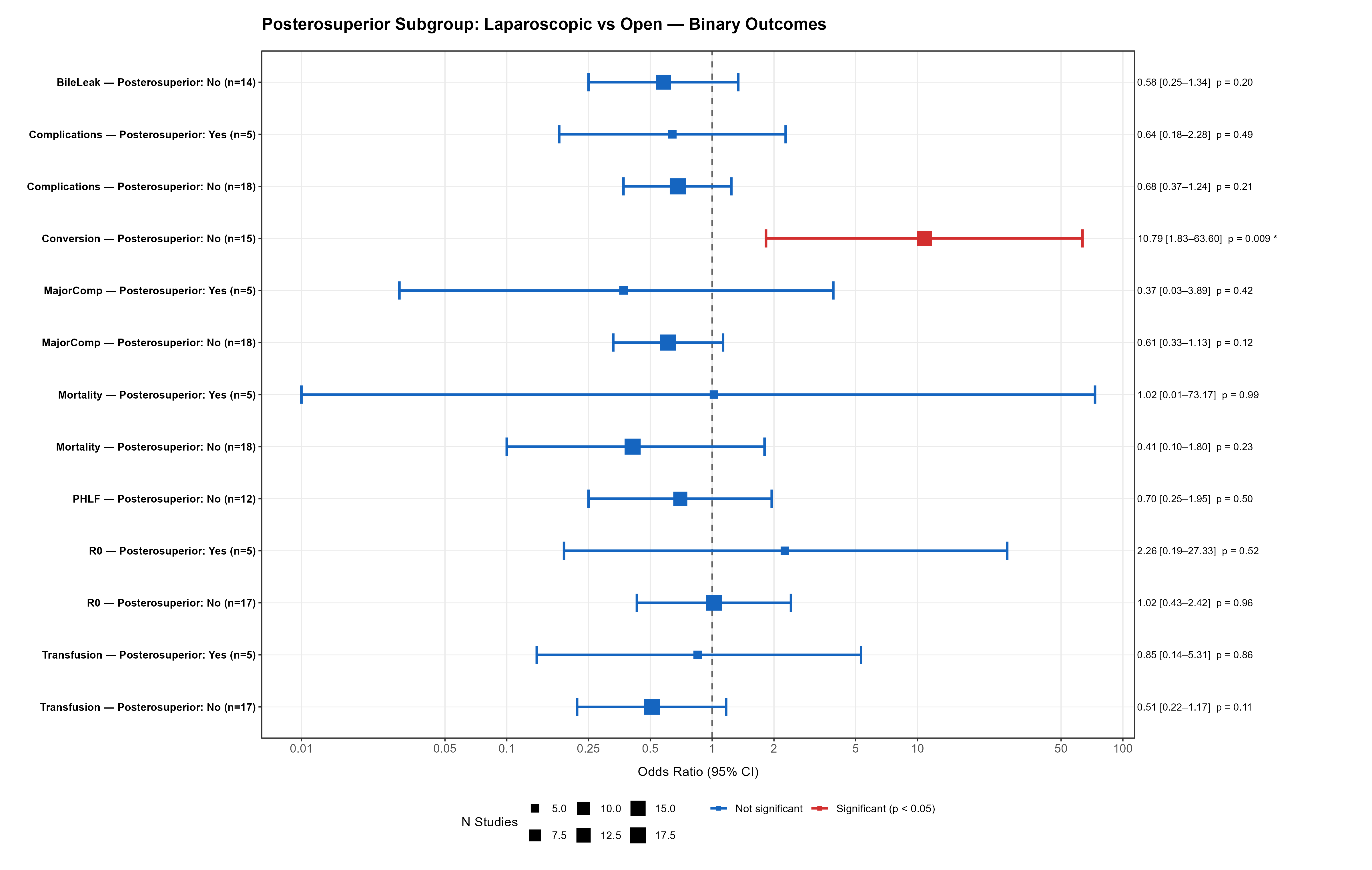


*Supplementary Figure S7K. Laparoscopic vs open outcomes by posterosuperior involvement.*
