# Supplementary material for: Robotic versus laparoscopic versus open hepatectomy for hepatocellular carcinoma: a systematic review and network meta-analysis
Source: J Robot Surg. 2026 Mar 30;20(1):380. doi: 10.1007/s11701-026-03344-2 (PMC13035557; doi:10.1007/s11701-026-03344-2)
Supplement: Supplementary file 4 — Supplementary Material 4 [file 11701_2026_3344_MOESM4_ESM.docx]

# Supplementary Table: Characteristics and References of Included Studies

**Network Meta-Analysis: Robotic vs Laparoscopic vs Open Hepatectomy for Hepatocellular Carcinoma**

## References of Included Studies (n = 23)- Table 1

1. Zhang XP, Chen ZH, Wang K, et al. Short-term and long-term outcomes of robotic hepatectomy versus open hepatectomy for large hepatocellular carcinoma: a multicenter propensity score-matched study. Hepatobiliary Surg Nutr. 2024;13(1):42-55. doi: 10.21037/hbsn-23-256

2. Li H, Chen L, Zhang Y, et al. Efficacy and safety of robotic versus laparoscopic liver resection for hepatocellular carcinoma: a propensity score-matched retrospective cohort study. Hepatol Int. 2024;18(4):1231-1242. doi: 10.1007/s12072-024-10672-4

3. Huang XK, Li Y, Chen J, et al. Robotic versus laparoscopic complex hepatectomy for hepatocellular carcinoma: a propensity score-matched analysis. Hepatobiliary Surg Nutr. 2024;13(2):198-210. doi: 10.21037/hbsn-23-425

4. Zhu P, Liao W, Zhang B, et al. A prospective study using propensity score matching to compare long-term survival outcomes after robotic-assisted, laparoscopic, or open liver resection for patients with BCLC stage 0-A hepatocellular carcinoma. Ann Surg. 2023;278(6):e1187-e1194. doi: 10.1097/SLA.0000000000005967

5. Kato Y, Sugioka A, Kojima M, et al. Initial experience with robotic liver resection: audit of 120 consecutive cases at a single center and comparison with open and laparoscopic approaches. J Hepatobiliary Pancreat Sci. 2023;30(1):72-90. doi: 10.1002/jhbp.1279

6. Giuliante F, Vigano L, De Rose AM, et al. Short and long-term outcomes after minimally invasive liver resection for single small hepatocellular carcinoma: an analysis of 714 patients from the IGoMILS (Italian group of minimally invasive liver surgery) registry. HPB (Oxford). 2023;25(6):679-688. doi: 10.1016/j.hpb.2023.02.005

7. Zhang XP, Chen ZH, Wang K, et al. Short- and long-term outcomes after robotic and open liver resection for elderly patients with hepatocellular carcinoma: a propensity score-matched study. Surg Endosc. 2022;36(11):8362-8373. doi: 10.1007/s00464-022-09273-3

8. Balzano E, Bernardi L, Torzilli G, et al. Implementing a robotic liver resection program does not always require prior laparoscopic experience. Surg Endosc. 2022;36(5):3317-3322. doi: 10.1007/s00464-021-08645-1

9. Pesi B, Moraldi L, Muiesan P, et al. Robotic versus open liver resection in hepatocarcinoma: surgical and oncological outcomes. Updates Surg. 2021;73(3):1085-1092. doi: 10.1007/s13304-021-01013-9

10. Lim C, Salloum C, Tudisco A, et al. Outcomes after 3D laparoscopic and robotic liver resection for hepatocellular carcinoma: a multicenter comparative study. Surg Endosc. 2021;35(7):3540-3549. doi: 10.1007/s00464-020-07829-9

11. Magistri P, Tarantino G, Guidetti C, et al. Laparoscopic versus robotic surgery for hepatocellular carcinoma: the first 46 consecutive cases. J Surg Res. 2017;217:92-99. doi: 10.1016/j.jss.2017.04.027

12. Chen PD, Wu CY, Hu RW, et al. Robotic versus open hepatectomy for hepatocellular carcinoma: a matched comparison. Ann Surg Oncol. 2017;24(4):1021-1028. doi: 10.1245/s10434-016-5638-9

13. Wang Y, Wei Y, Liang B, et al. Liver resection in stage 0-A HCC in segments 7/8: a propensity-matched analysis comparing open, laparoscopic, and robotic approach. Surg Endosc. 2025;39(3):1534-1545. doi: 10.1007/s00464-025-11521-3

14. Bernardi L, Balzano E, Bhogal RH, et al. Recurrence and survival after robotic vs laparoscopic liver resection in very-early to early-stage (BCLC 0-A) hepatocellular carcinoma. Surg Endosc. 2025;39(3):1476-1485. doi: 10.1007/s00464-025-11488-1

15. Duong LM, Wilson RJ, Ajmera M, et al. Outcomes of robotic-assisted liver surgery versus laparoscopic liver surgery for treatment of stage I hepatocellular carcinoma. Cancer. 2022;128(4):807-816. doi: 10.1002/cncr.34007

16. Huang XK, Qin HG, Li Z, et al. Comparing perioperative outcomes of robotic-assisted versus laparoscopic liver resection in patients with hepatocellular carcinoma. J Cancer. 2025;16(3):789-798. doi: 10.7150/jca.104533

17. O'Connell RM, Hoti E, Neary C, et al. Robotic, laparoscopic, and open liver resection for hepatocellular carcinoma: a propensity score matched analysis of perioperative outcomes. Updates Surg. 2023;75(8):2197-2207. doi: 10.1007/s13304-023-01596-7

18. Di Benedetto F, Magistri P, Di Sandro S, et al. Safety and efficacy of robotic vs open liver resection for hepatocellular carcinoma. JAMA Surg. 2023;158(1):46-54. doi: 10.1001/jamasurg.2022.5697

19. Krenzien F, Schmelzle M, Sucher R, et al. Propensity score-matching analysis comparing robotic versus laparoscopic limited liver resections of the posterosuperior segments: an international multi-center study. Ann Surg. 2024;279(2):297-305. doi: 10.1097/SLA.0000000000006075

20. D'Silva M, Cho JY, Han HS, et al. Robotic and laparoscopic right anterior sectionectomy and central hepatectomy: multicentre propensity score-matched analysis. Br J Surg. 2022;109(Suppl 6):znac270. doi: 10.1093/bjs/znac270

21. Nota CL, Woo Y, Raoof M, et al. Robotic versus open minor liver resections of the posterosuperior segments: a multinational, propensity score-matched study. Ann Surg Oncol. 2019;26(2):583-590. doi: 10.1245/s10434-018-6928-1

22. Montalti R, Scuderi V, Patriti A, et al. Robotic versus laparoscopic resections of posterosuperior segments of the liver: a propensity score-matched comparison. Surg Endosc. 2016;30(3):1004-1013. doi: 10.1007/s00464-015-4275-7

23. Lin ZY, Zhang XP, Chen ZH, et al. Short-term outcomes of robotic versus open hepatectomy among overweight patients with hepatocellular carcinoma: a propensity score-matched study. BMC Surg. 2023;23(1):82. doi: 10.1186/s12893-023-01978-3

## Table: Summary of Included Studies

| **No.** | **First Author** | **Year** | **Comparison** | **Sample Size (R/C)** | **Country** | **Design** |
| --- | --- | --- | --- | --- | --- | --- |
| 1 | Zhang XP | 2024 | Rob vs Open | 280/465 | China | Retrospective |
| 2 | Li H | 2024 | Rob vs Lap | 97/244 | China | Retrospective |
| 3 | Huang XK | 2024 | Rob vs Lap | 43/43 | China | Retrospective |
| 4 | Zhu P | 2023 | Rob vs Lap vs Open | 56/56/56 | China | Prospective |
| 5 | Kato Y | 2023 | Rob vs Lap vs Open | 120/451/495 | Japan | Retrospective |
| 6 | Giuliante F | 2023 | Rob vs Lap | 96/618 | Italy | Registry |
| 7 | Zhang XP | 2022 | Rob vs Open | 227/454 | China | Retrospective |
| 8 | Balzano E | 2022 | Rob vs Lap | 40/52 | Italy | Retrospective |
| 9 | Pesi B | 2021 | Rob vs Open | 23/31 | Italy | Retrospective |
| 10 | Lim C | 2021 | Rob vs 3D-Lap | 44/49 | France | Retrospective |
| 11 | Magistri P | 2017 | Rob vs Lap | 22/24 | Italy | Retrospective |
| 12 | Chen PD | 2017 | Rob vs Open | 81/81 | Taiwan | Retrospective |
| 13 | Wang Y | 2025 | Rob vs Lap vs Open | 78/74/61 | China | Retrospective |
| 14 | Bernardi L | 2025 | Rob vs Lap | 68/68 | Switzerland/Italy | Retrospective |
| 15 | Duong LM | 2022 | Rob vs Lap | 123/2926 | USA | Database |
| 16 | Huang XK | 2025 | Rob vs Lap | 53/106 | China | Retrospective |
| 17 | O'Connell RM | 2023 | Rob vs Lap vs Open | 14/14/28 | Ireland | Retrospective |
| 18 | Di Benedetto F | 2023 | Rob vs Open | 106/106 | Italy/USA | Retrospective |
| 19 | Krenzien F | 2024 | Rob vs Lap | 449/898 | International | Retrospective |
| 20 | D'Silva M | 2022 | Rob vs Lap | 127/381 | International | Retrospective |
| 21 | Nota CL | 2019 | Rob vs Open | 31/31 | International | Retrospective |
| 22 | Montalti R | 2016 | Rob vs Lap | 36/72 | Italy/Belgium | Retrospective |
| 23 | Lin ZY | 2023 | Rob vs Open | 104/104 | China | Retrospective |
